# Nature’s Master of Ceremony: The *Populus* Circadian Clock as Orchestrator of Tree Growth and Phenology

**DOI:** 10.1038/s44323-025-00034-4

**Published:** 2025-04-07

**Authors:** Bertold Mariën, Kathryn M. Robinson, Manuela Jurca, Ingrid H. Michelson, Naoki Takata, Iwanka Kozarewa, Pierre A. Pin, Pär K. Ingvarsson, Thomas Moritz, Cristian Ibáñez, Ove Nilsson, Stefan Jansson, Steve Penfield, Jun Yu, Maria E. Eriksson

**Affiliations:** 1 IceLab (Integrated Science Lab), Umeå University, Umeå, Sweden; 2 Department of Mathematics and Mathematical Statistics, Umeå University, Umeå, Sweden; 3 UPSC (Umeå Plant Science Centre), Department of Plant Physiology, Umeå University, Umeå, Sweden; 4 Forest Bio-Research Center, Forestry and Forest Products Research Institute, Hitachi, Ibaraki Japan; 5 UPSC (Umeå Plant Science Centre), Department of Forest Genetics and Plant Physiology, Swedish University of Agricultural Science, Umeå, Sweden; 6 SECOBRA Research, Maule, France; 7 Department of Plant Biology, Swedish University of Agricultural Science, Uppsala, Sweden; 8 CBMR (Novo Nordisk Foundation Center for Basic Metabolic Research), University of Copenhagen, Copenhagen, Denmark; 9 Department of Agronomy, University of La Serena, Ovalle, Chile; 10 Department of Crop Genetics, John Innes Center, Norwich, UK

**Keywords:** Biological techniques, Plant sciences

## Abstract

Understanding the timely regulation of plant growth and phenology is crucial for assessing a terrestrial ecosystem’s productivity and carbon budget. The circadian clock, a system of genetic oscillators, acts as ‘Master of Ceremony’ during plant physiological processes. The mechanism is particularly elusive in trees despite its relevance. The primary and secondary tree growth, leaf senescence, bud set, and bud burst timing were investigated in 68 constructs transformed into *Populus* hybrids and compared with untransformed or transformed controls grown in natural or controlled conditions. The results were analyzed using generalized additive models with ordered-factor-smooth interaction smoothers. This meta-analysis shows that several genetic components are associated with the clock. Especially core clock-regulated genes affected tree growth and phenology in both controlled and field conditions. Our results highlight the importance of field trials and the potential of using the clock to generate trees with improved characteristics for sustainable silviculture (e.g., reprogrammed to new photoperiodic regimes and increased growth).

## Introduction

Even under stable conditions, net ecosystem exchange of contrasting biomes displays diurnal oscillations due to endogenous processes^1,2^. Wood formation is the primary responsible for carbon (C) allocation in woody plants, an important ecosystem C sink^3^. Trees have a seasonal time window for radial growth with unclear drivers, hampering forest productivity and C budget assesments^4–8^. Lockhart^9^ theorized cell expansion and division, allowing radial growth, only occur after exceeding a meristem turgor pressure threshold. Indeed, tree growth is limited by species-specific vapor pressure deficit (VPD) and soil water potential (SWP) ranges explaining most diurnal growth variation^10^. Since VPD and SWP affect transpiration, and transpiration affects water potential and cell turgor, it is implied tree growth is controlled by the C source and tree water relations (i.e., source-limitation hypothesis)^10–13^. Since VPD increases rapidly with daylength, wood growth is inhibited during sunny days^6,10^. Nevertheless, CO_2_ assimilation can still occur, even when cambial activity is restrained by unfavorable conditions^6,14^. Consequently, a temporal decoupling occurs between wood growth (C sink) and C assimilation (C source) which dominate during rainy cloudy days and night-time, and sunny days and daytime, respectively^10^. The number of growth days might more strongly determine annual growth than the growth period (i.e., growing season)^6,15^. Trees’ time window for radial growth is thus determined by the C source (i.e., photosynthesis) and limited by tree water relations (i.e., meristem turgor pressure). In addition, it is suggested growth has greater environmental sensitivities than photosynthesis, and these sensitivities are temporally separated on a species-specific basis explained by species’ capacity to timely regulate physiology^6,14,16^.

The circadian clock (hereinafter “clock”) allows plants to track time and influences plant physiology and phenology (i.e., the timing, duration, and magnitude of life history phases)^6,10,17–26^. Not only does the clock explain species-specific growth patterns (i.e., diurnal growth rate and number of growth days) but also phenological responses (i.e., growth period)^6,23,24^. The clock likely correlates the number of growth days and growth period, determining annual tree growth and C uptake^6,15^. The extent to which the clock affects trees’ life history phases, related ecosystem processes, a species’ environmental sensitivity (i.e., through resonance, matching internal and external rhythms), and diurnal and annual growth pattern remains unclear^27,28^.

The clock is a system of genetic oscillators composed of interconnected transcription-translation negative feedback loops (TTNFLs) reset by signals known as zeitgebers. The mechanism generates cyclic endogenous rhythms adapted to changing environments^29–36^. The clock shows consistent rhythmicity over a broad temperature range (i.e., temperature compensation), is involved in winter hardiness and freezing tolerance, and sensitizes plants to temperature changes^32,37–55^. Many clock-regulated genes co-regulate plant physiology^32,56^. For example, the circadian period shortens with leaf age suggesting involvement of *TIMING OF CAB EXPRESSION 1* (*TOC1*) during leaf senescence^57^. *TOC1* is associated with expression of *FLOWERING LOCUS T* (*FT*) genes, and involved in xylogenesis (i.e., xylem cell formation)^58–60^. Edwards et al.^61^ showed that expression of *LATE ELONGATED HYPOCOTYL 1* and *2* (*LHY1 & 2*; *LHYs*, morning-expressed, light-responsive and repressing *TOC1*) and *TOC1* (evening-expressed and repressing *LHYs*) results in increased biosynthesis of growth regulators cytokinins and auxins during day and night, respectively^62,63^. Through fluctuating Cyclin D3 (CycD3) concentrations, cell division, and proliferation tend to be initiated during the evening, while cell elongation and xylem differentiation tend to occur at night. The clock thus gates (i.e., temporally restricts) DNA replication and mitosis to the night when favorable conditions prevent DNA damage and VPD is below radial growth thresholds^9,19,61,64^.

**Fig. 1:**
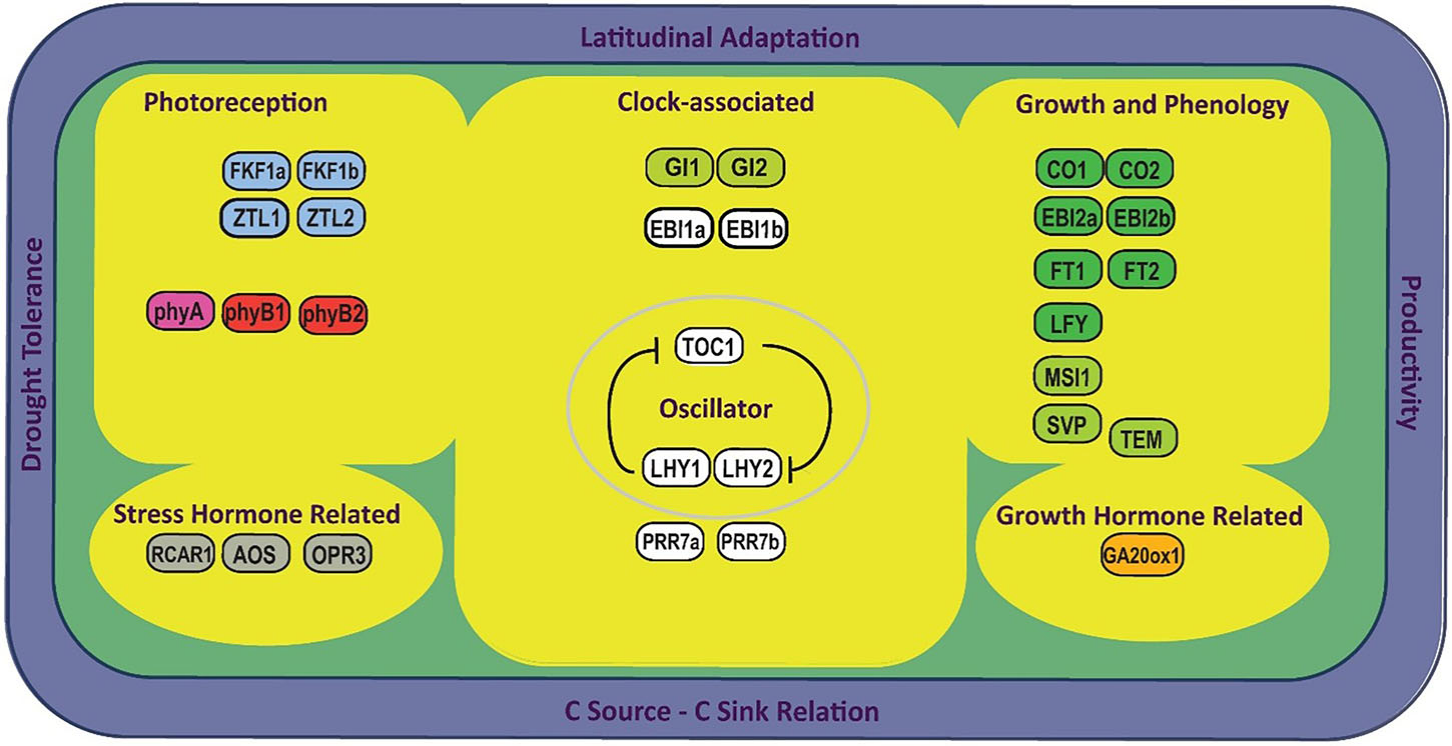
Simplified representation of the *Populus* circadian clock inspired by Bendix et al.^266^, Fogelmark and Troein^267^, and Singh et al.^268^. Relevant proteins comprising or pertaining to the *Populus* clock are schematically grouped and colored-coded based on their major physiological function. Proteins related to the photoreception of far-red, red, or blue light are highlighted in pink, red, or light blue, respectively. Key components of the central clock oscillator are encircled or highlighted in white, while other clock-associated proteins are highlighted in light green. Termination arrows indicate negative effects. Proteins known to regulate growth or phenology are highlighted in dark green, and those linked to stress or growth hormones are represented in gray or orange, respectively. The shaded blue outer perimeter denotes key traits influenced by the clock.

In general, the plant clock is thought to have a conserved architecture^65,66^. Various components make up the *Populus* clock (Fig. 1). Photoreceptors are the most prominent input pathway feature and allow daylight perception. Phytochromes A, B1, and B2 (phyA, phyB1 and phyB2) are dimers allowing daylength tracking^67–74^. Other photoreceptors, like cryptochromes, become phosphorylated upon interaction with blue light^75^. The ZEITLUPE protein family, containing F-box domains and LOV\PAS-domains with KELCH repeats (ZTL and LKP2), their homologs FLAVIN-BINDING KELCH REPEAT, F-BOX1 (FKF1), and phototropins likewise perceive blue light and play an active role in light-dependent protein degradation.

The core clock pathway consists of the TTNFL between *LHY1* and *LHY2* and *TOC1*, with *LHYs* encoding for v-MyB myeloblastosis viral oncogene homolog (MYB) transcription factors important in secondary plant metabolite biosynthesis^76,77^. *TOC1* (aka *PRR1*) belongs to the *PSEUDO-RESPONSE REGULATOR* (*PRR*) gene family^78–80^.

*GIGANTEA* (*GI*) and ZTL are output pathway components and regulate physiological events^81^. In *Arabidopsis thaliana*, ZTL affects gene expression, helps degrades *TOC1* and *PRR5* proteins, and resetting the clock^81–84^. GI, GIGANTEA-LIKE (GIL), and FKF1 proteins interact in a complex mediating degradation of CYCLING DOF FACTOR (CDF) proteins^85,86^. These proteins repress *CONSTANS* (*CO*) and *FT2* gene transcription by binding to *CO* and *FT* promotors^87,88^. *GI* and *GIL* genes regulate photoperiodic response, short-day (SD) induced growth cessation, and bud set^85,88–92^. Two *Populus* CO orthologues exist: *CO1* and *CO2*^59,93^. When *CO* is expressed in *A. thaliana*, it induces *FT* expression under long-day (LD) conditions^94^. FT then travels from phloem to shoot apex and initiates flowering^93,95^. *TEMPRANILLO1* and *2* (*TEM1* & *2*) prevent flowering, and the CO and TEM balance determines FT levels^94,96^. A similar regulatory module may control growth cessation and bud set under SD conditions in *Populus*^59^. *Populus FT* paralogs are expressed at different times in different tissues^97^. For example, *FT1* is involved in release of winter dormancy bud flush, while *FT2* is involved in vegetative growth and bud set^59,85,97,98^. Likewise, *FT1* and *FT2* are mainly expressed in stems and buds, and leaves, respectively^93,99^.

*EARLY BIRD 1* (*EBI1*) and its *Populus* homolog *EBI2* encode, like their *A. thaliana* homologs *NFXL1* and *NFXL2*, for zinc finger proteins with putative transcription factor activity involved in abiotic stress responses^100–102^. EBI may bind to ZTL affecting transcriptional activation of *LHY* and *TOC1*^103^. *EBI*/*NFXL2* thus affects *A. thaliana*’s clock free-running period and speed^32^. Genes like *LEAFY* (*LFY*), *SHORT VEGETATIVE PHASE* (*SVP), MULTICOPY SUPRESSOR OF IRA 1* (*MSI1*), *REGULATORY COMPONENT OF ABSCISIC ACID RECEPTOR 1* (*RCAR1*), *12-OXOPHYTODIENOATE REDUCTASE 3* (*OPR3*) and *ALLENE OXIDE SYNTHASE* (*AOS*) also relate to the clock, growth and phenology^104–109^. The floral meristem identity gene *LFY* controls the inflorescence-to-floral meristem transition^110–112^. Likewise, *SVP* regulates floral meristem specifications and transitions^113^. *SVP* affects temperature-responsive regulation of bud break after vernalization and represses expression of *FT1* and *GA20-OXIDASE1* (*GA20ox1*)^106,114–117^. *GA20ox1* is mainly expressed in photosynthesizing tissue and encodes for an enzyme catalyzing the stepwise conversion of C20 gibberellin growth and developmental hormones (GAs)^116–120^. *MSI1* encodes for a WD-40 repeat protein and is associated with flowering, gametophyte, and seed development^121–127^. Like *SVP*, *RCAR1* represses bud break while affecting *GA20ox1* expression. In addition, *RCAR1* regulates abscisic acid (ABA), which inhibits growth and regulates stress^104,105^. As elucidated in *A. thaliana*, the *OPR3* and *AOS* genes encode enzymes regulating the biosynthesis of the growth and stress resistance hormone jasmonic acid (JA)^107–109,128–133^.

The effects of gene modification regarding plant yield performance are seldom assessed thoroughly in a real-world environment^134^. This meta-study examines the growth and phenology of transgenic trees exposed to both natural and controlled conditions in Sweden.

**Fig. 2:**
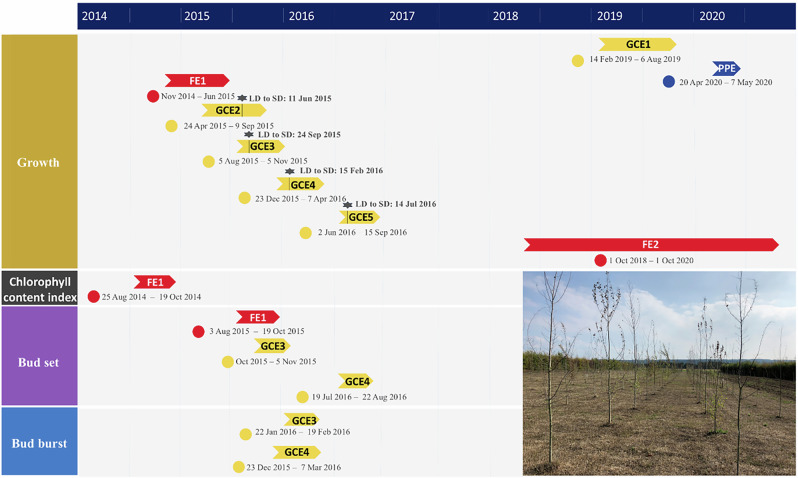
Schematic roadmap of the growth chamber (GCE; yellow), phenotyping platform (PPE; blue), and field (FE; red) experiments done between 2014 and 2020. The length of the arrow and dates indicate the duration of each experiment per variable. The black horizontal line with a green star represents a switch from long-day to short-day conditions. The photo on the bottom right shows FE1 in June 2015.

The growth and phenology of hybrid WT *Populus* T89 (WT^T89^) trees were compared with independently transformed transgenic and WT Elite864012 (WT^Elite^) trees (SI). Their silviculture potential was screened in two large field trials (FEs), with further characterization in five growth chamber experiments (GCEs) and a phenotyping platform experiment (PPE; Fig. 2). To assess statistical significance of growth or phenological changes, we applied generalized additive models (GAMs) allowing determination of when growth or phenological differences become significant and estimation of thresholds at which bud phenological stages occur.

## Results

### Primary and secondary growth effects

**Fig. 3:**
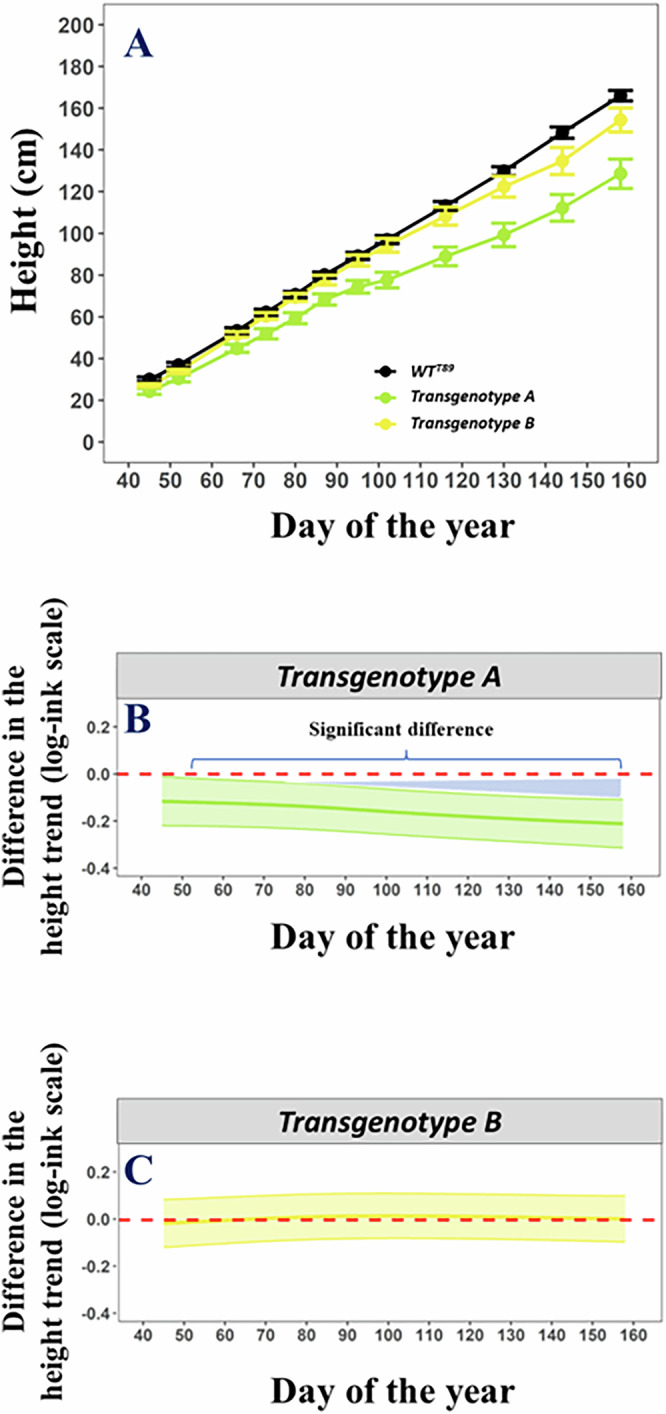
Graphical visualization of a generalized additive model with ordered-factor-smooth interaction smoothers. Example of the primary growth of *P. tremula* L. × *P. tremuloides* trees during growth chamber experiment 1(**A**). The black solid line represents the primary growth of the reference WT^T89^ while the colored solid lines represent the primary growth of two transgenotypes *ztl-5* (A; light-green) and *ztl-7* (B; yellow). Dots and error bars represent mean and standard error values, respectively. Term plots of a generalized additive (mixed) model with ordered-factor-smooth interaction smoothers modeling the mean primary growth of the respective trees during the experiment (**B**, **C**). The horizontal red dotted line represents the mean primary growth of the reference WT^T89^. In contrast, the mean primary growth of *ztl-5* and *ztl-7* are represented by colored solid lines. The difference smooth’s upper Bayesian Wabha/Silverman credible interval (shaded band) is below the horizontal red zero-line in the term plot (i.e., representing the growth of WT^T89^) indicating that the growth of *ztl-5* is significantly (*p* < 0.05) different from the growth of WT^T89^, whereas the growth of *ztl-7* does not differ significantly^135–137^.

The significance of growth changes in transgenotypes (i.e., independent antibiotic-resistant transgenic lines grouped by the same gene construct) is always reported in contrast to WT^T89^ unless indicated (Table 1, SI). The deviation of difference smooth’s Bayesian Wabha/Silverman credible interval above or below the horizontal zero-line in a term plot (i.e., representing WT^T89^ growth) visually indicates significant (*p* < 0.05) differences in transgenotypes’ growth compared to WT^T89^ (Fig. 3)^135–137^.

**Table 1:** The growth and phenology behavior of (RNAi) transgenic *Populus* transgenotypes.

Transgenotype	Significant growth changes compared to WT^T89^		Significant phenological changes compared to WT^T89^			
	Ht.	Diam.	CCI	Bs.	Bb. (ap)	Bb. (lat)
**Photoreceptor-related**						
*pCCR2::LUC PHYA1-1*	- (FE1)	- (FE1)				
*pCCR2::LUC PHYA22-2*	- (PPE), - (FE1)	- (PPE), + (FE1)	+ (FE1)	D (FE1)		
*PHYB-12*	+ (PPE), - (FE2)	+ (FE2)				
PHYB2KO-6*	+ (PPE)					
*ztl-5*	- (GCE1)	- (GCE1)	- (GCE1)			
*ztl-7*	+ (FE1)		+ (GCE1)			
*fkf1-11* ©	- (FE1)	- (FE1)	- (FE1)	A (FE1)		
*lhy PHYA1-32* ©						
*pCCR2::LUC WT (T89)*						
*prr7-5* ©						
*fkf1-10* ©						
*ztl-3*						
**Core clock-related**						
*lhy-10*	- (GCE1), - (PPE), + (FE1)	- (GCE1), + (FE1)		D (FE1)		
*toc1-5*	+ (FE1)	+ (FE1)				
TOC1_OX-7* ©	+ (FE1)					
TOC1_OX-4* ©						
***GI*-related**						
*gi-6* ©	- (PPE)					
*gi-13* ©	+ (FE1)	- (PPE)	+ (FE1)	D (FE1)		
GILOX12*	- (FE2)					
GIOX-12*	- (FE2)					
***AtGA20ox*-related**						
*pMEE1::AtGA20ox1-10* (T89)* ©	- (GCE4), + (FE2)			D (GCE4)	A (GCE4)	A (GCE4)
*pMEE2::AtGA20ox1-2* (T89)* ©	+ (PPE), - (GCE4), + (FE2)	+ (PPE), - (GCE4), + (FE2)		D (GCE4)		A (GCE4)
*pMEE2::AtGA20ox1-11* (T89)* ©	- (GCE4), + (FE2)	+ (FE2)		D (GCE4)	A (GCE4)	A (GCE4)
*pMEE2::AtGA20ox1-5* (T89)* ©	- (GCE4)			D (GCE4)		A (GCE4)
*pMEE1::AtGA20ox1-5* (Elite)* ©	+ (FE2)	- (GCE4), - (FE2)		A (GCE4)	D (GCE4)	D (GCE4)
*pMEE1::AtGA20ox1-8* (Elite)* ©	+ (GCE4)	+ (GCE4)		A (GCE4)	D (GCE4)	D (GCE4)
*pMEE2::AtGA20ox1-5* (Elite)* ©	+ (GCE4)	+ (GCE4)		A (GCE4)	D (GCE4)	D (GCE4)
*pMEE2::AtGA20ox1-6* (Elite)* ©	+ (GCE4)	+ (GCE4)		A (GCE4)	D (GCE4)	D (GCE4)
*pMEE1::AtGA20ox1-3* (T89)* ©				D (GCE4)	A (GCE4)	A (GCE4)
*pMEE1::AtGA20ox1-8* (T89)* ©				D (GCE4)	A (GCE4)	A (GCE4)
*pMEE1::AtGA20ox1-4* (Elite)* ©				A (GCE4)	D (GCE4)	D (GCE4)
*pMEE2::AtGA20ox1-10* (Elite)* ©				A (GCE4)	D (GCE4)	D (GCE4)
**CO/FT-related**						
CO1OX-13*	+ (FE2)					
CO2OX-11*	- (FE2)					
*ft-16*	- (FE1), - (GCE2), + (GCE3), - (FE2)	- (FE1), + (FE2)	- (FE1)	A (FE1), A (GCE3)		
*ft-7*	- (FE2)	+ (FE2)				
*EBI1*-related						
*ebi1-2* ©	+ (GCE1), + (PPE), + (FE1), + (FE2)	+ (GCE1), + (PPE), + (FE1)				
*ebi1-3* ©	+ (GCE1), + (FE1), + (FE2)	+ (GCE1), + (FE1)	+ (FE1)			
*ebi1-14* ©	+ (GCE1), + (FE1), + (FE2)	+ (FE1), - (FE2)				
*ebi2-7* ©	- (PPE), - (FE1)	- (PPE)	- (FE1)			
*ebi2-8* ©	- (PPE)	- (PPE)	- (FE1)			
*ebi2-6* ©	+ (FE1)	+ (FE1)				
**Bud & flowering-related**						
*lfy-4*	+ (FE2)					
*svp-3*	+ (FE2)					
SVPOX-6*	+ (FE2)					
SVPOX-7*	- (FE2)					
*rcar-10*	+ (FE2)					
RCAROX-18*	+ (FE2)					
*msi-1a*						
tem1B-5*						
***OPR3*-related**						
*opr3-15* ©	+ (PPE), + (GCE2)					
*opr3_ft-3* ©	- (FE2)	+ (FE2)				
*opr3_ft-12* ©	- (GCE2)					
*opr3-7* ©						
*opr3-11* ©						
*opr3_ft-7* ©						
***AOS-*related**						
*aos-1* ©				D (GCE3)		
*aos-10* ©	+ (FE2)			D (GCE3)		
*aos-13* ©	+ (FE2)	+ (PPE)		D (GCE3)	D (GCE3)	
*aos_ft-10* ©	+ (GCE3)	- (GCE3)		A (GCE3)		D (GCE3)
*aos_ft-1* ©	- (FE2)	+ (FE2)		A (GCE3)		
*aos_ft-13* ©	- (GCE3), - (FE2)			A (GCE3)		D (GCE3)
**WT-related**						
WT (Elite)*	+ (PPE), + (GCE4)	+ (PPE)		A (GCE4)	D (GCE4)	D (GCE4)
WT (Elite-884056)*						

The name stands for either an experiment done in growth chambers (GCE), the phenotyping platform (PPE), or the field (FE). Ht., Diam., CCI, Bs. and Bb. stand for the height, diameter, chlorophyll content index, bud set, and bud burst, respectively. Measures of significance are symbolized as + and – for positive and negative significant differences between the growth or phenology of a transgenotype and WT^T89^ (i.e., clock mutants with + or – grow significantly faster or slower, respectively. Likewise, clock mutants with D or A display a significant delay or advance, than WT^T89^, respectively). Non-significant results are abbreviated as ns. Transgenotypes introduced here for the first time in the literature are marked with a © symbol. Overexpressing and knockdown transgenotypes are marked by an asterisk or underlined, respectively.

The growth of photoreceptor-related transgenotypes generally differed significantly. The primary and secondary growth of *pAtCCR2::LUC PHYA1-1* (*aPttPHYA*-*1; knockdown construct targeting endogenous PHYA*) and *pAtCCR2::LUC PHYA22-2* (*oatPHYAox-22;* i.e., *overexpressor construct of oatPHYA*) carrying transgene *A.* *thaliana* promoter *COLDCIRCADIANRHYTHMN RNA BINDING2* fused to firefly *LUCIFERASE (AtCCR2pro:LUC*) was usually significantly slower (Figs. S9–S12)^138^. The primary growth of *PHYB-12* and *PHYB2KO-6* with downregulated or knocked out the expression of *PHYTOCHROME B1* and *2* (*PHYB1* and *PHYB2*), respectively, was usually significantly faster (Fig. S5, S6, S51 and S52). The primary growth of *ztl-5* and *7* was significantly slower and faster, respectively (Fig. S1, S2, S9 and S10). The primary and secondary growth of *fkf1-11* was significantly slower (Figs. S9–S12).

The growth patterns of core clock-related transgenotypes were condition dependent as *lhy-10* displayed both significantly slower (in GCEs) and faster primary and secondary growth (in FEs; Figs. S1–S6, S9–S12, S51 and S52). The growth of *toc1-5* and *TOC1_OX-7* was usually significantly faster (Figs. S9–S12).

The primary growth of *gi-6, gi-13*, *GIOX-12*, and *GILOX12* was, except *gi-13*, always significantly slower (Figs. S5–S8, S51 and S52). In contrast, primary and secondary growth of *AtGA20ox1* genotypes under the promoters *AINTEGUMENTALIKE1* (*MEE1*) or *RIBULOSE-1,5-BIPHOSPHATE CARBOXYLASE/OXYGENASE SMALL SUBUNIT* (*RBCS*) cloned from *Eucalyptus grandis* genomic DNA (*MEE2*) was occasionally significantly faster (Figs. S5–S8, S31, S32, S34, S35, S51 and S52)^139,140^. When compared against the growth of WT^Elite^ in GCE4, the primary and secondary growth of *AtGA20ox1* transgenotypes did not consistently grow significantly slower or faster. For example, primary growth of *pMEE1::AtGA20ox1-4*^Elite^ and *pMEE2::AtGA20ox1-10*^Elite^, and *pMEE2::AtGA20ox1-5*^Elite^ and *pMEE2::AtGA20ox1-6*^Elite^ was significantly slower and faster, respectively than WT^Elite^ (Figs. S33, S36, S53 and S56).

We observed contrasting primary growth patterns between *CO1OX-13* and *CO2OX-11* in FE2. The former and latter transgenotypes had primary growth that was significantly faster and slower, respectively (Figs. S51 and S52). Primary growth of *ft-7* and *ft-16* in FE1 and FE2 was usually significantly slower (Figs. S9, S10, S18–S21, S51 and S52). Secondary growth of *ft-7* and *ft-16* in FE2 was significantly faster, but not for *ft-16* in FE1 (Figs. S11, S12, S53 and S54). All *EBI1*-related transgenotypes (*ebi1*) and *ebi2-6* displayed faster primary and secondary growth, except *ebi1-14* in FE2 (Figs. S1, S12, and S51–S64). In contrast, primary and secondary growth of *ebi2-7* and *ebi2-*8 was slower (Figs. S5–S12).

Almost all bud development- or flowering-related transgenotypes had a faster primary growth. From the *12 OPR3*-related transgenotypes, including *opr3-11* and *opr3-7*, only *opr3-15* had significantly faster primary growth. With an *FT* RNAi background, *opr3_ft-3* and *opr3_ft-12*, but not *opr3_ft-7*, explore *OPR3* dependence on *FT* (both *FT1* and *FT2*) and had significantly slower primary growth. *Opr3_ft-3* had significantly faster secondary growth in FE2 (Figs. S5, S6, S18 and S19). A similar pattern was observed in the primary growth of *AOS*-related transgenotypes. *aos-10* and *aos-13*, but not *aos-1*, had significantly faster primary or secondary growth. *aos_ft-10*, *aos_ft-1*, and *aos_ft-13* had both significantly slower as faster primary and secondary growth (Fig. S20, S21, and S51–S54). Primary and secondary growth of WT^Elite^ was usually significantly faster (Figs. S5, S6, S31 and S32).

### Phenological alterations

**Fig. 4:**
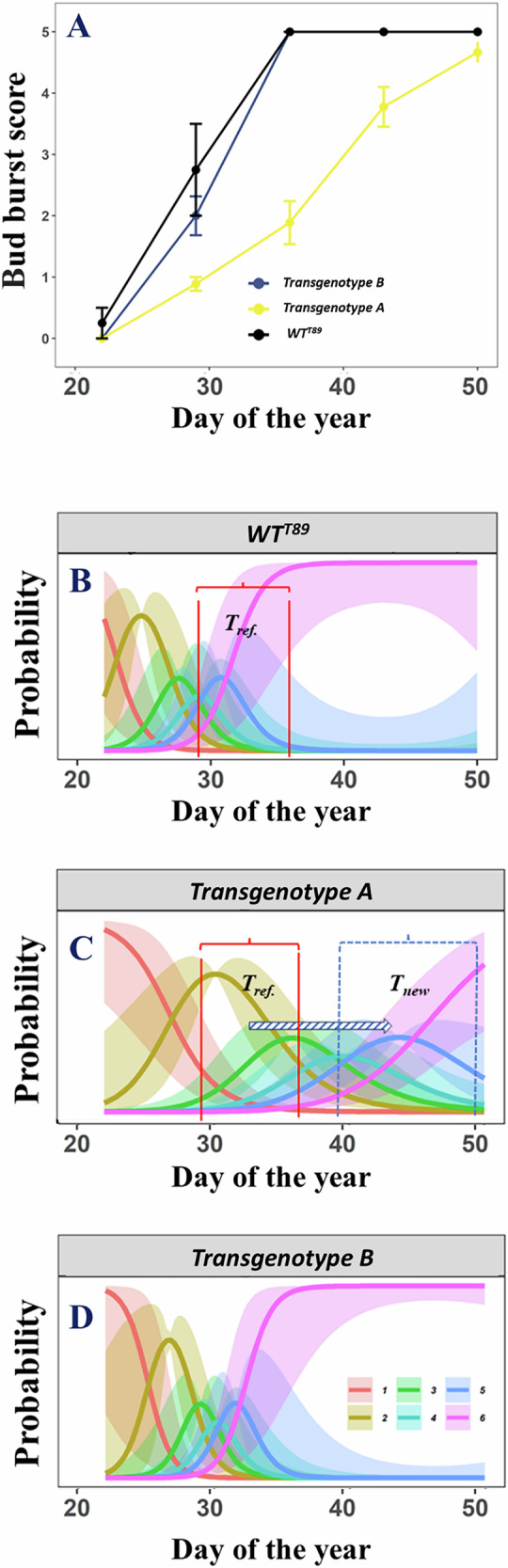
Graphical visualization of a cumulative threshold model. Example of the lateral bud burst scoring of *P. tremula* L. × *P. tremuloides* trees during growth chamber experiment 3. The black solid line represents the bud burst of the reference WT^T89^, while the colored solid lines represent the bud burst of two transgenotypes *aos_ft-10* (A; blue) and *ft-16* (B; yellow; **A**). Dots and error bars represent mean and standard error values, respectively. The lateral bud burst was scored following UPOV^193^ using the following scoring values: dormant buds enveloped by scales (0), swelling buds with diverging scales (1), sprouting buds (2), opened buds with leaves clustered (3), diverging leaves with rolled op blades (4), and completely unfolded leaves (5). Term plots of a generalized additive (mixed) model with ordered-factor-smooth interaction smoothers modeling the bud burst of the respective trees during the experiment (**B**, **C**, **D**). The colored solid lines, given for the reference WT^T89^, *aos_ft-10*, and *ft-16*, represent the predicted probability of having a bud in a specific lateral bud burst stage at a particular moment. The shaded bands around the colored solid lines represent 95% pointwise confidence intervals. The bud burst categories follow UPOV^193^ using the following scoring values: dormant buds enveloped by scales (1; red), swelling buds with diverging scales (2; orange), sprouting buds (3; light-green), opened buds with leaves clustered (4; petroleum-green), diverging leaves with rolled op blades (5; blue), and completely unfolded leaves (6; purple). Let *T*_ref._ represent a rapid increase in the predicted probability of a bud to have completely unfolded leaves. It can be observed that this rapid increase (*T*_new_) is delayed substantially in transgenotype A, as opposed to transgenotype B.

**Fig. 5:**
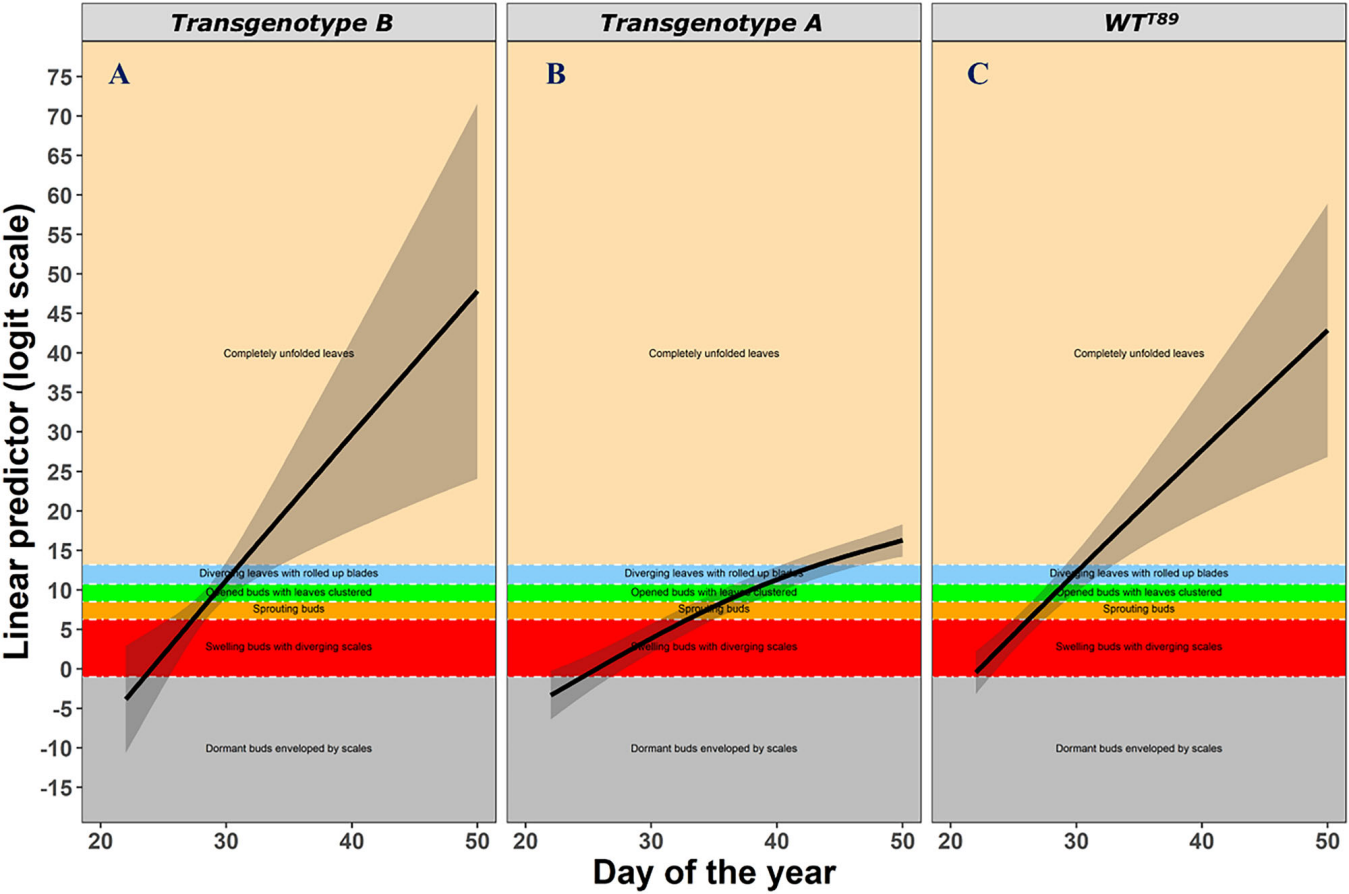
Representation of lateral bud burst development in different transgenotypes using a cumulative threshold modeling framework. Examples of lateral bud burst development in *P. tremula* L. × *P. tremuloides* trees during growth chamber experiment 3. The black solid lines represent the development of the lateral bud burst in two transgenotypes *aos_ft-10* (**A**; subplot B) and *ft-16* (**B**, subplot A), and the WT^T89^ (subplot **C**), through the linear predictor (i.e., the expected value of a latent variable following a logistic distribution). The black shaded bands around these black solid lines represent 95% pointwise confidence intervals. More specifically, the black solid lines show the factor-smooth interaction effect between *Day of the Year* and *Transgenotype* on the linear predictor. Each bud burst category, scored following UPOV^193^, is shown here as colored vertical bars in each subplot: dormant buds enveloped by scales (0; gray), swelling buds with diverging scales (1; red), sprouting buds (2; orange), opened buds with leaves clustered (3; light-green), diverging leaves with rolled op blades (4; blue), and completely unfolded leaves (5; beige). The cumulative threshold model estimates cut points on the latent variable which allows to predict when buds, given a specific transgenotype, are likely to transition from one bud burst category to the next. The first threshold (i.e., the boundary between the first and second bud burst phases; dormant buds enveloped by scales and swelling buds with diverging scales) is by definition always set to −1. In this example, it can be observed that transitions in the lateral bud burst development of transgenotype A occur substantially slower than similar transitions in transgenotype B, or the reference WT^T89^. The lateral bud burst development of transgenotype B, on the other hand, is remarkably similar to the reference WT^T89^.

Differences in chlorophyll content index (CCI), bud set, and bud burst between transgenotypes and WT^T89^ were analyzed using an ordered-factor-smooth interaction approach and cumulative threshold models (Fig. 4)^141,142^. The phenological behavior of transgenotypes is always reported in contrast to WT^T89^ (Tables 1 and 2; Fig. 5; SI).

**Table 2:** Overview of the experiments’ characteristics.

Name	Transgenotypes (n)	Individuals (n)	Sampling				Y_i_
			Start	End	Frequency	LD or SD	
GCE1	8	12	14-Feb-19	6-Aug-19	Weekly	LD	Height
	8	12	14-Feb-19	6-Aug-19	Weekly	LD	Diameter
PPE	26	9	20-Apr-20	7-May-20	Alternate days	LD	Height
	26	9	20-Apr-20	7-May-20	Alternate days	LD	Diameter
FE1	18	18	Nov-14	Jun-15	Alternate months	n/a	Height
	18	18	Nov-14	Jun-15	Alternate months	n/a	Diameter
	18	18	3-Aug-15	19-Oct-15	Weekly	n/a	Bud set
	18	18	25-Aug-14	6-Oct-14	Weekly	n/a	CCI
GCE2	7	9	24-Apr-15	9-Sep-15	Weekly	LD to SD on 11-Jun-15	Height
GCE3	7	9	5-Aug-15	5-Nov-15	Weekly	LD	Height
	7	9	1-Oct-15	5-Nov-15	Weekly	SD	Bud set
	7	9	22-Jan-16	19-Feb-16	Weekly	LD	Bud burst (ap)
	7	9	22-Jan-16	19-Feb-16	Weekly	LD	Bud burst (lat)
GCE4	13	8	23-Dec-15	7-Apr-16	Weekly	LD to SD on 15-Feb-16	Height
	13	8	23-Dec-15	7-Apr-16	Weekly	LD to SD on 15-Feb-16	Diameter
	13	8	19-Jul-16	22-Aug-16	Weekly	SD	Bud set
	10	8	23-Dec-15	7-Mar-16	Weekly	LD	Bud burst (ap)
	13	8	23-Dec-15	7-Mar-16	Weekly	LD	Bud burst (lat)
GCE5	2	12	2-Jun-16	15-Sep-16	Weekly	LD to SD on 14-Jul-16	Height
FE2	34	18	1-Oct-18	1-Oct-20	Every 6 months	n/a	Height
	34	18	1-Oct-18	1-Oct-20	Every 6 months	n/a	Diameter

The name stands for either an experiment done in growth chambers (GCE), the phenotyping platform (PPE), or field (FE). Ap, lat, LD, and SD stand for apical and lateral, and long-day and short-day light conditions, respectively.

*pAtCCR2::LUC PHYA22-2* had significantly delayed bud set and significantly higher CCI values (Figs. S13–S16). The delay in bud set and leaf senescence suggests *pAtCCR2::LUC PHYA22-2* has an extended growth period without faster primary growth. During GCE1, *ztl-5* and *ztl-7* had significantly lower and higher CCI values, respectively (Figs. S15 and S16). *fkf1-11* was observed to have significantly advanced bud set and lower CCI suggesting faster leaf senescence (Figs. S13–S16). Only *lhy-10* had a significantly later bud set from core clock-related transgenotypes (Figs. S13 and S14).

Clock-output-related transgenotypes showed significant phenological differences. *gi-13* had significantly delayed bud set and higher CCI, suggesting a prolonged growth period (Figs. S13–S16). Differences were observed in bud set between lines with ectopic expression of *AtGA20ox1*, and WT^T89^ or WT^Elite^ in the background. The former and latter transgenotypes had significantly delayed or advanced bud set, and advanced or delayed bud burst, respectively (Figs. S37, S38, S41, 42, S45, and S46). Compared to the bud phenology of WT^Elite^, only *pMEE1::AtGA20ox1-4*^Elite^ and *pMEE2::AtGA20ox1-10*^Elite^ differed significantly (Figs. S39, S40, S43, S44, S47, and S48). CO\FT-regulatory-module-related transgenotypes did not differ significantly in their phenology. Only *ft-16* had significantly advanced bud set, delayed apical bud burst, and lower CCI, suggesting a shortened growth period (Figs. S13–S16, S25, and S26).

The phenological differences observed in transgenotypes targeting *EBI* in FE1 are the significantly higher CCI of *ebi1-3*, suggesting slower leaf senescence, and advanced bud set of *ebi2-7* and *ebi2-8* (Figs. S13–S16). The bud set in *aos-1, aos-10*, and *aos-13*, and *aos_ft-1*, *aos_ft-10* and *aos_ft-13* were significantly delayed and advanced, respectively. The apical bud burst in *aos-13*, and lateral bud burst in *aos_ft-10* and *aos_ft-13* was also significantly delayed, suggesting *aos_ft* transgenotype trees have a shortened growth period dependent on *FT*. We observed a significantly delayed apical and lateral bud burst in WT^Elite^ (Figs. S22, S23, S25, S26, S28 and S29).

## Discussion

Clock-associated genes significantly affected tree growth and phenology across various experimental levels. Our findings highlight the potential of modifying clock-associated genes for silviculture. For example, *lhy-10* and *toc1-5* grew faster in the field (likely due to their sped-up core-clock TTNFL) indicating trees can be reprogrammed to new photoperiodic regimes by altering their critical daylength or growth period. By safeguarding DNA replication licensing timing through gating and optimizing resonance between cell cycle phases and external environmental rhythms, *LHY* and *TOC1* regulate growth. Downregulation of these genes may lead to a respective decrease and increase in cold hardiness and tolerance, and critical day length^143–146^.

Increasing the growth of trees cultivated for biofuel may increase bioenergetic yields^147–149^. *EBI1* appears key to tree growth. *ebi1* transgenotypes generally had faster primary and secondary growth. *EBI1*, associated with stress and photosynthesis protection in *A. thaliana*, affects the clock period and expression of *LHY* and *TOC1*^101,103,150^. *EBI* downregulation may even increase primary growth and early flowering^103^. The only phenological difference observed in the *ebi1* transgenotype is that *ebi1-3* had a higher CCI, suggesting slower leaf senescence. The growth functions of *EBI2* appear similar to *EBI1* in *Populus* albeit with expression at different times and tissues^101,103,150^.

Transgenic trees with increased secondary growth have an adaptive advantage under drought conditions and enhanced post-drought recovery^8^. In this light, primary and secondary growth of transgenotypes with *AtGA20ox1* expression were generally faster regardless of background line or reporter-promoter construct. The genetic background of *AtGA20ox1* transgenotype trees affected tree phenology showing the background can be used as an alternative to promotors for altering growth. *MEE1* and *MEE2* did not strongly affect growth. *AtGA20ox1* overexpression increases growth elongation and period, biomass, and fiber length^115,117^. Tree growth even occasionally increased regardless of growth period shortening^117^. The growth period thus appears less relevant than diurnal growth. The background line strongly affected the bud set irrespective of the promoter.

Trees’ photoperiodic growth constraints can be amended. For example, slower primary growth, delayed bud set, and higher CCI values found in *pAtCCR2::LUC PHYA22-2* suggest overexpression of *oatPHYA* results in a prolonged growth period but hampered primary growth^73,151^. Given the lack of differences in *pCCR2::LUC*^*T89*^ growth, we conclude the *pCCR2::LUC* construct did not affect growth. The upregulation of *oatPHYA*, unlike *PHYA1*, not only positively affected leaf and bud phenology but also secondary growth. Downregulation or knocking out *PHYB* in *PHYB-12* and *PHYB2KO-6* generally resulted in trees with faster primary and secondary growth. *PHYB* is involved in day length, temperature perception, and growth cessation, which might explain why the primary growth of *PHYB-12* was slower in FE2 than the PPE^151^. Primary growth of *ztl-5* and *ztl-7* was, respectively, slower and faster in GCE1 and FE1. Primary growth of *fkf1-11* was slower and leaf senescence in *fkf1-11* progressed faster. No changes were observed in *PRR7*^28^.

Modification of clock output-associated genes affected tree growth and phenology. All *GI*-overexpressing transgenotypes had slower primary growth. *gi-13* had delayed bud set and significantly higher CCI, suggesting slower leaf senescence. These results contrast the literature suggesting moderate *GI* downregulation leads to pleiotropic effects in the field^85,152–154^. Trees with *GI* overexpression also displayed delayed growth cessation and bud set under SD conditions in GCEs. *CO1OX-13* and *CO2OX-11* in FE2 had faster and slower primary growth, respectively. Likewise, *FT* downregulation resulted in contrasting growth patterns but generally slower growth. These results suggest *CO1, CO2*, and *FT* expression impact *Populus* growth in natural conditions^59,85,155^. In line with Böhlenius et al.^59^, *ft-16* had significantly advanced bud set, delayed apical bud burst, and lower CCI. Except for *SVPOX-7*, all *lfy*, *svp, SVPOX*, *rcar,* or *RCAROX* transgenotypes had faster primary growth. Our results, suggesting *LFY* downregulation leads to faster primary growth but no effect on bud phenology, contrast the literature stating upregulation of *LFY* generally leads to faster primary growth and advanced flowering^110,111,156^. Due to its role in temperature regulation of flowering and bud burst, *SVP* downregulation was expected to delay bud set and advance bud burst^106^. *RCAR* downregulation likewise advanced bud burst^104,106^. As expected, *msi* transgenotype trees lacked a clear phenotype and WT^Elite^ transgenotype trees generally had a faster primary growth and advanced bud set^125–127^.

We observed positive growth in trees with downregulation of JA biosynthesis-related genes^108,109,128^. *OPR3* downregulation positively affected primary growth but *FT’*s reduced expression overruled this response and generally led to slower primary growth^59^. Primary growth of *aos* and *aos_ft* transgenotypes was generally faster and unclear, respectively. Bud set in *aos_ft* transgenotype was advanced, while apical and lateral bud burst was typically delayed. The implied shortened growth period in *aos_ft* transgenotype trees is expected due to reduction of *FT*s in the background^59^.

Phenological differences were sometimes unexpected or lacking. For example, bud burst in *lhy-10* was later but no phenological differences were observed in *toc1-5*. It remains unclear why *TOC1* overexpression, nor *ZTL* downregulation always altered bud phenology^59,84,143,157,158^.

Tissue-specific clocks exist that differ in their sensitivity to light or temperature compensation mechanism^28,37,159–164^. Roots, for example, have clocks assumed to be slave versions of shoot clocks^159,161,165–167^. Tissue-specific clocks’ sensitivity to environmental changes and importance in tree growth and phenology therefore affects the source-sink limitation paradigm^28,37,159–170^. Dynamic global vegetation models, implicitly assuming plant productivity is C source-limited and underestimating the sensitivity of growth processes to environmental conditions other than photosynthesis, thus need accounting for the clock^16,171–175^.

New *Populus* transgenotypes with promising characteristics were introduced here. *ebi1-2*, *ebi1-3*, or *ebi1-14*, for example, generally had faster primary and secondary growth compared to WT^T89^ suggesting *EBI1* is key to tree growth. Likewise, the effect of different promotors and genetic backgrounds on *AtGA20ox1* genotype trees was tested. Indeed, the latter affected phenology. Transgenotypes, such as *pMEE1::AtGA20ox1-5*^Elite^ or *pMEE1::AtGA20ox1-8*^Elite^, may therefore both grow fast and survive cold climates, confirming the importance of exposing transgenic trees to real-world conditions to strengthen laboratory research^115,134^. It is common forestry practice to transfer southern natural accessions up North. It is now shown core-clock genes’ expression (i.e., *LHY1, LHY2*, or *TOC1*) can be used to reprogram trees biotechnologically to a new photoperiodic regime (i.e., latitudinal adaptation) allowing the growth period of targeted accessions to increase. Statistical learning approaches, such as cumulative threshold models, enrich current tree breeding, cultivation, or forestry practices with substantial benefits for chronosilviculture.

The essential takeaway is that transgenic perturbations of genes comprising or pertaining to the *Populus* clock substantially affected tree physiology across various experimental levels. The clock thus orchestrates tree growth and phenology as if it were Nature’s ‘Master of Ceremony’. Combined with the observation that angiosperm deciduous tree species do not necessarily mirror, or halt, fine- or coarse roots growth in temperature tree species, this confirms the essentiality of Körner’s^16^ proposed paradigm shift in plant growth control^175,176^.

## Methods

### Plant material, constructs, and transformations

Transformable wild type *P. tremula* L. × *P. tremuloides* Michx. CV. T89 plants were used to perform *Agrobacterium tumefaciens*-mediated transformations at the Umeå Plant Science Centre (UPSC)^177,178^. The transformed plants resulted in circa 68 RNAi transgenic lines targeting 26 genes associated with the clock mechanism (Table S2)^179–181^. The trees were propagated using in vitro culture and grown in greenhouses until the experiments started. Independent antibiotic-resistant transgenic lines grouped by the same gene construct are hereafter called a transgenotype. The transformation procedures of 31 transgenotypes previously described in the relevant literature are briefly summarized in Table S3 (SI). The transformation procedures for 39 remaining transgenotypes are discussed in detail below. Table S2 provides information on the design, base pairs, gene models, and primer sequences. Following Ibanez et al.^143^ and Jurca et al.^158^, plasmids were first constructed by obtaining gene-specific fragments from *Populus* cDNA. These fragments were amplified with DNA polymerase and primers with specific primer sequences. The gene fragments were then cloned into an entry vector and, using an enzyme mix, recombined into a plant destination vector^182^. The resulting plasmids were transformed into *A. tumefaciens* C58 strain GV3101 and used to transform WT^T89^ trees^115,180,181^. Independent and stable transgenotypes were selected after exposing the trees to an antibiotic selection marker. Gene expression analysis of *c*. ten transgenotypes of antibiotic-resistant plants was used to select transgenotypes for further analysis.

### Transgenotypes targeting photoreceptor and morning-loop genes

Twelve transgenotypes targeted photoreceptor-related genes. Following Kozarewa et al.^138^, *PHYA1* was antisense inhibited in anti-sense transgenotype *pAtCCR2::LUC PHYA1-1*. pAtCCR2::LUC PHYA22-2* included an over-expression cassette of oat*PHYA* (*construct number). The antisense transgenotype (*pAtCCR2::LUC PHYA1-1*) expresses a full-length *PHYA* cDNA fragment flipped 3′-5′ under the control of the cauliflower mosaic virus (CaMV) 35 s promoter while the oat*PHYA* (i.e., *pAtCCR2::LUC PHYA22-2*) was constitutively expressed by 35 s CaMV^73^. Another promoter-reporter construct of *COLD CIRCADIAN RHYTHMN RNA BINDING 2* (*pAtCCR2*) fused to firefly *LUCIFERASE* (*LUC*) was also introduced to these transgenotypes, as well as in a control transgenotype with only the transgene *pAtCCR2::LUC T89-5*^62^. The transgenotype *PHYB-12* and *PHYB2KO-6* downregulate and knock out the *PHYB* gene, respectively, responsible for a plant’s red-light sensitivity. *PHYB-12*, however, is the result of RNAi, with the gene fragment introduced as two inverted copies with an intron, while *PHYB2KO-6* is generated using CRISPR/Cas9-mediated gene editing (i.e., CRISPR, Clustered Regularly Interspaced Short Palindromic Repeats; Cas9, CRISPR-associated protein 9) following Ding et al.^151,183,184^. *ztl-3*, *ztl- 5* and *ztl-7* are transgenic RNAi transgenotypes constructed following Jurca et al.^158^ which repress *ZTL* expression and hence affect blue-light sensitivity, clock control and the impulse to reset the clock. The transgenotypes targeting the *ZTL* homolog *FKF1*, *fkf1-10* and *fkf1-11*, likewise repress *FKF1* affecting blue-light reception and flowering. *prr7-5*, on the other hand, downregulates *PRR7*, which directly affects the core-loop of the clock. The gene fragments in *fkf1-10*, *fkf1-11*, and *prr7-5* are designed as two inverted copies of the respective amplified gene-specific fragment with an intron in between.

### Transgenotypes targeting core clock-regulating genes

Four transgenotypes included in this study have been described in Ibanez et al.^143^ and Edwards et al.^61^. *lhy-10* is a transgenotype targeting *LHY1* and *LHY2*. This transgenotype represses the expression of *LHY*. Likewise, the expression of *LHY*’s antipodal core clock-regulated gene *TOC1* is downregulated in *toc1-5*. In transgenotypes *TOC1_OX-4* and *TOC1_OX-7*, however, *TOC1* is upregulated. The entire *TOC1* cDNA in these transgenotypes is constitutively upregulated with varying degrees. In all the above transgenotypes, the core loop of the clock is disrupted with severe alterations in the rhythmicity of the clock as a consequence. Both *LHY* and *TOC1* are key to the clock mechanism.

### Transgenotypes targeting genes controlling growth, bud development, and flowering

*GI* is a prominent gene in the output loop of the clock. Similar to Ding et al.^85^, four transgenotypes were constructed targeting *GI* and *GIL*. *gi-6* and *gi-13* were constructed so that *GI* is repressed^185^. The transgenotypes *GIOX-12* and *GILOX-12* upregulate *GI* and *GIL*, respectively. All four transgenotypes, through the effect of *GI* on *CO* and *FT*, are supposed to have altered timings in their growth cessation, bud set, and flowering^89^. Twelve transgenotypes had ectopic At*GA20ox1* expression driven by two specific promoters. This should lead to increased levels of GAs, as the gene coding for the multifunctional enzyme is relevant in synthesizing GAs^120^. These transgenotypes have WT^T89^ or WT^Elite^ in the background. In addition, the transgenotypes *pMEE1::AtGA20ox1-3*^T89^*, pMEE1::AtGA20ox1-4*^Elite^*, pMEE1::AtGA20ox1-5*^Elite^*, pMEE1::AtGA20ox1-8*^T89^*, pMEE1::AtGA20ox1-8*^*Elite*^*, pMEE1::AtGA20ox1-10*^T89^ and *pMEE1::AtGA20ox1-10*^Elite^ have *AINTEGUMENTALIKE1 (AIL1; MEE1* or *pAIL1)* as promotor (i.e., mainly expressed in meristem and young leaves)^139,186^. In contrast, *pMEE2::AtGA20ox1-2*^T89^*, pMEE2::AtGA20ox1-5*^T89^*, pMEE2::AtGA20ox1-5*^Elite^*, pMEE2::AtGA20ox1-6*^Elite^*, pMEE2::AtGA20ox1-10*^Elite^, and *pMEE2::AtGA20ox1-11*^T89^ have *RIBULOSE-1,5-BIPHOSPHATE CARBOXYLASE/OXYGENASE SMALL SUBUNIT* (*RBCS; MEE2* or *pEL1.2*) cloned from *Eucalyptus grandis* genomic DNA as promoter (i.e., mainly expressed in photosynthesizing tissue)^139^.

Like *FKF1*, *GI* and *GIL* in *Populus* may regulate the transcription of *CO*^85,88,90,91^. Two transgenotypes, *CO1OX-13* and *CO2OX-11*, were constructed using RNAi following Hsu et al.^155^ and upregulate either *CO1* or *CO2*, respectively. On the other hand, the transgenotype *tem1B-5* constructed following Castillejo and Pelaz^94^ upregulates *TEM*, which repressed the expression of *FT*. Since the balance between CO and TEM regulates the expression of *FT*, these upregulating transgenotypes will display different bud burst and flowering times compared to the WT^T89^ ^93^. *ft-16* and *ft-7* are two transgenotypes targeting the *FT* gene^59,97,98^. The *FT* and *FT1* gene fragments were introduced in these transgenotypes as two inverted copies with an intron. As a result, the *FT* gene is downregulated, severely affecting the regulation of bud set and flowering by the CO\FT regulatory module^59^.

Following Eriksson et al.^187^, six transgenotypes were constructed with either downregulation of *EBI1* or *EBI2*^188^. *ebi1-2, ebi1-3* and *ebi1-14* repress the former gene, while *ebi2-6, ebi2-7* and *ebi2-8* repress the latter gene. In *A. thaliana*, *EBI* (*PttEBI1* orthologue) is associated with ZTL and hence regulates the transcriptional activity of *LHY* and *TOC1*. It can be expected that these transgenotypes differ significantly in their growth and phenology from the WT^T89^^101,102^.

More transgenotypes target genes involved in bud development and flowering (e.g., *LFY, SVP*, *MSI1*, or *RCAR1*). *lfy-4*, for example, a transgenotype with a fragment of the *LFY* gene, which is involved in the flower-meristem identity, is introduced as two inverted copies with an intron. The *LFY* gene is downregulated in *lfy-4*, which should lead to changes in the timing of tree vegetative growth and flowering. Using a similar design, *SVP* is introduced into *svp-3*. In this transgenotype, *SVP* is also downregulated, supposedly affecting the timing of bud development and the temperature-responsive regulation of flowering. *SVP* is, on the other hand, upregulated in *SVPOX-6* and *SVPOX-7*. In these transgenotypes, the construct consists of a CaMV 35S promoter that drives the expression of the *SVP* cDNA as a translational fusion to an N-terminal human influenza hemagglutinin (HA) sequence. The upregulation of *SVP* in these transgenotypes should likewise affect temperature-responsive regulation of bud break after vernalization. The transgenotype *msi-1A* is a construct for CaMV 35 s promoter-driven upregulation of RNAi to downregulate *MSI1* constructed following Englund^127^. Because of *MSI1* involvement in WD-40 repeat proteins, the trees likely display altered regulation of flowering, and gametophyte and seed development^121–127^. Following Singh et al.^106^, *RCAR1* is downregulated and upregulated in transgenotypes *rcar-10* and *RCAROX-18*, respectively. Although *RCAR1* represses bud break, it also regulates ABA^104,105^. Like the transgenotypes targeting *RCAR1*, twelve other transgenotypes target genes related to JA levels in the trees. Six of these transgenotypes have a fragment introduced of the *OPR3* gene encoding for the OPR3 enzyme regulating the biosynthesis of JAs^87^. *opr3-7*, *opr3-11*, and *opr3-15* simply have the *OPR3* fragment introduced as two inverted copies with an intron leading to downregulation of *OPR3*. *Opr3_ft-3*, *opr3_ft-7* and *opr3_ft-12* have the same design but also have *FT*RNAi in the background, which normally affects the regulation of flowering. Six other transgenotypes target *AOS*, encoding for a cytochrome P450 protein regulating JA biosynthesis, and are also split into three transgenotypes that downregulate AOS (*aos-1*, *aos-10*, and *aos-13*) or downregulate AOS and have *FT*RNAi in the background (*aos_ft-1*, *aos_ft-10* & *aos_ft-13*). Two other wild-type transgenotypes were investigated as alternatives to the reference WT^T89^. Both WT^Elite^ and WT^Elite-884056^, described in Stener and Westin^189^, are of particular interest to clonal forestry practices in Scandinavia.

### Quantitative reverse transcription polymerase chain reactions

This study characterizes transgenotypes related to the *aos, aos_ft, fkf1, opr3, opr3_ft, AtGA20ox, prr7, gi* and *TOC1_OX* transgenotypes found in Table S2 (Figs. S57–S67). Gene expression data for most transgenotypes (SI) was determined using quantitative reverse transcription polymerase chain reactions (RT-qPCR) following Jurca et al.^158^. Eight hours after dawn (ZT8), we sampled leaves from trees grown in a greenhouse under constant light conditions (i.e., 18:6 h light/dark cycles with a light intensity of 250 μmol m^−2 ^s^−1^) and shock froze them in liquid nitrogen. RNA was subsequently extracted from each leaf following the classical cetyltrimethylammonium method of le Provost et al.^190^. After treatment with DNase (TURBO DNA-free kit; Ambion, Austin, US), cDNA was synthesized from 1 μg RNA using an iScript cDNA Synthesis Kit (Bio-Rad Laboratories, California, US). RT-qPCR was performed using a CFX96 Real-Time detection system (Bio-Rad Laboratories), gene-specific primers (SI), three to four biological replicates, and two technical replicates. The relative expression of the genes of interest was normalized against the expression of reference housekeeping genes *ELONGATION FACTOR 1 ALPHA* (*EF1a*) or 18S rRNA. Further calculations were done relative to WT^T89^ or the lowest expressing transgenotype using the 2−ΔΔCT method of Livak and Schmittgen^191^ and Pfaffl^192^. qPCR results were inconclusive for *aos* and *aos_ft* transgenotype trees, as well as *opr3_ft-12*.

### Growth chamber experiments

We conducted five growth chamber experiments (GCE1 - GCE5), measuring the growth and phenology of transgenotypes in growth chambers at UPSC according to standard experimental setups outlined in Ibanez et al.^143^, Edwards et al.^61^, and Jurca et al.^158^ (Fig. 2; Table 2). Following Nilsson et al. ^177^, cuttings of *P. tremula* L. × *P. tremuloides* were first grown in vitro for 4 weeks. The rooted and in vitro-cultivated transgenic trees were then potted in a 3:1 mix of fertilized peat and perlite and grown for another four weeks under constant LD light, temperature, and relative humidity conditions (i.e., 18:6 h light/dark cycles; 18 °C; and 80% relative humidity, respectively) in the greenhouses at UPSC. The light intensity during this period was 200 μmol m^−2 ^s^−1^ (Osram Powerstar HQI-T 400W/D lamps; Osram, Munich, Germany). After the first month, each tree received weekly nutrients (SuperbaS, Supra Hydro AB, Landskrona, Sweden) and water (1.5 l). After a period (Table 2), the trees in GCE2, GCE3, GCE4, and GCE5 were exposed to the same temperature, relative humidity, and irradiance conditions. Still, the light conditions were changed to SD conditions (i.e., 15:9 h light/dark cycles), and dusk time remained unchanged. During each GCE, the primary and secondary growth of the trees was measured weekly. Phenological observations were made on the bud set, and the apical and lateral bud burst in GCE3 and GCE4. The development of the bud set was scored weekly following UPOV^193^ and Ibanez et al.^143^ using the following (opposite) scoring values: a still actively growing shoot and uninitiated bud set (3), initiation of the bud set and cessation of growth (2), formation of the buds (1), and completed bud set (0)^98,143,194^. The development of the apical and lateral bud burst was scored following UPOV^193^ and Ibanez et al.^143^ using the following scoring values: dormant buds enveloped by scales (0), swelling buds with diverging scales (1), sprouting buds (2), opened buds with leaves clustered (3), diverging leaves with rolled up blades (4), and completely unfolded leaves (5).

### Phenotyping platform experiment

The growth of the transgenotypes was also investigated in a trial at the UPSC tree phenotyping platform (WIWAM Conveyor, SMO, Eeklo, Belgium; Fig. 2; Table 2 in SI). In this trial, hereafter called the phenotyping platform experiment (PPE), the trees automatically move around on a conveyor belt, allowing automatic daily watering, fertilization, and monitoring, and recording of growth parameters. The trees in the PPE were first in vitro-cultivated. The trees were then potted in a commercial mix of fertilized peat and soil (Yrkes Plantjord, Weibulls Horto, Hammenhög, Sweden) and grown for another four weeks under constant LD light, temperature, and relative humidity conditions (i.e., 18:6 h light/dark cycles; 18 °C; and 80% relative humidity, respectively). Afterward, the trees were brought into the phenotyping platform to expose them to similar light and temperature conditions (i.e., 18:6 h light/dark cycles and 20:18 °C warm/cold cycles, respectively) but altered relative humidity conditions (i.e., a relative air humidity of 60%). The red-to-far-red light ratio of the artificial LED lights on the phenotyping platform had an approximate value of 0.9 and an irradiance of 150 to 200 μmol m^-2^s^−1^. The soil in the pots was kept automatically at a target humidity of 1.9 (i.e., a value close to the experimentally determined water capacity), meaning that 1.9 l of water is added for 1 kg of dry soil. All trees were watered and fertilized based on the procedure in Wang et al.^195^. After three weeks in the greenhouse, the trees received a weekly dose of 200 ml of 1% Rika-S fertilization (7:1:5 N/P/K; Weibulls Horto, Hammenhög, Sweden). Whilst in the phenotyping platform experiment, the trees were watered twice daily (i.e., according to the target humidity) and fertilized every alternate day with 50 ml of 0.6% Rika-S. In the 7th week, the fertilization dose was increased to 75 ml of 0.6% Rika-S. Additionally, in the 4th and 7th weeks, the trees were treated with Nemasys C insecticide (BASF, Ludwigshafen, Germany). Primary growth of the transgenic trees was measured automatically every alternate day using a light curtain. Secondary growth was also measured every alternate day. However, this was done by automatic photometry of the trees’ sides, top and bottom using three Imperx B4820 RGB cameras. The data were subsequently recorded on a WIWAM computer and made available using the PIPPA web interface (https://pippa.psb.ugent.be/, UGent, Belgium).

### Field experiments

Populus transgenotypes were amplified every fourth week until an adequate number of trees was reached. Subsequently, apical shoots of circa five centimeters were cut under sterile conditions and transferred into sterile plastic jars of one liter containing 130 mL MS growth medium (Duchefa Biochemie, Haarlem, Netherlands), adjusted to pH 5.6. Two shoots were transferred to each jar and exposed to cyclic LD light and temperature conditions (i.e., 18:6 h light/dark and 22:18 °C warm/cold cycles, respectively). After roots developed, each tree of c. 15 cm was replanted in a 1 l plastic pot containing a pre-fertilized 3:1 mix of fertilized peat and perlite (Yrkes Plantjord, Weibulls Horto, Hammenhög, Sweden). The potted trees were transferred to a greenhouse with similar environmental settings and treated as described in Johansson et al.^196^. After a month, the trees were put outside during the daytime to promote acclimatization to field conditions. Subsequently, they were transported and potted at the field site in Våxtorp.

The growth and phenology of the transgenotypes were tested in two transgenic field trials in Våxtorp (56°25′N, 13°47′E; 39 m.a.s.l.; Laholm municipality, Halland county, Sweden; permits 22-2655|12 and 18-3494|16), which are hereafter called field experiment 1 and 2 (FE1 and 2; Fig. 2; Wang et al.^98^ and Table 2). Before the start of the measurements in 2014 (FE1) and 2018 (FE2), the similarly sized and aged trees were planted in a randomized block design of 18 blocks with 3 × 2.3 m of spacing. In June 2014 (FE1), trees were planted in blocks containing 42 trees, including six WT^T89^ individuals and 18 transgenic individuals each representing an unique transgenotype. In October 2016 (FE2), trees were planted in blocks containing 40 trees, including four WT^T89^ individuals and 34 transgenic individuals each representing an unique transgenotype. Each time, a row of WT^T89^ individuals was planted around the randomized field design. Full-grown larch hedges flanked the long sides of the plots. Stem primary and secondary growth was measured in FE1 and FE2 every alternate month and half a year, respectively. The primary growth of each tree was measured from the base to the top axillar bud using a millimeter-scaled measuring pole. Only in FE1, were weekly phenological observations made on the development of the bud set and leaf senescence. As in the GCEs, the development of the bud set was scored using the scoring scheme of UPOV^193^ and Ibanez et al.^143^. The development of leaf senescence was assessed by measuring the chlorophyll content index (CCI) of five randomly chosen leaves per tree with a chlorophyll content meter (CCM 200 plus, Opti-Sciences). Chlorophyll detoxification is the most prominent feature of leaf senescence and allows trees to remobilize nutrients from their leaves towards more vital plant organs^197,198^. Since the CCI is a proxy for chlorophyll concentrations that have, within limits, a close to linear relationship with chlorophyll concentrations, the decline in the CCI can be used as a proxy for the development of the leaf senescence process^199,200^. CCI measurements were made at approximately the same day period and using the same leaf side^199,201,202^. Further details of the number of transgenotypes per experiment, the (approximate) number of tree individuals per transgenotype, and the duration and frequency of the measurement sampling in the GCEs, PPE, and FEs can be found in Table 2.

### Statistical analyses

All growth and phenology data of the trees were analyzed using generalized additive (mixed) models^141,203^. These interpretable models assume that the relationship between the response and explanatory variables can be modeled using (non-)linear smooth functions^203–206^. The use of smoothers, as well as integrated smoothness selection methods, allows GAMs to have an a-priori-unknown but flexible predictor function of which the complexity is completely determined by the data. As a result, GAMs can unravel hidden relationships in the data whilst accounting for numerous statistical obstacles (e.g., overfitting, non-linear relationships, bias/variance tradeoffs)^204,207–209^. Because it is possible to implement random effects or specified correlation structures within the GAM framework, GAMs can also be used to model spatiotemporal data gathered from repeated observations in multiple individuals and locations over time^210,211^. One of the limitations of GAMs, unlike even more flexible statistical models (e.g., GAMLSS; generalized additive models for location, scale, and shape), is that it only focuses on the exponential distribution family and its location parameter *μ* (i.e., alternatively interpretable as the mean). However, few GAM distributions also allow us to model the distribution parameters *σ* (i.e., the shape of the response variable’s distribution; alternatively interpretable as the variance).

### Generalized additive models with ordered-factor-smooth interactions

To test for significant differences between the growth and phenology of each transgenotype and the growth of WT^T89^ in the GCEs, PPE, and FEs, we adopted the GAM with ordered-factor-smooth interaction approach outlined in Wood^140^ and Rose et al. ^212^. In this pair-wise comparison of smoothers approach, separate difference smoothers are generated by the GAM for each factor level minus one reference level. In our study, we therefore used GAMs where the growth or phenology of each transgenotype was modeled as difference smoothers, which compare against a reference smoother (i.e., the growth of WT^T89^; additional models were made with the growth of WT^Elite^ as reference smoother solely for the AtGA20ox1 transgenotypes with WT^Elite^ in the background). Given that all the assumptions of the GAM are met, the deviation of a difference smooth’s upper or lower Bayesian Wabha/Silverman credible interval above or below the horizontal zero-line in a term plot would then visually indicate significant differences in the growth of the transgenotype compared to the growth of WT^T89^ (Fig. 3)^135–137^. R/mgcv’s *summary* function also provides a quantitative indication of the significance of the (ordered) difference smoothers.

To model the primary and secondary growth of the transgenic trees as a function of their covariates, we used the *gam* function in R/mgcv^209,211,213^. Several GAMs with similar construction but using different distributions were tested. The GAMs with Gaussian location-scale distribution (i.e., gaulss) consistently had the lowest Akaike Information Criterion (AIC), and the Gaussian location-scale distribution was therefore selected to be used in the GAMs modeling tree growth^142^. The most suited monotonic link function (i.e., the function linking the distribution parameter to the predictor; “identity”, “log” or “logb” for the mean; “logb” for the variance) varied case by case. For each experiment, and both the mean and variance, primary or secondary growth was modeled with the *primary* or *secondary growth* of the individual tree as the response variable. The fixed covariates of the *primary* and *secondary growth* were the *time* (continuous), *transgenotype* (categorical with up to 34 levels), and tree *individual* (categorical with up to 20 levels). The interaction term was modeled as an (ordered) random factor-smooth interaction between the covariates *time* and *transgenotype*. Random factor-smooth interaction smoothers were chosen because a (difference) smoother was required for each of a large number of transgenotypes and because these smoothers required the same smoothing parameter field^141,214,215^. The dependency among observations of the same tree individual was finally incorporated by using the *individual* as a random intercept (i.e., thus introducing a coefficient for each tree). To reduce overfitting to a minimum, we chose the restricted maximum likelihood (REML) argument as the smoothness selection method^213,216^. To test for significant differences between the development of leaf senescence in each transgenotype and the development of leaf senescence in WT^T89^ during FE1, a Gaussian GAM was made like those used to test tree growth (Eq. 1). The response variable was, in this case, the chlorophyll content index.

The GAMs modeling primary and secondary growth and leaf senescence are formulated in Eq. 1, where both location and scale parameters are considered. ***Y***_***i***_ represents the independent response variable observations on each *individual* indexed by *i*, with *i* = 1,…, n. ***D*** represents the distribution of the response variable, and ***g*** is the monotonic link function relating the predictor ***η*** to the distribution parameters ***µ***_***i***_ (location) and ***σ***_***i***_ (scale)^217–220^. Let ***t*** and ***x*** denote the covariates *time* and *transgenotype*, while ${\boldsymbol{f}}\left({{\boldsymbol{t}}}_{{\boldsymbol{i}}}\right)$, ${{\boldsymbol{f}}}_{{\boldsymbol{1}}}^{{\boldsymbol{o}}}\left({{\boldsymbol{t}}}_{{\boldsymbol{i}}},\,{{\boldsymbol{x}}}_{{\boldsymbol{i}}}\right),$ and ${\boldsymbol{\zeta }}$ represent the smooth function of the covariate ***t***_***i***_, the ordered factor-smooth interaction function of the covariates ***t***_***i***_ and ***x***_***i***_, and the random effect (i.e., the random effect in the mean for each *individual i*), respectively.

1 $$\begin{array}{c}{{\boldsymbol{Y}}}_{{\boldsymbol{i}}} \sim {\boldsymbol{D}}({\rm{\mu }}_{\boldsymbol{i}},{\boldsymbol{\sigma }}_{\boldsymbol{i}})\\ {{\boldsymbol{g}}}_{{\bf{1}}}\left({{\boldsymbol{\mu }}}_{{\boldsymbol{i}}}\right)={{\boldsymbol{\eta }}}_{{\bf{1}}}={{\boldsymbol{f}}}_{{\bf{1}}}\left({{\boldsymbol{t}}}_{{\boldsymbol{i}}}\right)+{{\boldsymbol{f}}}_{{\bf{2}}}^{{\boldsymbol{o}}}\left({{\boldsymbol{t}}}_{{\boldsymbol{i}}},\,{{\boldsymbol{x}}}_{{\boldsymbol{i}}}\right)+{\boldsymbol{\zeta }}\\ {{\boldsymbol{g}}}_{{\bf{2}}}\left({{\boldsymbol{\sigma }}}_{{\boldsymbol{i}}}\right)={{\boldsymbol{\eta }}}_{{\bf{2}}}={{\boldsymbol{f}}}_{{\bf{2}}}\left({{\boldsymbol{t}}}_{{\boldsymbol{i}}}\right)+{{\boldsymbol{f}}}_{{\bf{2}}}^{{\boldsymbol{o}}}\left({{\boldsymbol{t}}}_{{\boldsymbol{i}}},\,{{\boldsymbol{x}}}_{{\boldsymbol{i}}}\right)+{\boldsymbol{\zeta }}\end{array}$$

### Cumulative threshold models

Cumulative threshold models, or more specifically ordered categorical family GAMs, were made to test whether the bud phenology of the transgenic trees differed significantly from the bud phenology of WT^T89^. We tested specifically for differences in the timing of the bud set between the transgenic and WT^T89^ trees grown during GC3, GC4, and FE1. We also tested for differences in the timing of the apical and lateral bud burst between the transgenic and WT^T89^ trees grown during GC3 and GC4. To test for true significance in the differences, we again used the GAM with an ordered-factor-smooth interaction approach. This time, we modeled ordered categorical data. In an additional step, we computed the predicted probabilities of a bud to be in a specific bud set or bud burst stage at each moment in time (Fig. 4)^141,142^. Wood^141^; p. 176, in fact, notes that “the linear predictor [in this kind of ordered categorical family GAMs] provides the expected value of a latent variable according to a logistic distribution. The probability of this latent variable to be in between certain cut-points (i.e., the categories or stages) then provides the probability of the ordered categorical variable to be of the corresponding stage”.

To model the bud set and bud burst of the transgenic trees as a function of their covariates, we again used the *gam* function in R/mgcv^209,211,213^. Due to the ordered and categorized data, GAMs with ordered categorical distribution (i.e., ocat) and “identity” monotonic link function were subsequently made to model the bud set and bud burst. The bud phenology was modeled with the respective *bud set* or *bud burst* for each tree as the response variable. The fixed covariates of the *bud set* and *bud burst* were the *time* (continuous), *transgenotype* (categorical with up to 19 levels), and tree *individual* (categorical with up to 12 levels). As for the GAMs modeling growth, the interaction term was modeled as an (ordered) random factor-smooth interaction smoother between the covariates *time* and *transgenotype*. A reference smoother was introduced in the ordered categorical GAMs. Random factor-smooth interaction smoothers were also chosen because a (difference) smooth was required for each of the large number of transgenotypes and required the same smoothing parameter^141,214,215^. The dependency among observations of the same tree individual was again incorporated using the *individual* as a random intercept. Term plots, generated with the help of the *data_slice* and *fitted_values* functions from R/gratia, provide a visual indication of substantial differences between the predicted probabilities of a bud to be in a specific bud set or bud burst stage at a particular moment^221^. Equations (2) and (3) provide the cumulative threshold models modeling the bud set and bud burst.

2 $$\begin{array}{c}{{\boldsymbol{Y}}}_{{\boldsymbol{i}}} \sim {\boldsymbol{D}}({{\mu }}_{\boldsymbol{i}})\\ {\boldsymbol{g}}\left({{\boldsymbol{\mu }}}_{{\boldsymbol{i}}}\right)={\boldsymbol{\eta }}={{\boldsymbol{f}}}_{{\bf{1}}}\left({{\boldsymbol{t}}}_{{\boldsymbol{i}}}\right)+{{\boldsymbol{f}}}_{{\bf{2}}}^{{\boldsymbol{o}}}\left({{\boldsymbol{t}}}_{{\boldsymbol{i}}},\,{{\boldsymbol{x}}}_{{\boldsymbol{i}}}\right)+{\boldsymbol{\zeta }}\end{array}$$

3 $$\begin{array}{c}{{\boldsymbol{Y}}}_{{\boldsymbol{i}}} \sim {\boldsymbol{D}}({{\mu }}_{\boldsymbol{i}})\\ {\boldsymbol{g}}\left({{\boldsymbol{\mu }}}_{{\boldsymbol{i}}}\right)={\boldsymbol{\eta }}={{\boldsymbol{f}}}^{{\boldsymbol{o}}}\left({{\boldsymbol{t}}}_{{\boldsymbol{i}}},\,{{\boldsymbol{x}}}_{{\boldsymbol{i}}}\right)+{\boldsymbol{\zeta }}\end{array}$$

### Model assumptions

The interpretation of the GAMs, their output, and the extent of the potentially significant differences between their smoothers depends on the degree to which their underlying parametric assumptions are met and reported^222–225^. Following Zuur et al.^226^ these are, in order of importance, the homogeneity, normality, concurvity, nonlinear dependency, and temporal dependency of the model residuals. The assumption of independent and identically distributed residuals (IID) combines aspects of heterogeneity and dependency and requires assessment. The zero adjusting assumption is not applicable to this study. Failure to meet, or deviations from, the model assumptions might increase type I or II errors or affect the effect size estimation or its significance^227,228^. However, a quantitative analysis of the potential error is not straightforward.

All model residuals were extracted using the *residuals* functions from R/mgcv^140^. Several other functions from the same package provided a first indication of the degree to which many of the parametric assumptions are met. The *summary* function provides approximate p-values on a significant trend in the smoothers and an idea of the model deviance. The model deviance generally proved to be high, adding consideration to the notion that although the hypothesis complexity may increase (i.e., the number of parameters), one can still have low bias and variance (i.e., the interpolation threshold) and that infinite overparameterization can be preferable to any finite number of parameters^229–231^. Deviations from homogeneity or normality of the residuals could be assessed visually using four diagnostic plots of the normalized quantile residuals provided by the *gam.check* function^232^. The homoscedasticity in the residuals was also further investigated using the *check1D*, *l_densCheck* and *l_gridCheck1D* functions in R/mgcViz^233^. In light of the central limit theory and the rather small sample sizes typical for biological experiments, we used the provided histograms and quantile-quantile plots to assess whether the residuals were normally distributed. The *gam.check* function, which provides the k-indices and *p*-values, was used to check the required basis dimensions for the smoothers^234^. Low *p*-values in combination with k-indices lower than one would suggest that the basis dimension of the smoothers was too low. The *shapiro.test* (Shapiro-Wilk test), *ad.test* (Anderson-Darling test) and *cvm.test* (Cramer-Von-Mises test) functions in the R/base, R/nortest, and R/goftest packages were also used to test whether the model residuals followed a normal distribution^235–237^. Non-linearity in the model residuals was assessed with the *bdsTest* function (Brock–Dechert–Scheinkmand and LeBaron statistic test) in R/fNonlinear^238^. The IID characteristics of the model residuals were investigated by testing for white noise in the error vector using the *whitenoise.test* function in R/normwhn.test and the standard *Box.test* function (Ljung-Box test)^239–241^. Residual temporal autocorrelation was visually assessed using the *acf* and p*acf* functions in R/mgcv. Only the raw residual ACF and pACF plots could be provided. Indications of temporal autocorrelation were further investigated by looking for trends or difference stationarity in the residuals. Four tests were used from the R/urca package^242^. The KPSS (Kwiatkowski-Phillips-Schmidt-Shin), PP (Phillips-Perron), ADF (Augmented-Dickey-Fuller), ERS (Elliot-Rothenberg and Stock Point Optimal), and ADF-GLS tests for the presence of a unit root (i.e., a stochastic trend) in a time series test, were performed using the *ur.kpss*, *ur.pp*, *ur.df* and *ur.ers* functions, respectively^243–245^. The additional NP (Ng and Perron) unit root test, giving valid results even when an unknown ARMA process is present, was implemented using the *CADFtest* function in R/CADFtest^246^. The potential need to add autoregressive or moving average orders to the GAMs was tested by running the *auto.arima* function in R/forecast^247^. Tests were also done to characterize the data further, given that the parametric model assumptions were, according to a strict interpretation, often not met. The Hurst coefficient for long-range dependence and randomness in a system was performed using the *WhittleEst* function from the R/longmemo package^248–252^. The skewness (i.e., the asymmetry around the mean of the probability distribution) and kurtosis (i.e., the magnitude in which the tails of a distribution differ from the tails of a normal distribution) were calculated using the *skewness*, *kurtosis* and *describe* functions in the packages R/e1071, R/sur and R/pscyh^253–255^. Indicative for a “good” model fit is a mean, variance, skewness, kurtosis, and Filliben correlation coefficient of 0,1,0,3 and 1, respectively^256^. The modality of the data distributions was tested using the *dip.test* function (i.e., unimodal test) in R/diptest, bimodality_amplitude function (i.e., bimodal test) in R/modes and modetest function (i.e., multimodal test) in R/multimode^257–259^. Graphical output was made mainly using R/ggplot2 and R/dplyr^260–265^.

## Supplementary information

Supplementary information

Supplementary information

Supplementary information

## Data Availability

Extra information is provided in the supplementary information files. These include also data and code.
