# Supplementary material for: Nature’s Master of Ceremony: The Populus Circadian Clock as Orchestrator of Tree Growth and Phenology
Source: NPJ Biol Timing Sleep. 2025 Apr 7;2:16. doi: 10.1038/s44323-025-00034-4 (PMC11976295; doi:10.1038/s44323-025-00034-4)
Supplement: Supplementary file 1 — Supplementary information [file 44323_2025_34_MOESM1_ESM.pdf]

Table of Contents

Supplemental Supplemental Figures **3**
Growth Chamber Experiment 1 (GCE1) 3
Primary growth (Figures S1 - S2) 3
Secondary growth (Figures S3 - S4) 5
Phenotyping Platform Experiment (PPE) 7
Primary growth (Figures S5 – S6) 7
Secondary growth (Figures S7 – S8) 9
Field Experiment 1 (FE1) 11
Primary growth (Figures S9 – S10) 11
Secondary growth (Figures S11 – S12) 13
Chlorophyll content index (Figures S13 – S14) 15
Bud set (Figures S15 – S17) 17
Growth Chamber Experiment 2 (GCE2) 20
Primary growth (Figures S18 – S19) 20
Growth Chamber Experiment 3 (GCE3) 22
Primary growth (Figures S20 – S21) 22
Bud set (Figures S22 – S24) 24
Apical bud burst (Figures S25 – S27) 27
Lateral bud burst (Figures S28 – S30) 30
Growth Chamber Experiment 4 (GCE4) 33
Primary growth (Figures S31 – S33) 33
Secondary growth (Figures S34 – S36) 36

Bud set (Figures S37 – S40) 39
Apical bud burst (Figures S41 – S44) 43
Lateral bud burst (Figures S45 – S48) 47
Growth Chamber Experiment 5 (GCE5) 51
Primary growth (Figures S49 – S50) 51
Field Experiment 2 (FE2) 53
Primary growth (Figures S51 – S53) 53
Secondary growth (Figures S54 – S56) 56
Quantitative reverse transcription polymerase chain reactions (RT-qPCR) (Figures S57 – S76) 59
Supplemental Tables (Tables S1 – S3) **70**
References **74**

Supplemental Figures

Growth Chamber Experiment 1 (GCE1)

Primary growth

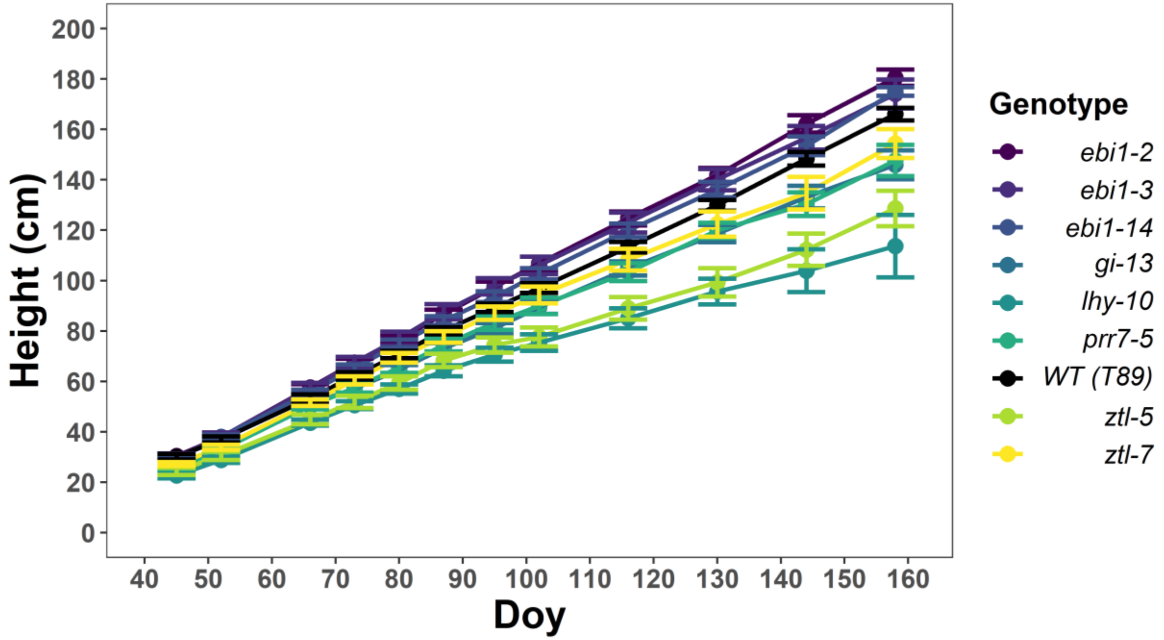

Figure S1. The primary growth of *P. tremula* L. x *P. tremuloides* trees during GCE1 under constant long-day (LD) light, temperature, and relative humidity conditions (i.e., 18:6 hours light/dark cycles; 18 °C; and 80% relative humidity, respectively). The black solid line represents the primary growth of the reference WT^{T89} while the colored solid lines represent the primary growth of independently RNAi transformed transgenic lines. Dots and error bars represent mean and standard error values, respectively. Doy is the abbreviation for day of the year. Doy counts started on the 1st of January 2019.

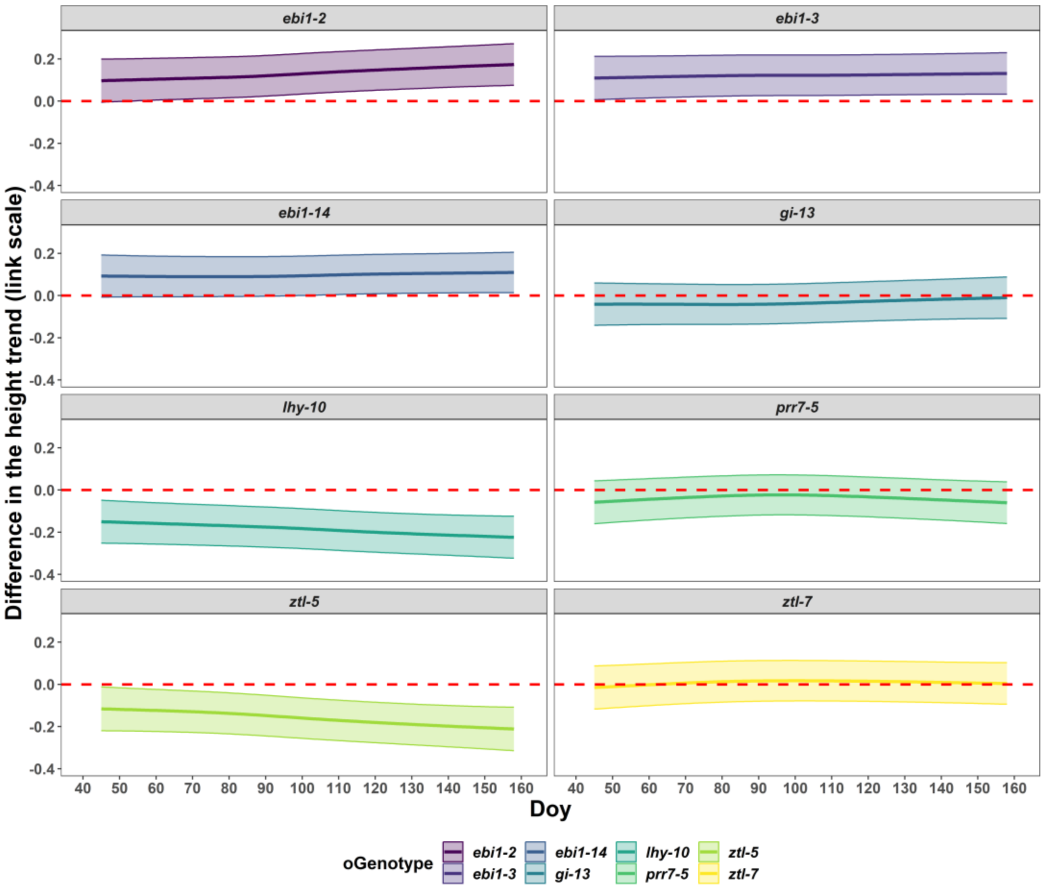

Figure S2. Term plots of the generalized additive (mixed) models with ordered-factor-smooth interaction smoothers modeling the mean primary growth of *P. tremula* L. x *P. tremuloides* trees during GCE1 under constant long-day (LD) light, temperature, and relative humidity conditions (i.e., 18:6 hours light/dark cycles; 18 °C; and 80% relative humidity, respectively). The mean primary growth of the reference WT^{T89} is represented by the horizontal red dotted line. In contrast, the mean primary growth of the independently RNAi transformed transgenic lines is represented by colored solid lines. The shaded bands around the smooth terms represent Bayesian Wabha/Silverman credible intervals. Doy is the abbreviation for day of the year. Doy counts started on the 1st of January 2019.

Secondary growth

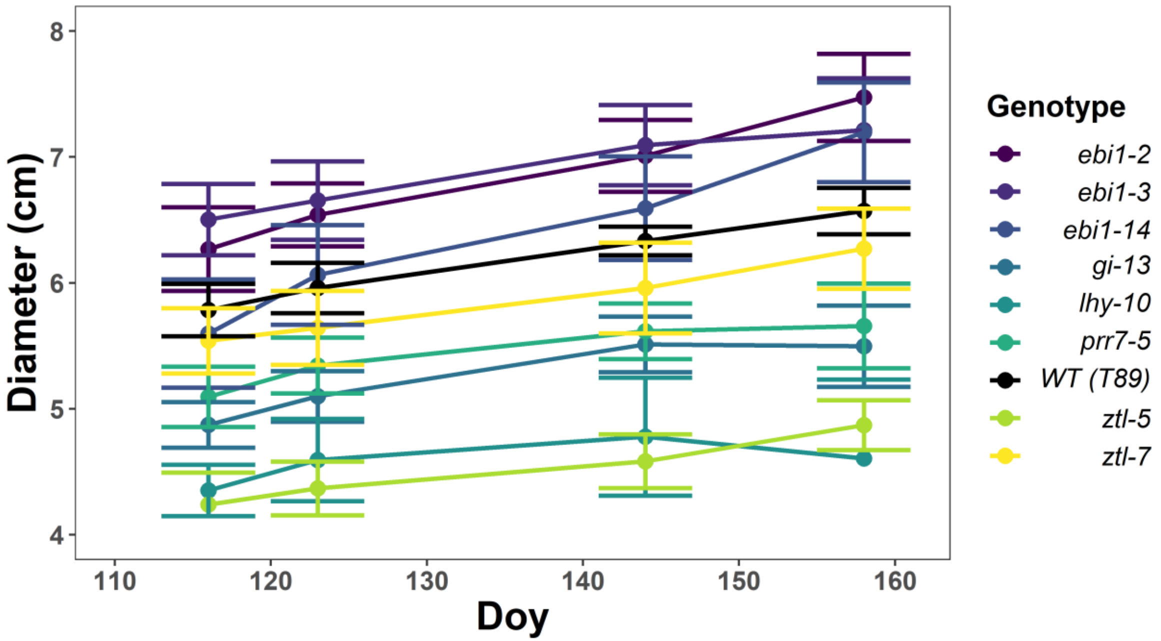

Figure S3. The secondary growth of *P. tremula* L. x *P. tremuloides* trees during GCE1 under constant long-day (LD) light, temperature, and relative humidity conditions (i.e., 18:6 hours light/dark cycles; 18 °C; and 80% relative humidity, respectively). The black solid line represents the secondary growth of the reference WT^{T89} while the colored solid lines represent the secondary growth of independently RNAi transformed transgenic lines. Dots and error bars represent mean and standard error values, respectively. Doy is the abbreviation for day of the year. Doy counts started on the 1st of January 2019.

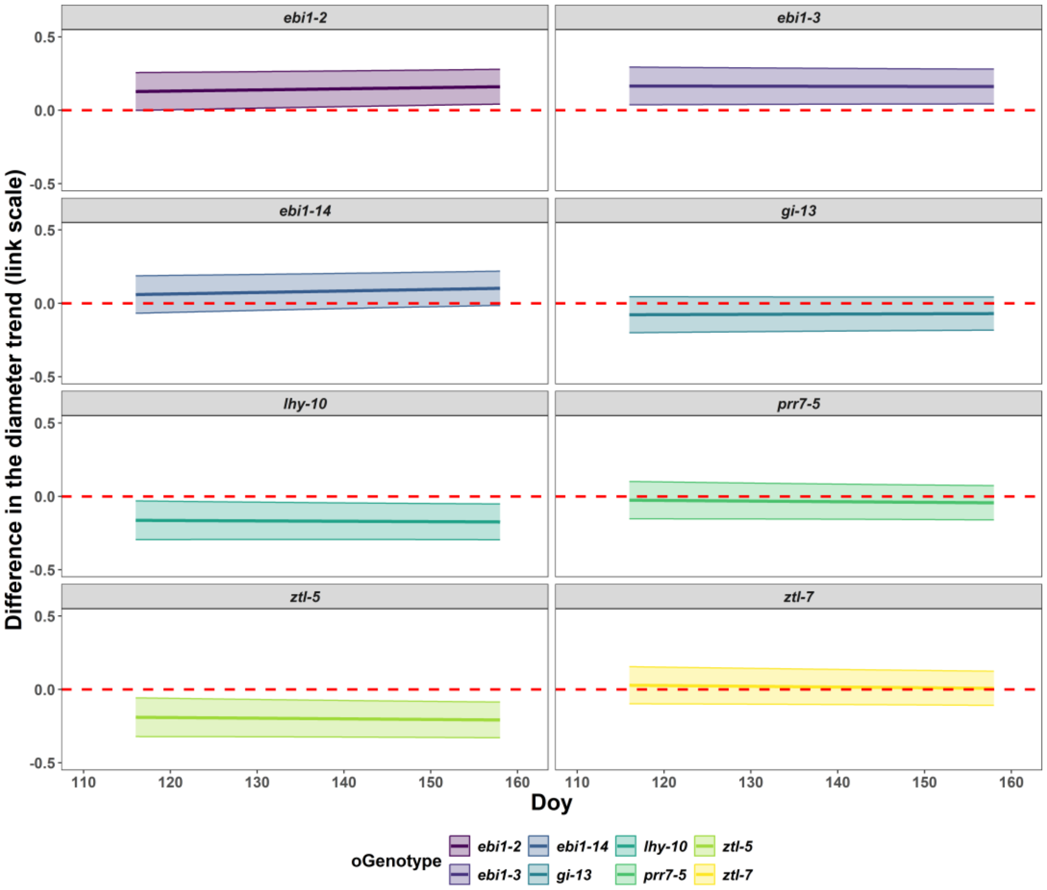

Figure S4. Term plots of the generalized additive (mixed) models with ordered-factor-smooth interaction smoothers modeling the mean secondary growth of *P. tremula* L. x *P. tremuloides* trees during GCE1 under constant long-day (LD) light, t'emperature, and relative humidity conditions (i.e., 18:6 hours light/dark cycles; 18 °C; and 80% relative humidity, respectively). The mean secondary growth of the reference WT^{T89} is represented by the horizontal red dotted line. In contrast, the mean secondary growth of the independently RNAi transformed transgenic lines is represented by colored solid lines. The shaded bands around the smooth terms represent Bayesian Wabha/Silverman credible intervals. Doy is the abbreviation for day of the year. Doy counts started on the 1st of January 2019.

Phenotyping Platform Experiment (PPE)

Primary growth

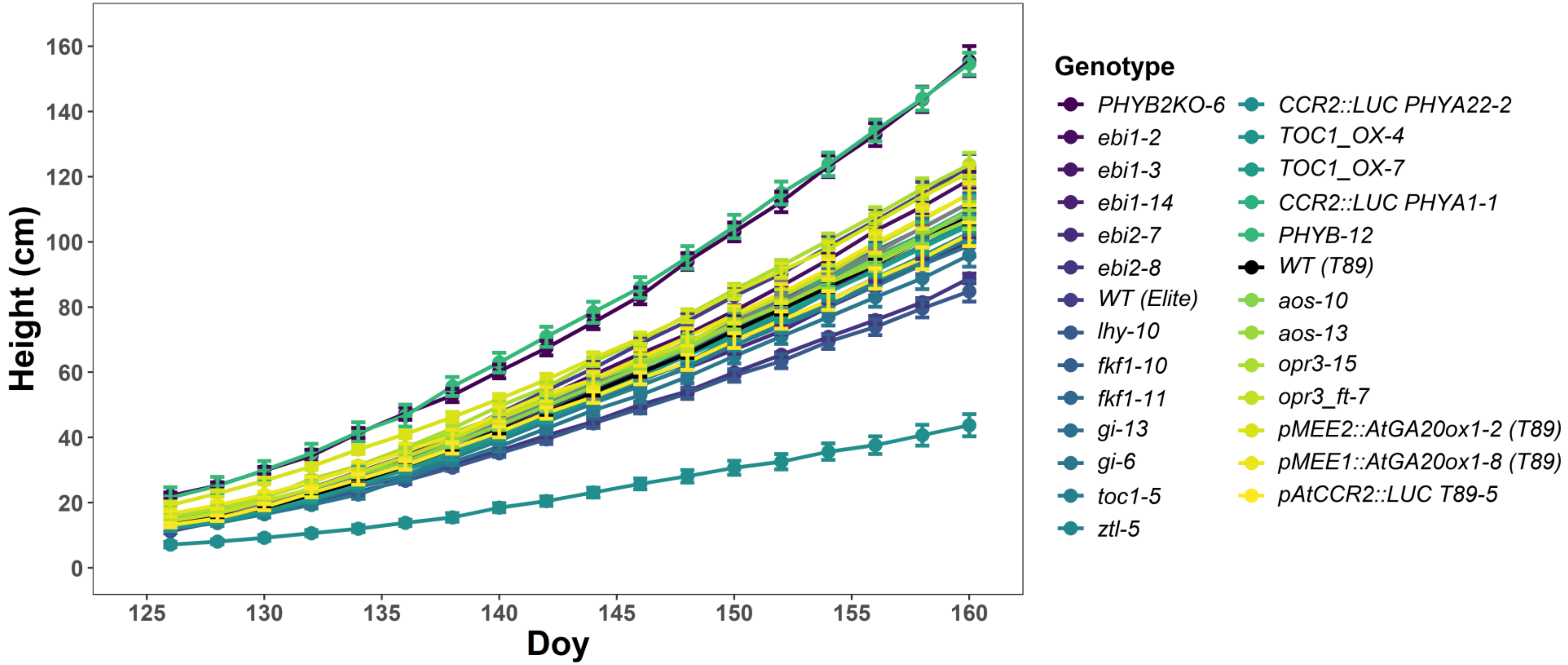

Figure S5. The primary growth of *P. tremula* L. x *P. tremuloides* trees during the PPE under constant long-day (LD) light, temperature, and relative humidity conditions (i.e., 18:6 hours light/dark cycles; 20:18 °C warm/cold cycles; and 60% relative humidity, respectively). The black solid line represents the primary growth of the reference WT^{T89} while the colored solid lines represent the primary growth of independently RNAi transformed transgenic lines. Dots and error bars represent mean and standard error values, respectively. Doy is the abbreviation for day of the year. Doy counts started on the 1st of January 2020.

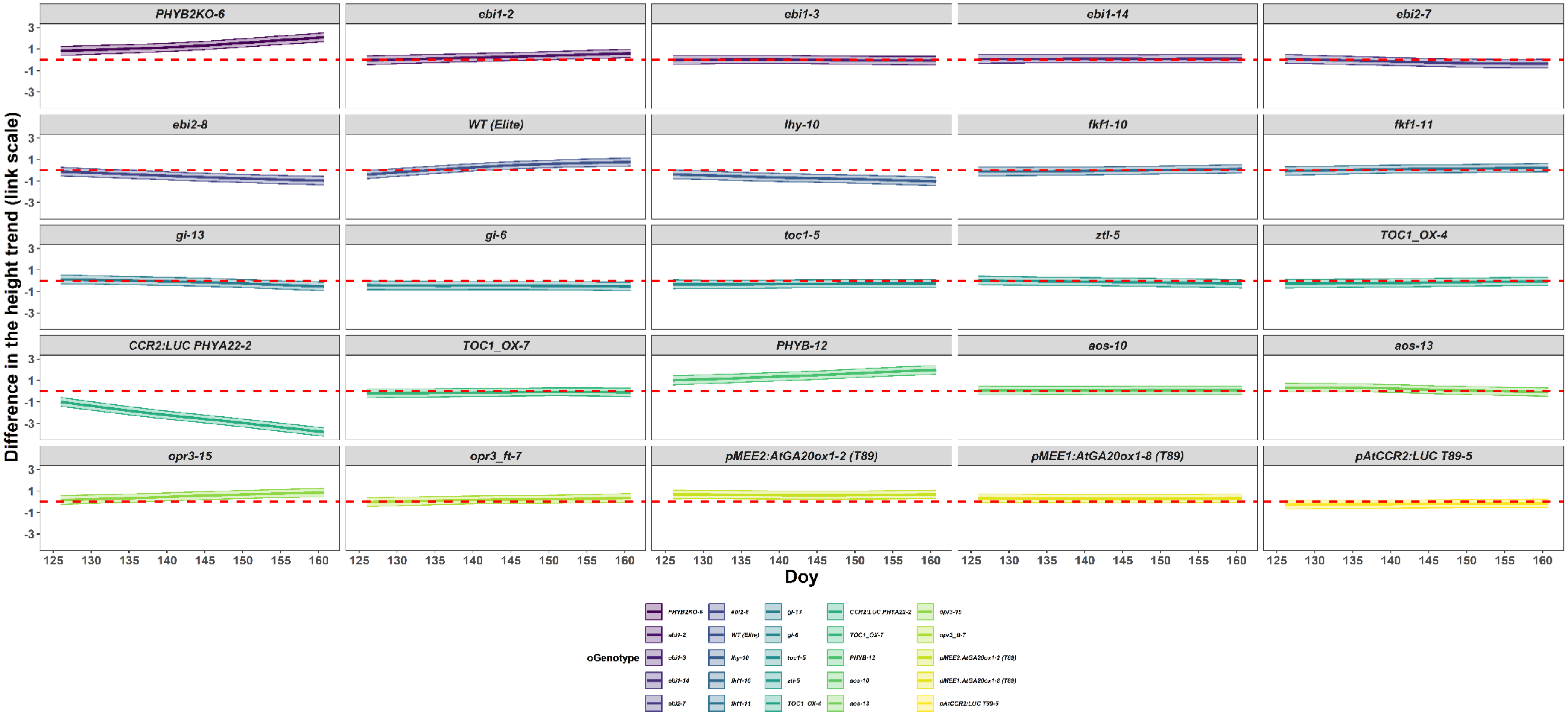

Figure S6. Term plots of the generalized additive (mixed) models with ordered-factor-smooth interaction smoothers modeling the mean primary growth of *P. tremula* L. x *P. tremuloides* trees during the PPE under constant long-day (LD) light, temperature, and relative humidity conditions (i.e., 18:6 hours light/dark cycles; 20:18 °C warm/cold cycles; and 60% relative humidity, respectively). The mean primary growth of the reference WT^{T89} is represented by the horizontal red dotted line. In contrast, the mean primary growth of the independently RNAi transformed transgenic lines is represented by colored solid lines. The shaded bands around the smooth terms represent Bayesian Wabha/Silverman credible intervals. Doy is the abbreviation for day of the year. Doy counts started on the 1st of January 2020.

Secondary growth

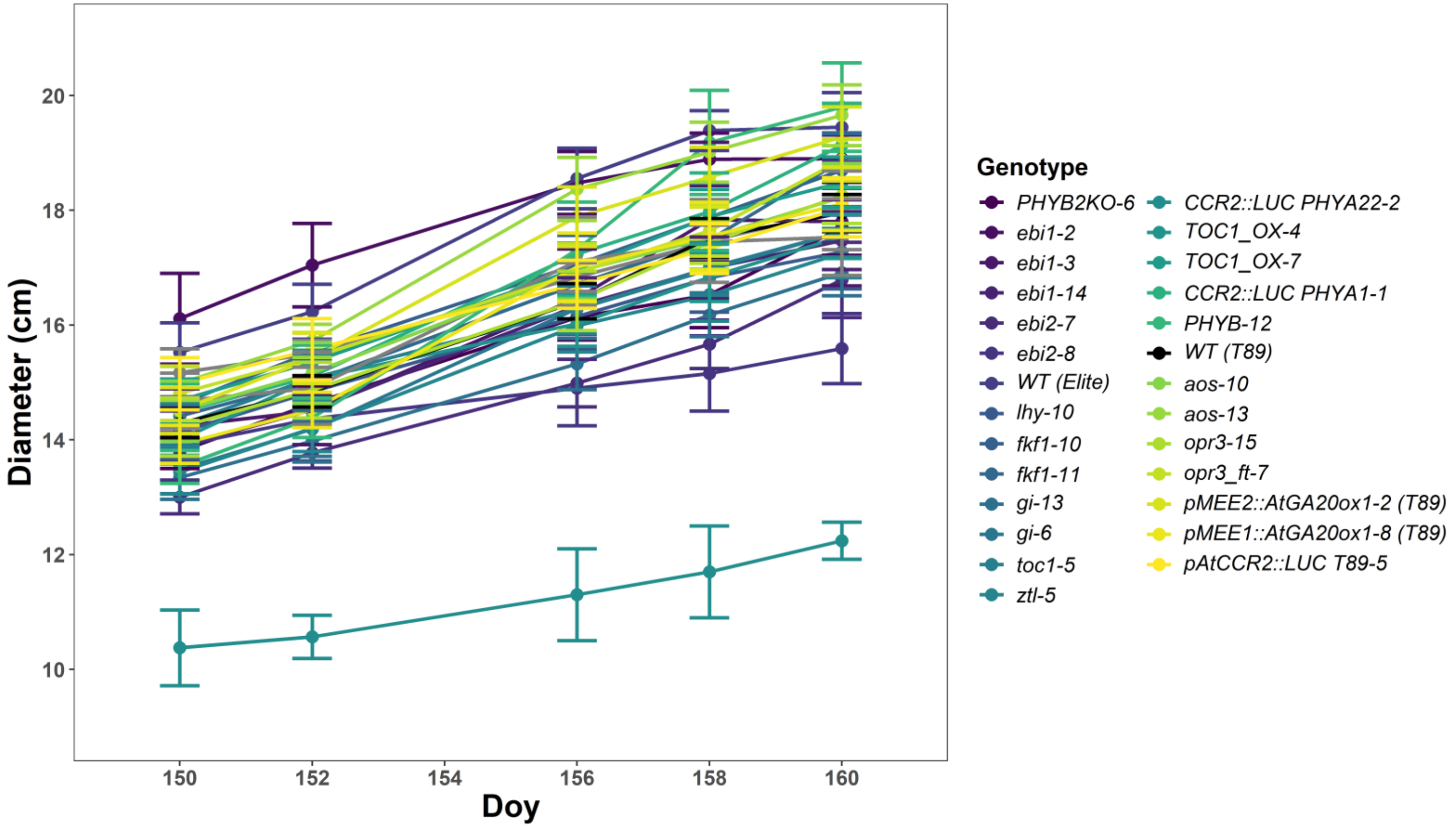

Figure S7. The secondary growth of *P. tremula* L. x *P. tremuloides* trees during the PPE under constant long-day (LD) light, temperature, and relative humidity conditions (i.e., 18:6 hours light/dark cycles; 20:18 °C warm/cold cycles; and 60% relative humidity, respectively). The black solid line represents the secondary growth of the reference WT^{T89} while the colored solid lines represent the secondary growth of independently RNAi transformed transgenic lines. Dots and error bars represent mean and standard error values, respectively. Doy is the abbreviation for day of the year. Doy counts started on the 1st of January 2020.

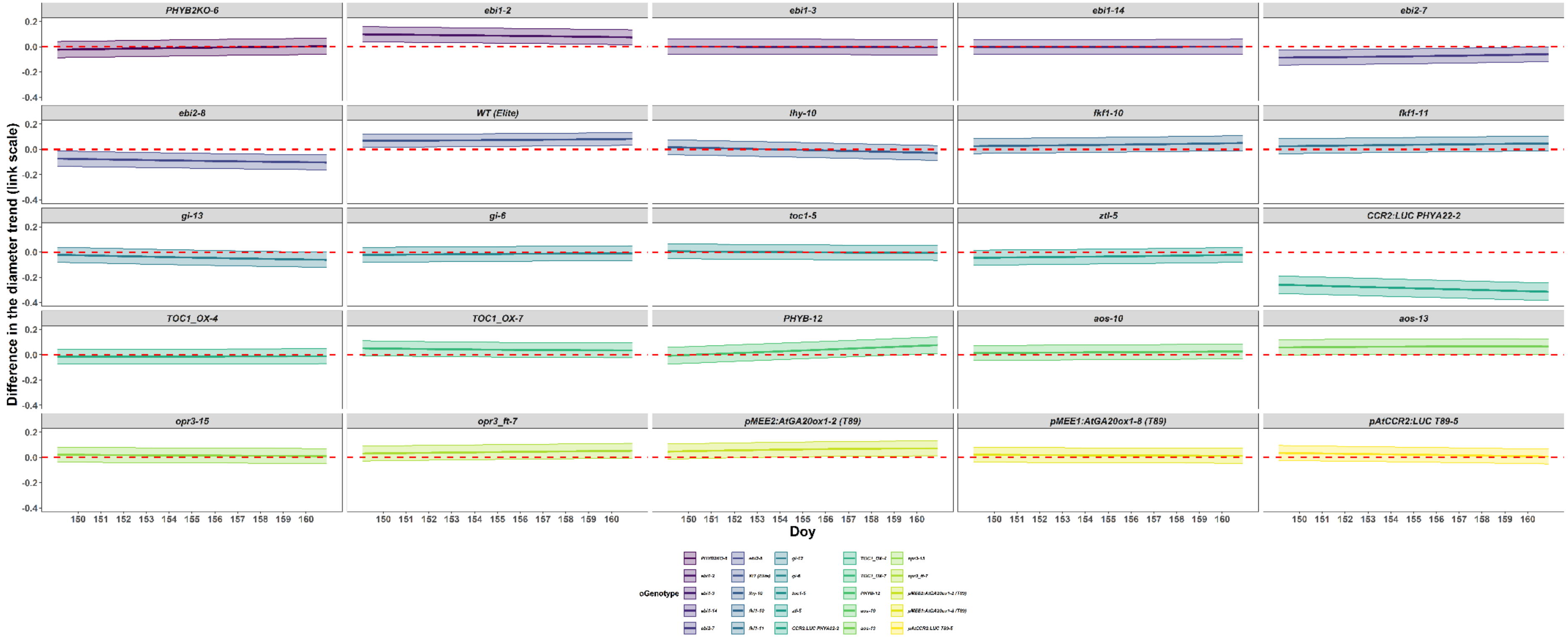

Figure S8. Term plots of the generalized additive (mixed) models with ordered-factor-smooth interaction smoothers modeling the mean secondary growth of *P. tremula* L. x *P. tremuloides* trees during the PPE under constant long-day (LD) light, temperature, and relative humidity conditions (i.e., 18:6 hours light/dark cycles; 20:18 °C warm/cold cycles; and 60% relative humidity, respectively). The mean secondary growth of the reference WT^{T89} is represented by the horizontal red dotted line. In contrast, the mean secondary growth of the independently RNAi transformed transgenic lines is represented by colored solid lines. The shaded bands around the smooth terms represent Bayesian Wabha/Silverman credible intervals. Doy is the abbreviation for day of the year. Doy counts started on the 1st of January 2020.

Field Experiment 1 (FE1)

Primary growth

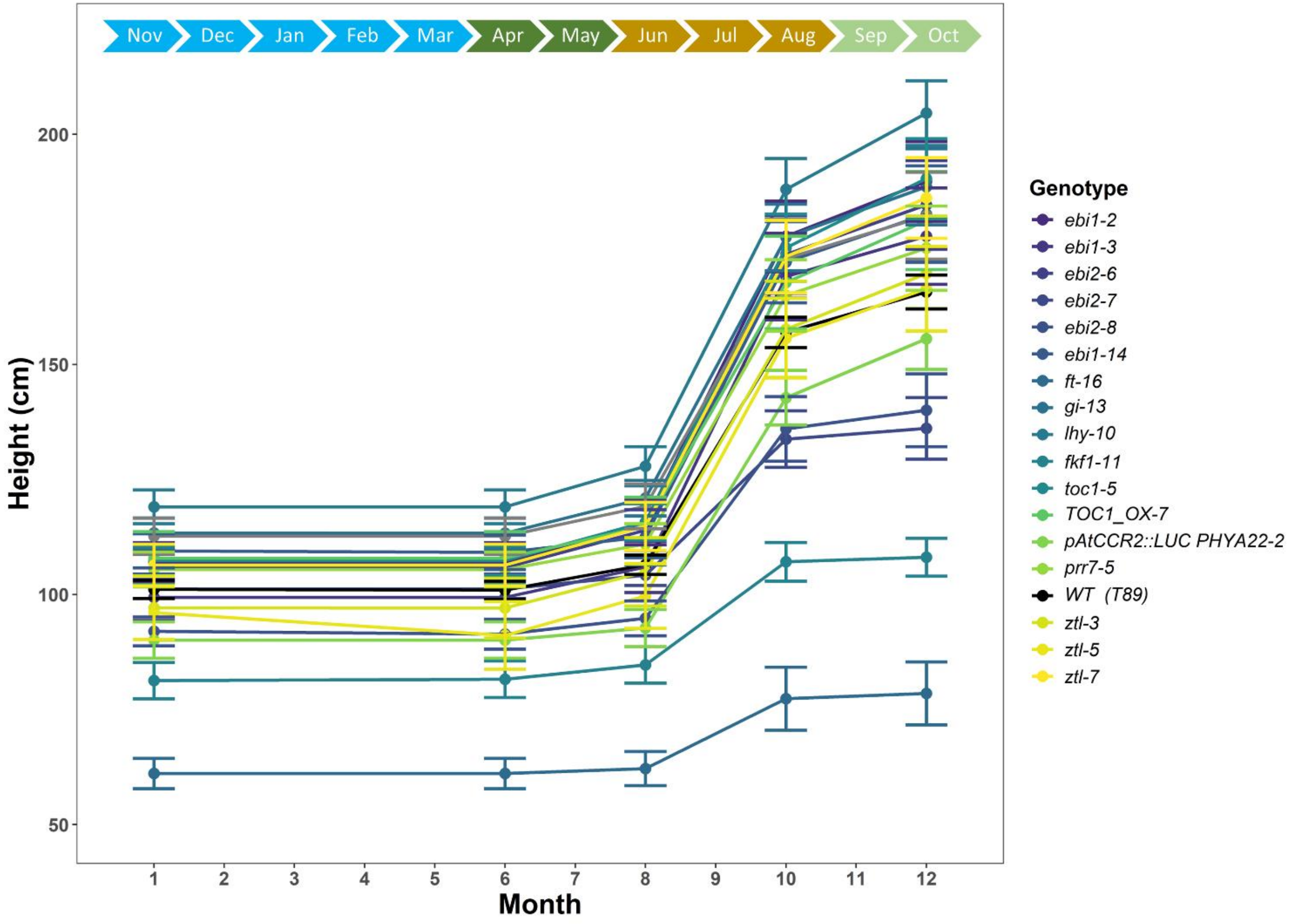

Figure S9. The primary growth of *P. tremula* L. x *P. tremuloides* trees during FE1. The black solid line represents the primary growth of the reference WT^{T89} while the colored solid lines represent the primary growth of independently RNAi transformed transgenic lines. Dots and error bars represent mean and standard error values, respectively. Month counts started in November 2014.

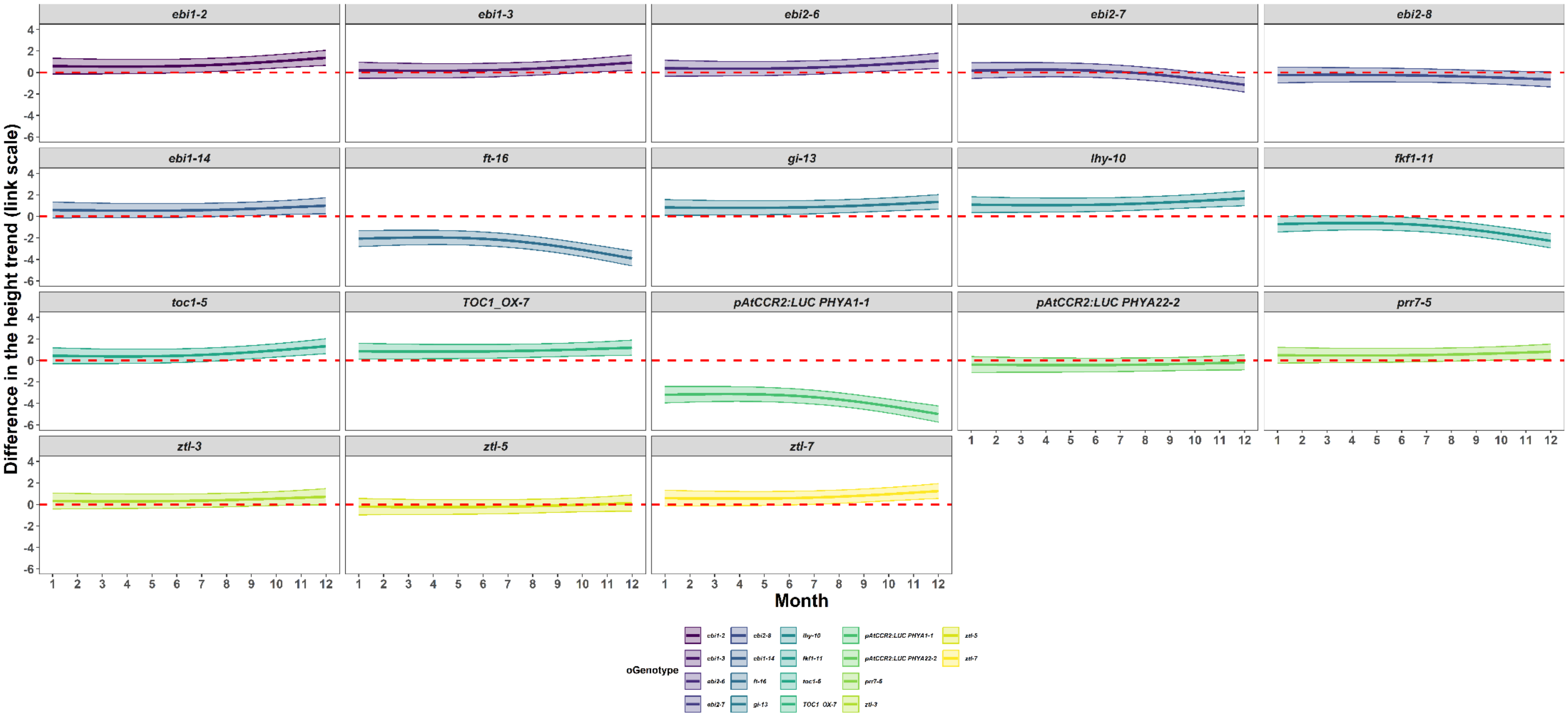

Figure S10. Term plots of the generalized additive (mixed) models with ordered-factor-smooth interaction smoothers modeling the mean primary growth of *P. tremula* L. x *P. tremuloides* trees during FE1. The mean primary growth of the reference WT^{T89} is represented by the horizontal red dotted line. In contrast, the mean primary growth of the independently RNAi transformed transgenic lines is represented by colored solid lines. The shaded bands around the smooth terms represent Bayesian Wabha/Silverman credible intervals. Month counts started in November 2014.

Secondary growth

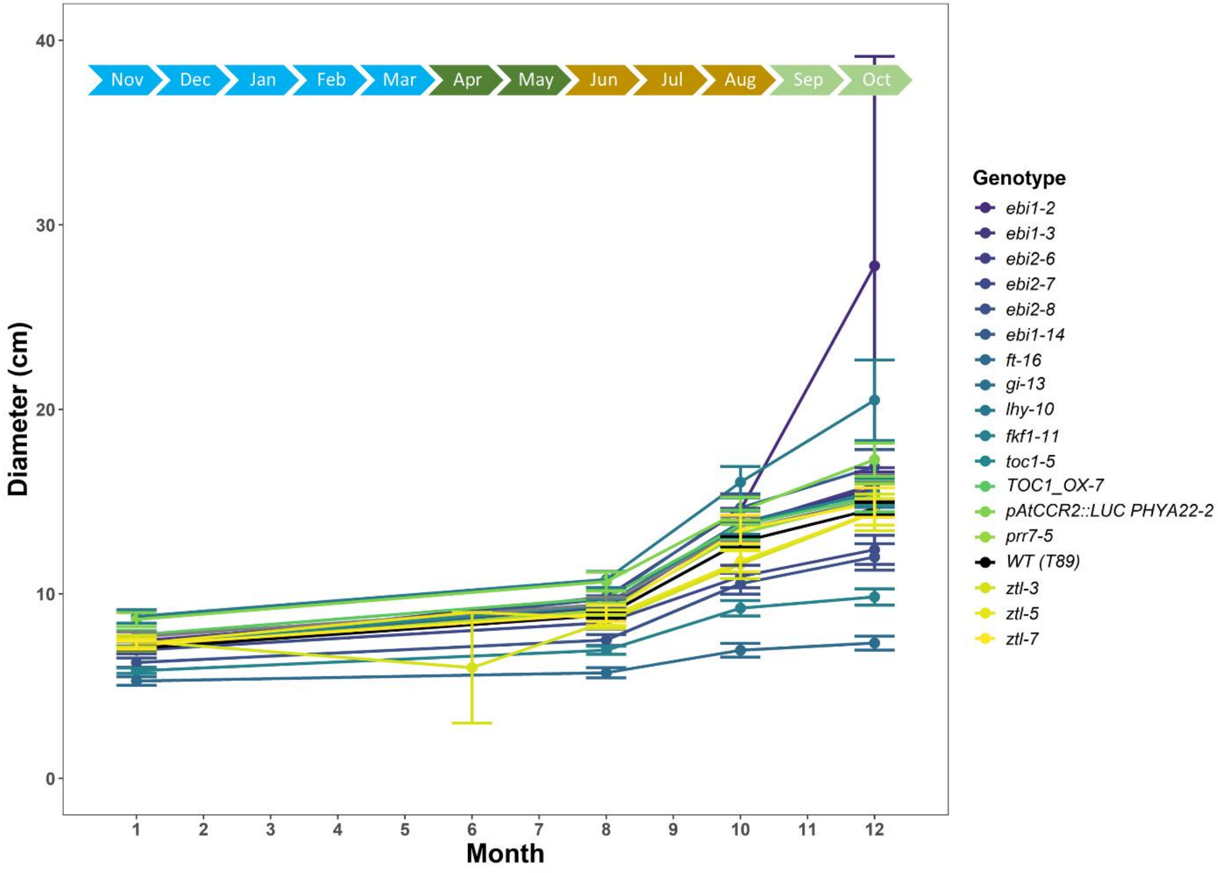

Figure S11. The secondary growth of *P. tremula* L. x *P. tremuloides* trees during FE1. The black solid line represents the secondary growth of the reference WT^{T89} while the colored solid lines represent the secondary growth of independently RNAi transformed transgenic lines. Dots and error bars represent mean and standard error values, respectively. Month counts started in November 2014.

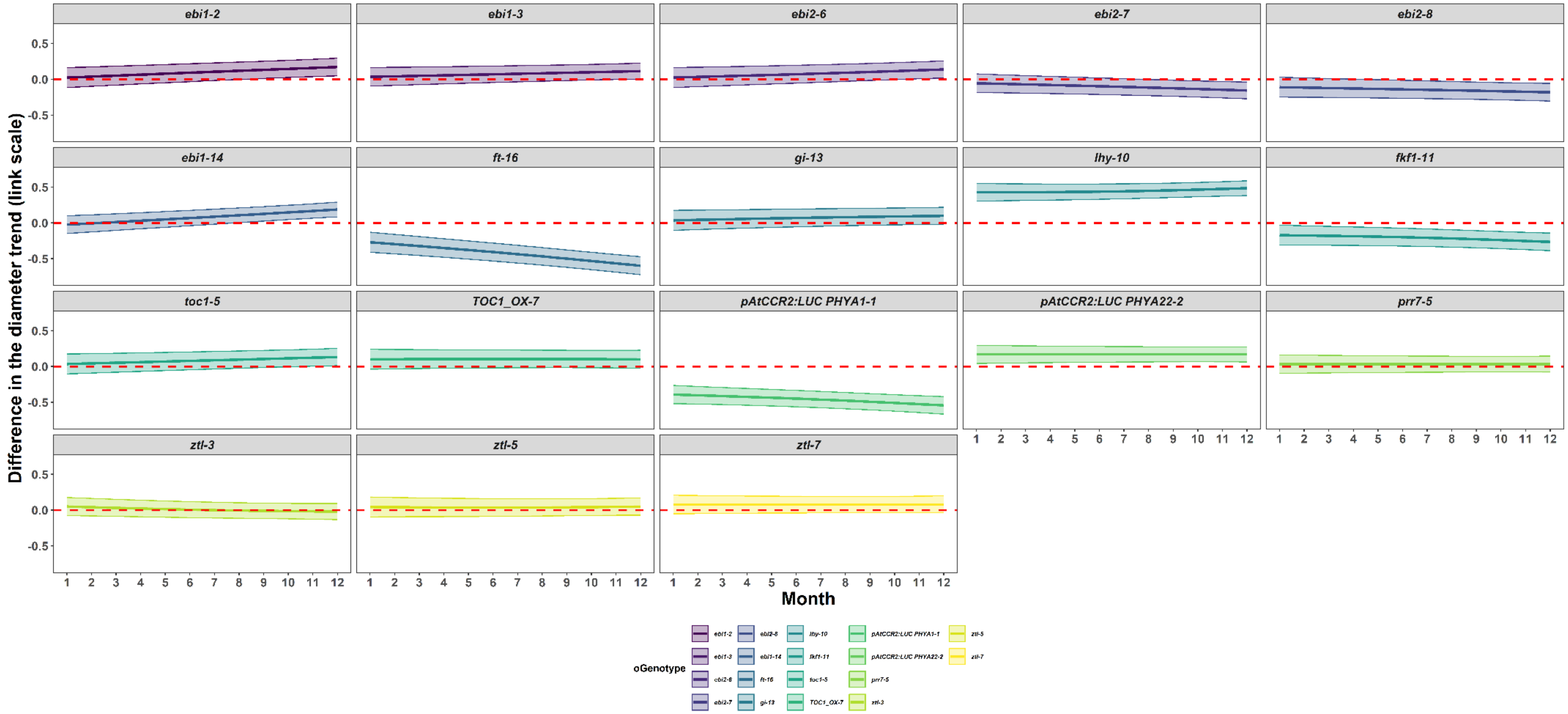

Figure S12. Term plots of the generalized additive (mixed) models with ordered-factor-smooth interaction smoothers modeling the mean secondary growth of *P. tremula* L. x *P. tremuloides* trees during FE1. The mean secondary growth of the reference WT^{T89} is represented by the horizontal red dotted line. In contrast, the mean secondary growth of the independently RNAi transformed transgenic lines is represented by colored solid lines. The shaded bands around the smooth terms represent Bayesian Wabha/Silverman credible intervals. Month counts started in November 2014.

Chlorophyll content index

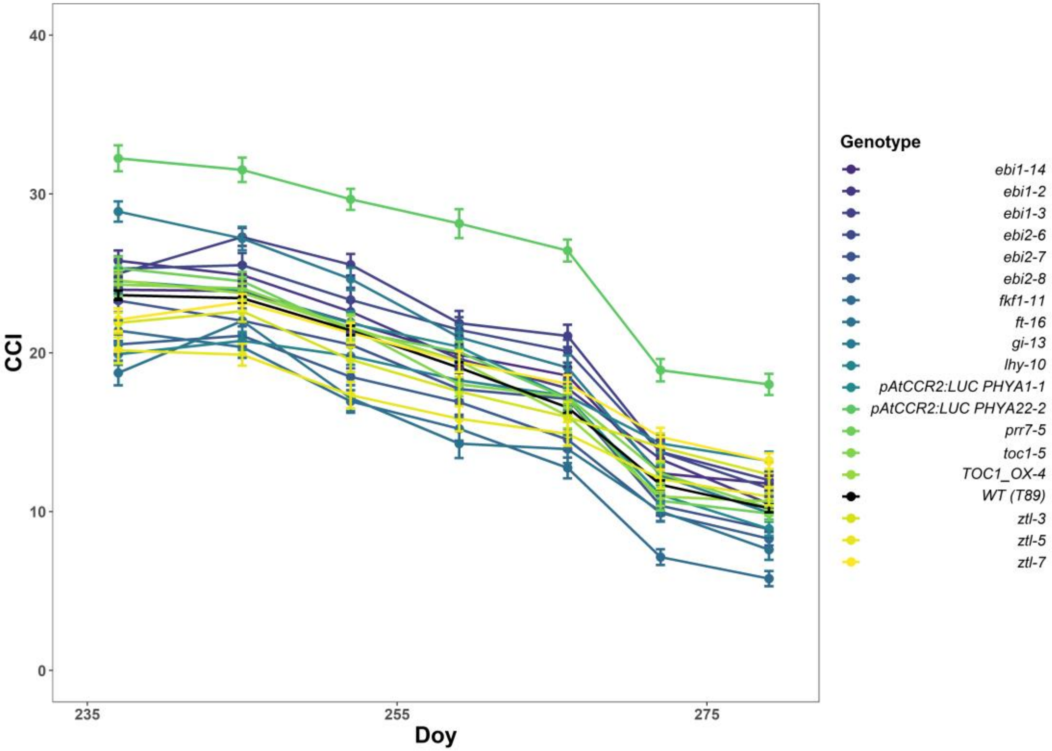

Figure S13. The chlorophyll content index (CCI) development in *P. tremula* L. x *P. tremuloides* trees during FE1. The black solid line represents the CCI of the reference WT^{T89} while the colored solid lines represent the CCI of independently RNAi transformed transgenic lines. Dots and error bars represent mean and standard error values, respectively. Doy is the abbreviation for day of the year. Doy counts started on the 1st of January 2014.

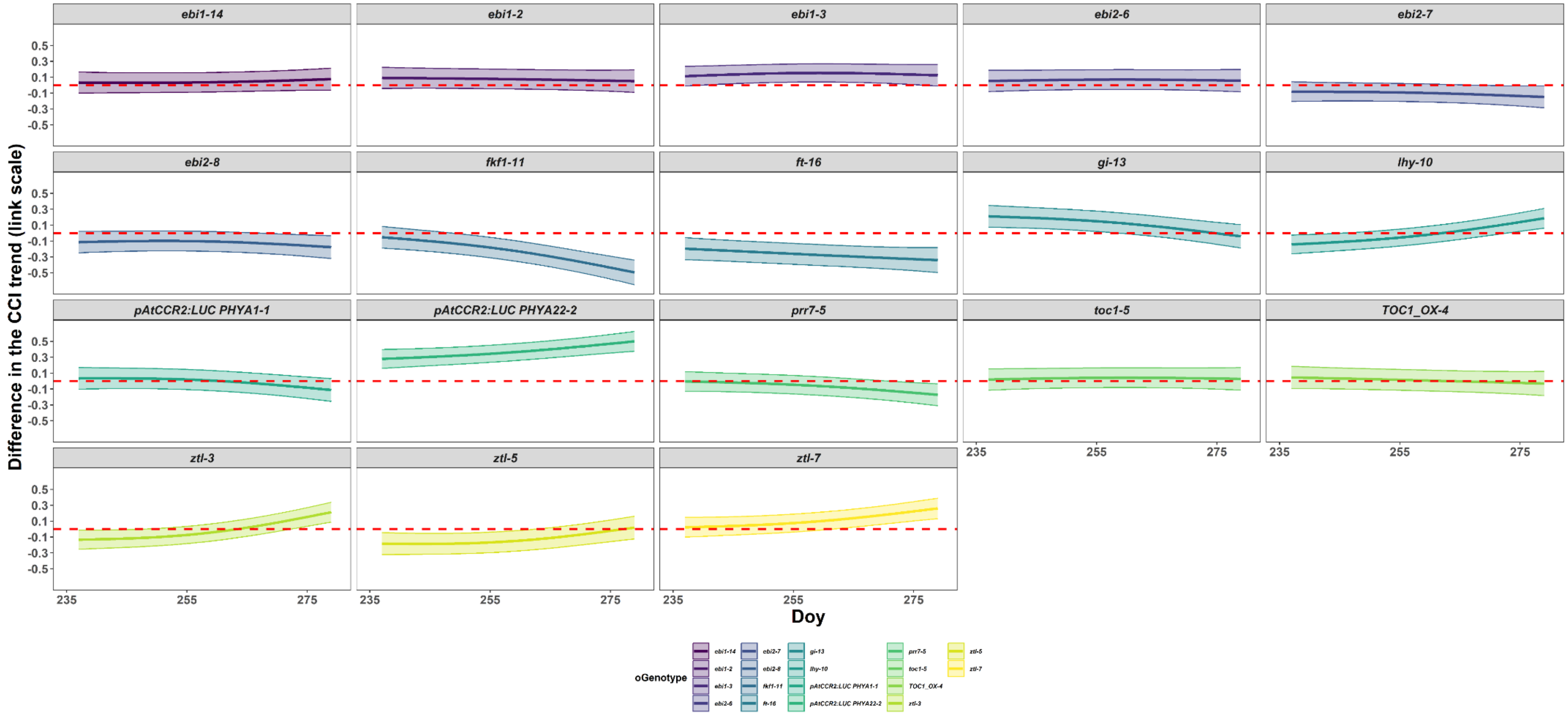

Figure S14. Term plots of the generalized additive (mixed) models with ordered-factor-smooth interaction smoothers modeling the chlorophyll content index (CCI) development of *P. tremula* L. x *P. tremuloides* trees during FE1. The mean CCI of the reference WT^{T89} is represented by the horizontal red dotted line. In contrast, the mean CCI of the independently RNAi transformed transgenic lines is represented by colored solid lines. The shaded bands around the smooth terms represent Bayesian Wabha/Silverman credible intervals. Doy is the abbreviation for day of the year. Doy counts started on the 1st of January 2014.

Bud set

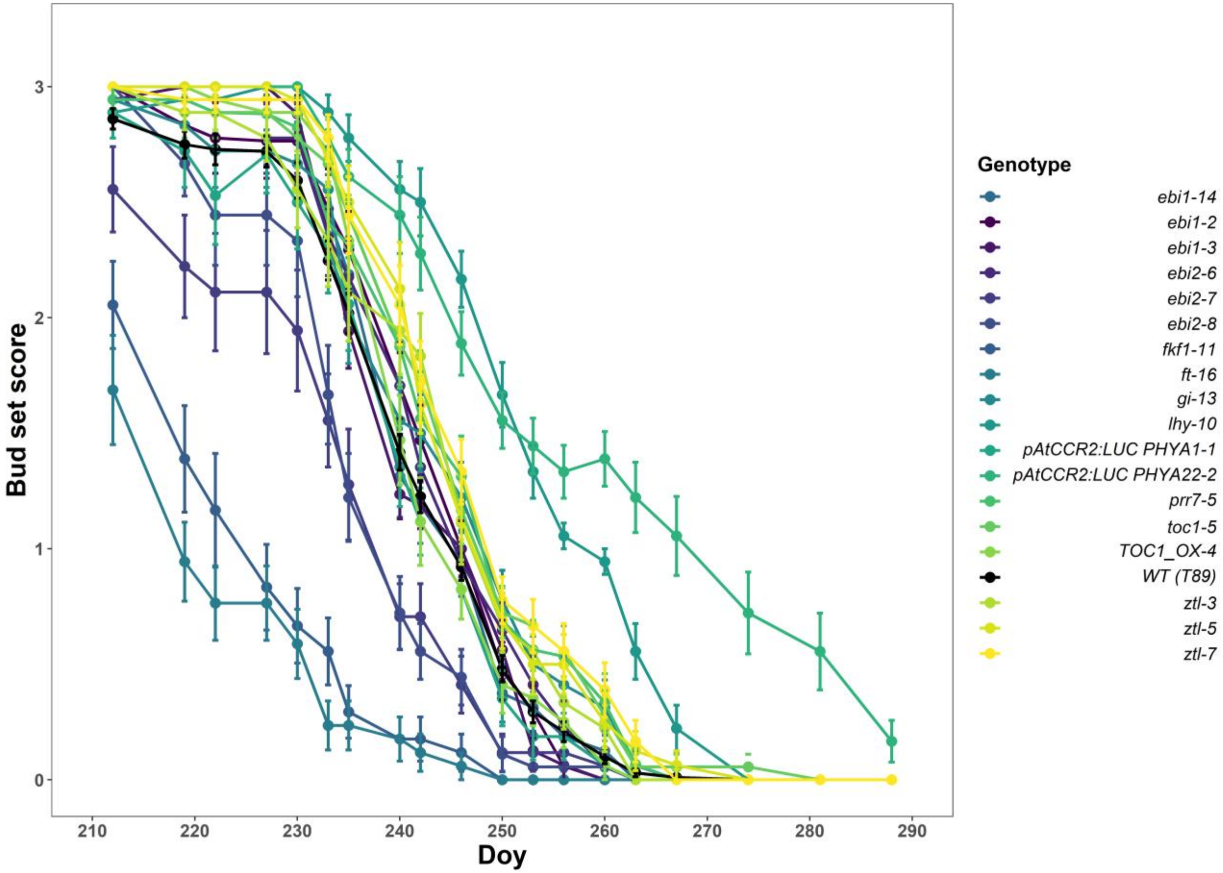

Figure S15. The bud set development in in *P. tremula* L. x *P. tremuloides* trees during FE1. The black solid line represents the bud set stage of the reference WT^{T89} while the colored solid lines represent the bud set stage of independently RNAi transformed transgenic lines. Dots and error bars represent mean and standard error values, respectively. The bud set was scored following UPOV [1] and Ibanez, et al. [2] using the following (opposite) scoring values: a still actively growing shoot and uninitiated bud set (3), initiation of the bud set and cessation of growth (2), formation of the buds (1), and completed bud set (0)[2-4]. Doy is the abbreviation for day of the year. Doy counts started on the 1st of January 2014.

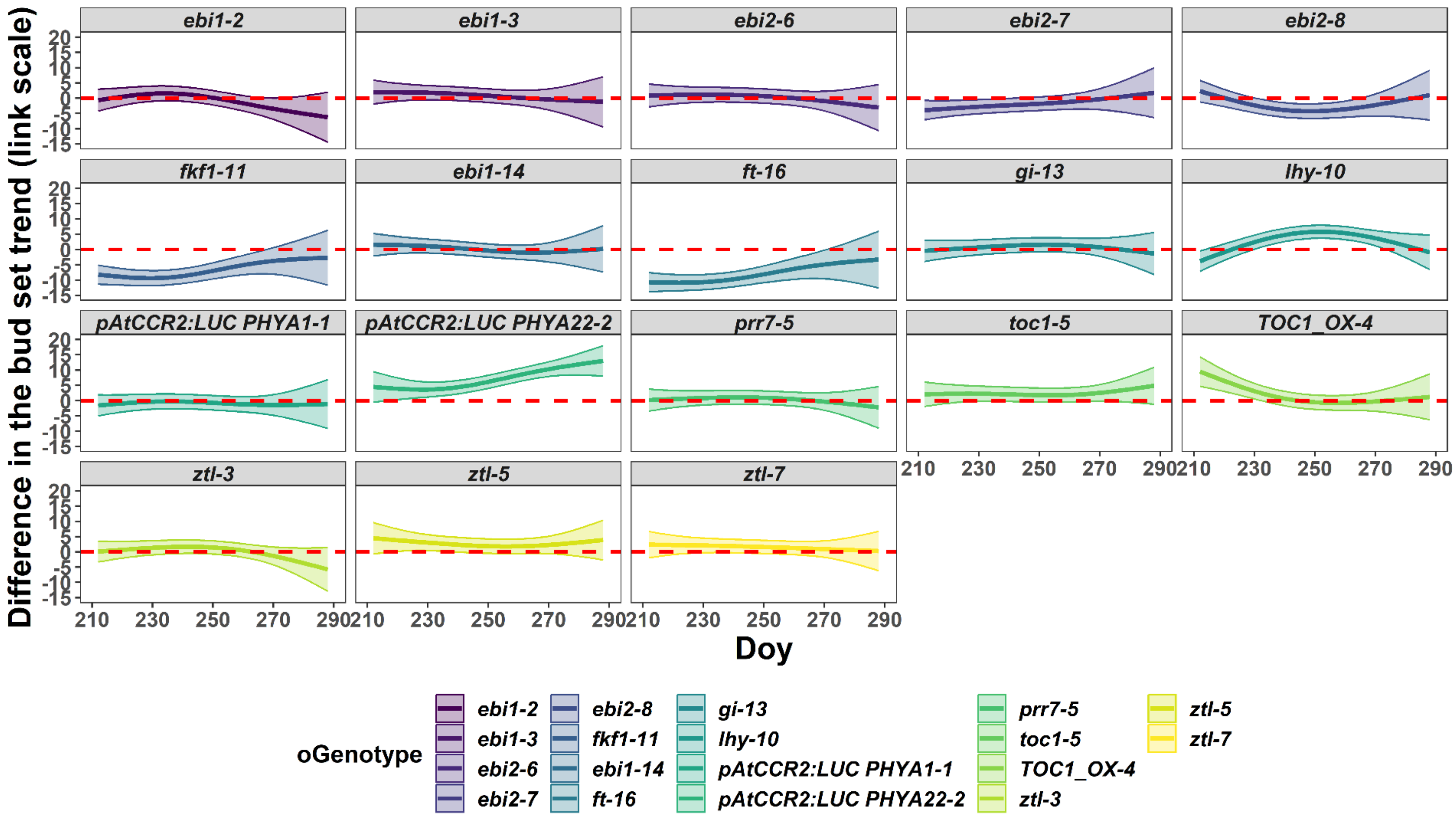

Figure S16. Term plots of the generalized additive (mixed) models with ordered-factor-smooth interaction smoothers modeling the bud set development of *P. tremula* L. x *P. tremuloides* trees during FE1. The mean bud set of the reference WT^{T89} is represented by the horizontal red dotted line. In contrast, the mean bud set of the independently RNAi transformed transgenic lines is represented by colored solid lines. The shaded bands around the smooth terms represent Bayesian Wabha/Silverman credible intervals. Doy is the abbreviation for day of the year. Doy counts started on the 1st of January 2014.

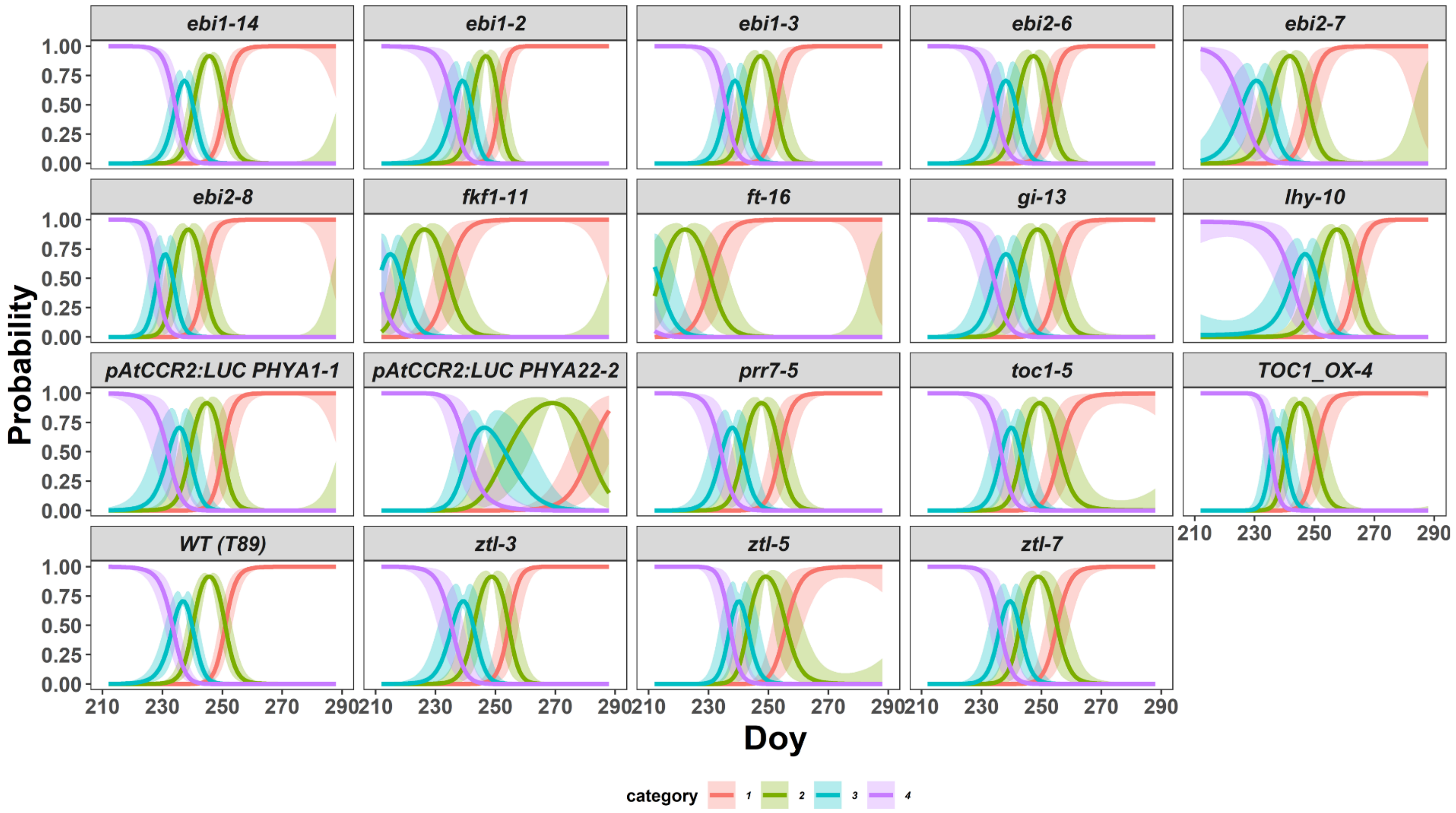

Figure S17. Term plots of the generalized additive (mixed) models with ordered-factor-smooth interaction smoothers modeling the bud set development of *P. tremula* L. x *P. tremuloides* trees during FE1. The colored solid lines, given for the reference WT^{T89} and the independently RNAi transformed transgenic lines, represent the predicted probability to have a bud in a certain bud set stage at a particular moment in time. The shaded bands around the colored solid lines represent 95% pointwise confidence intervals. The bud set was scored following UPOV [1] and Ibanez, et al. [2] using the following (opposite) scoring values: a still actively growing shoot and uninitiated bud set (4; purple), initiation of the bud set and cessation of growth (3; teal), formation of the buds (2; light-green), and completed bud set (1; red)[2-4]. Doy counts started on the 1st of January 2014.

Growth Chamber Experiment 2 (GCE2)

Primary growth

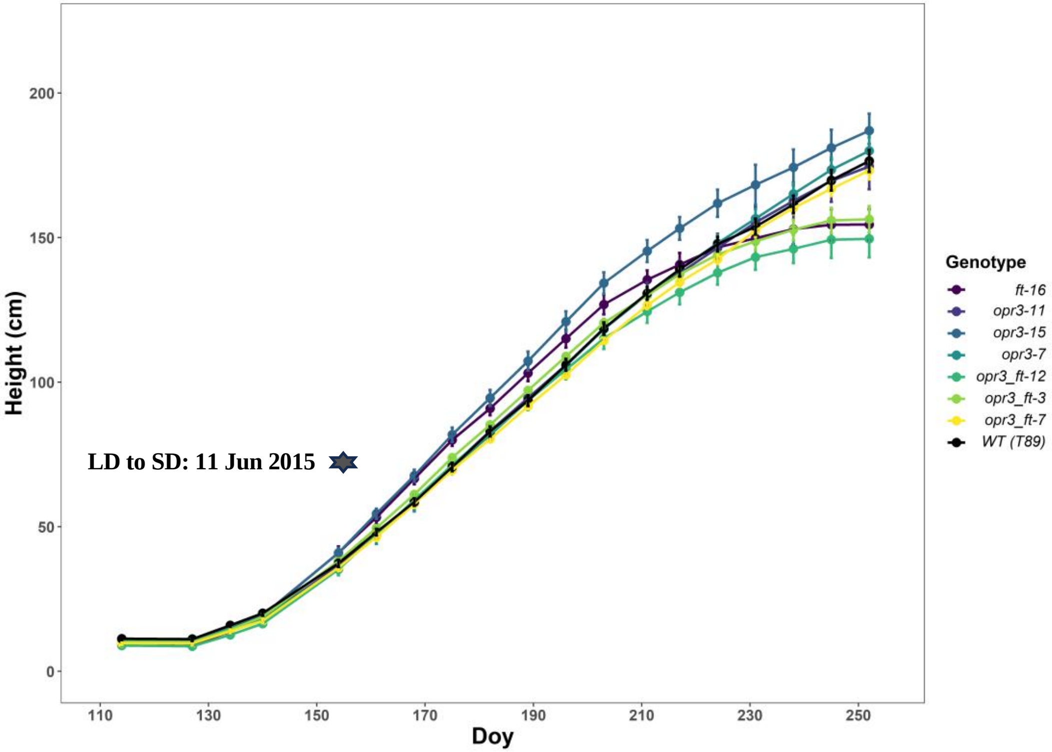

Figure S18. The primary growth of *P. tremula* L. x *P. tremuloides* trees during GCE2 under constant long-day (LD) light, temperature, and relative humidity conditions (i.e., 18:6 hours light/dark cycles; 18 °C; and 80% relative humidity, respectively). Light conditions were changed to short-day (SD) conditions (i.e., 16:8 hours light/dark cycles) on the 11th of June 2015 (Doy = 162). The black solid line represents the primary growth of the reference WT^{T89} while the colored solid lines represent the primary growth of independently RNAi transformed transgenic lines. Dots and error bars represent mean and standard error values, respectively. Doy is the abbreviation for day of the year. Doy counts started on the 1st of January 2015.

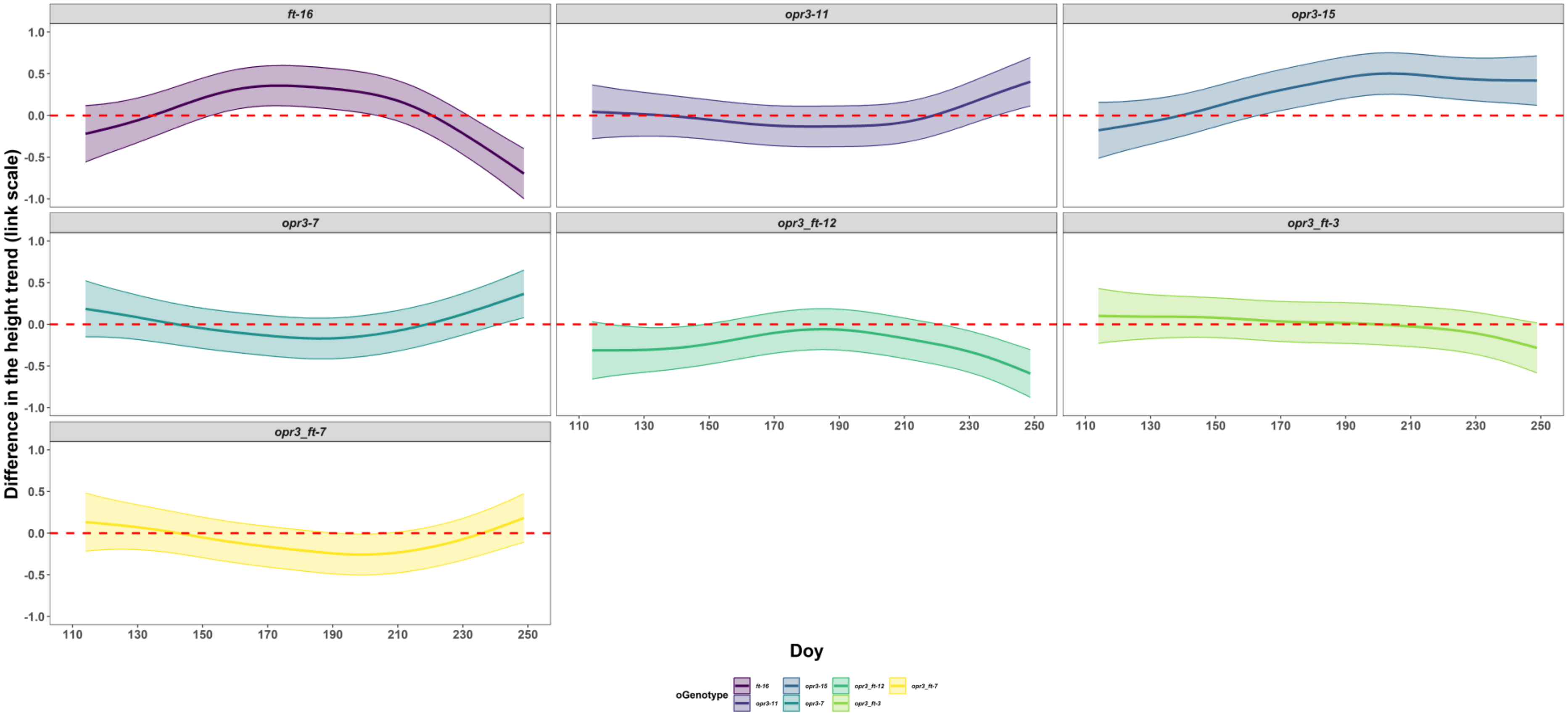

Figure S19. Term plots of the generalized additive (mixed) models with ordered-factor-smooth interaction smoothers modeling the mean primary growth of *P. tremula* L. x *P. tremuloides* trees during GCE2 under constant long-day (LD) light, temperature, and relative humidity conditions (i.e., 18:6 hours light/dark cycles; 18 °C; and 80% relative humidity, respectively). Light conditions were changed to short-day SD conditions (i.e., 16:8 hours light/dark cycles) on the 11th of June 2015 (Doy = 162). The mean primary growth of the reference WT^{T89} is represented by the horizontal red dotted line. In contrast, the mean primary growth of the independently RNAi transformed transgenic lines is represented by colored solid lines. The shaded bands around the smooth terms represent Bayesian Wabha/Silverman credible intervals. Doy is the abbreviation for day of the year. Doy counts started on the 1st of January 2015.

Growth Chamber Experiment 3 (GCE3)

Primary growth

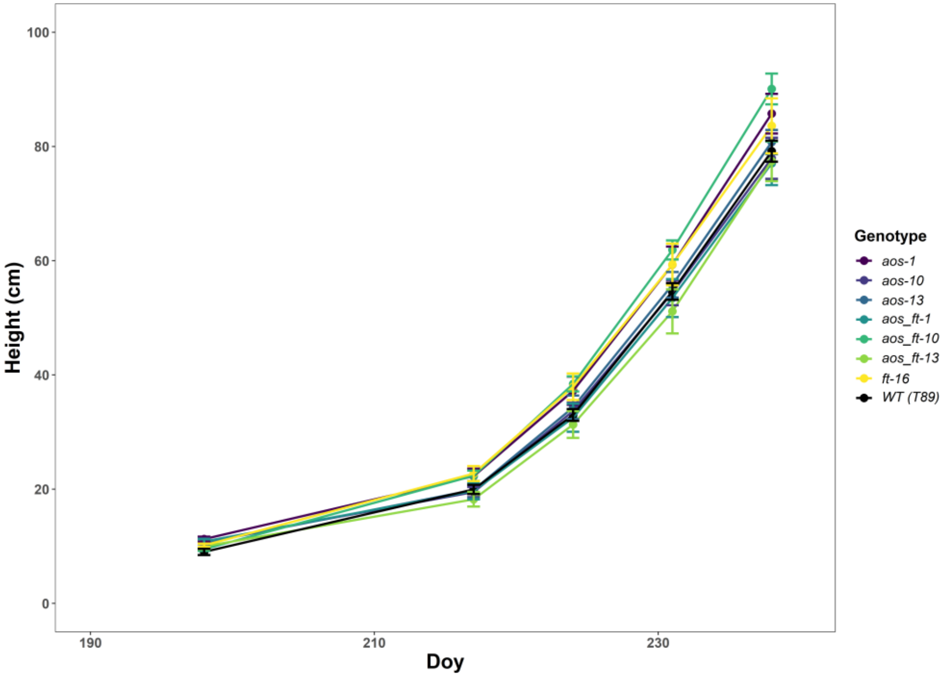

Figure S20. The primary growth of *P. tremula* L. x *P. tremuloides* trees during GCE3 under constant long-day (LD) light, temperature, and relative humidity conditions (i.e., 18:6 hours light/dark cycles; 18 °C; and 80% relative humidity, respectively). The black solid line represents the primary growth of the reference WT^{T89} while the colored solid lines represent the primary growth of independently RNAi transformed transgenic lines. Dots and error bars represent mean and standard error values, respectively. Doy is the abbreviation for day of the year. Doy counts started on the 1st of January 2015.

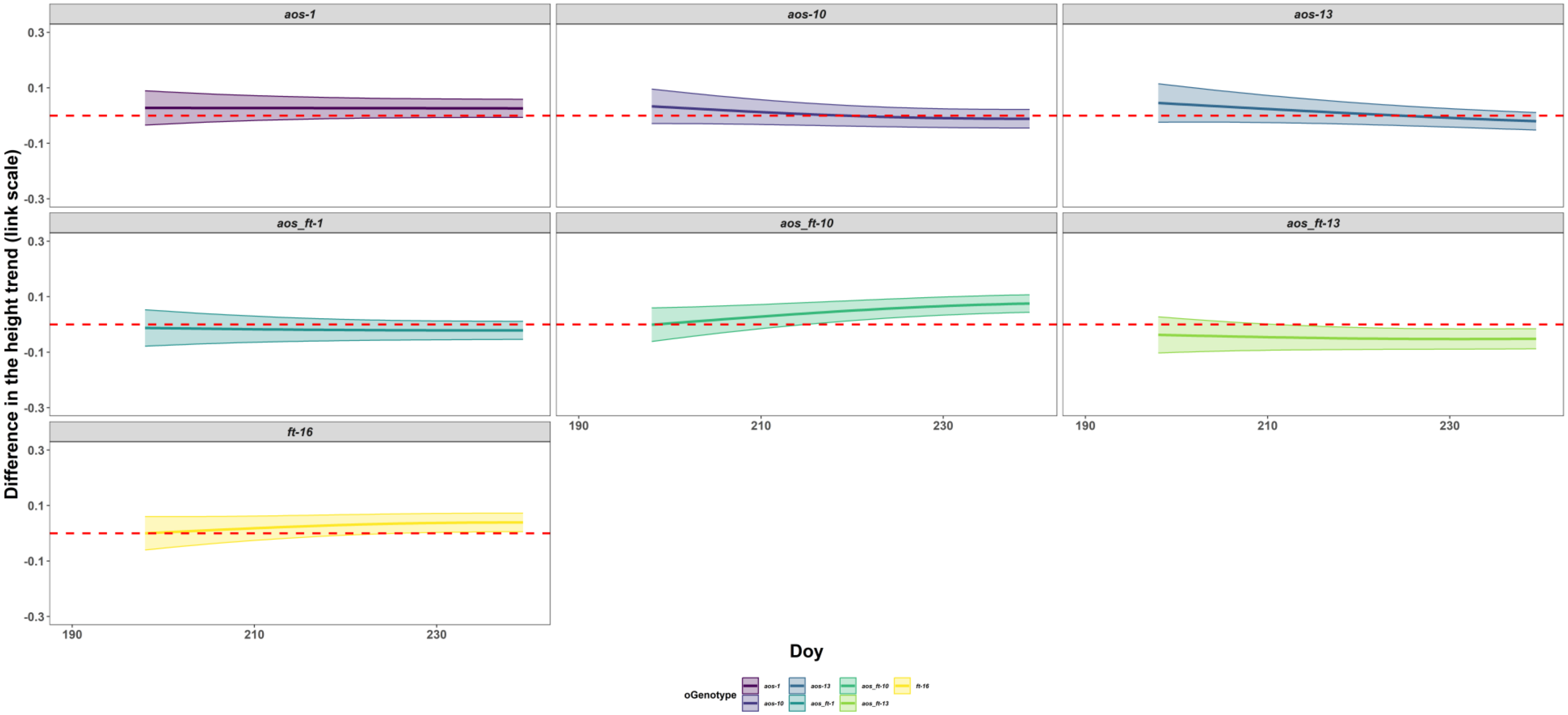

Figure S21. Term plots of the generalized additive (mixed) models with ordered-factor-smooth interaction smoothers modeling the mean primary growth of *P. tremula* L. x *P. tremuloides* trees during GCE3 under constant long-day (LD) light, temperature, and relative humidity conditions (i.e., 18:6 hours light/dark cycles; 18 °C; and 80% relative humidity, respectively). The mean primary growth of the reference WT^{T89} is represented by the horizontal red dotted line. In contrast, the mean primary growth of the independently RNAi transformed transgenic lines is represented by colored solid lines. The shaded bands around the smooth terms represent Bayesian Wabha/Silverman credible intervals. Doy is the abbreviation for day of the year. Doy counts started on the 1st of January 2015.

Bud set

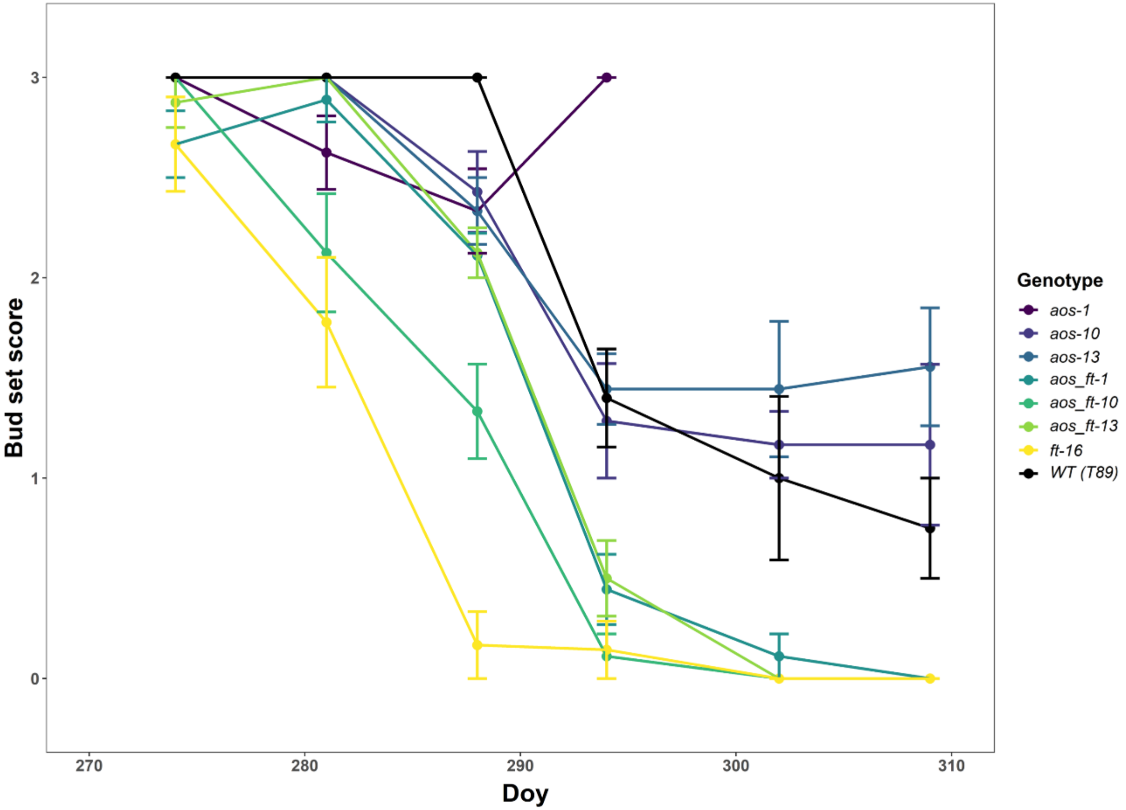

Figure S22. The bud set development in in *P. tremula* L. x *P. tremuloides* trees during GCE3 under constant short-day (SD) light, temperature, and relative humidity conditions (i.e., 15:9 hours light/dark cycles; 18°C; and 80% relative humidity, respectively). The black solid line represents the bud set stage of the reference WT^{T89} while the colored solid lines represent the bud set stage of independently RNAi transformed transgenic lines. Dots and error bars represent mean and standard error values, respectively. The bud set was scored following UPOV [1] and Ibanez, et al. [2] using the following (opposite) scoring values: a still actively growing shoot and uninitiated bud set (3), initiation of the bud set and cessation of growth (2), formation of the buds (1), and completed bud set (0)[2-4]. Doy is the abbreviation for day of the year. Doy counts started on the 1st of January 2015.

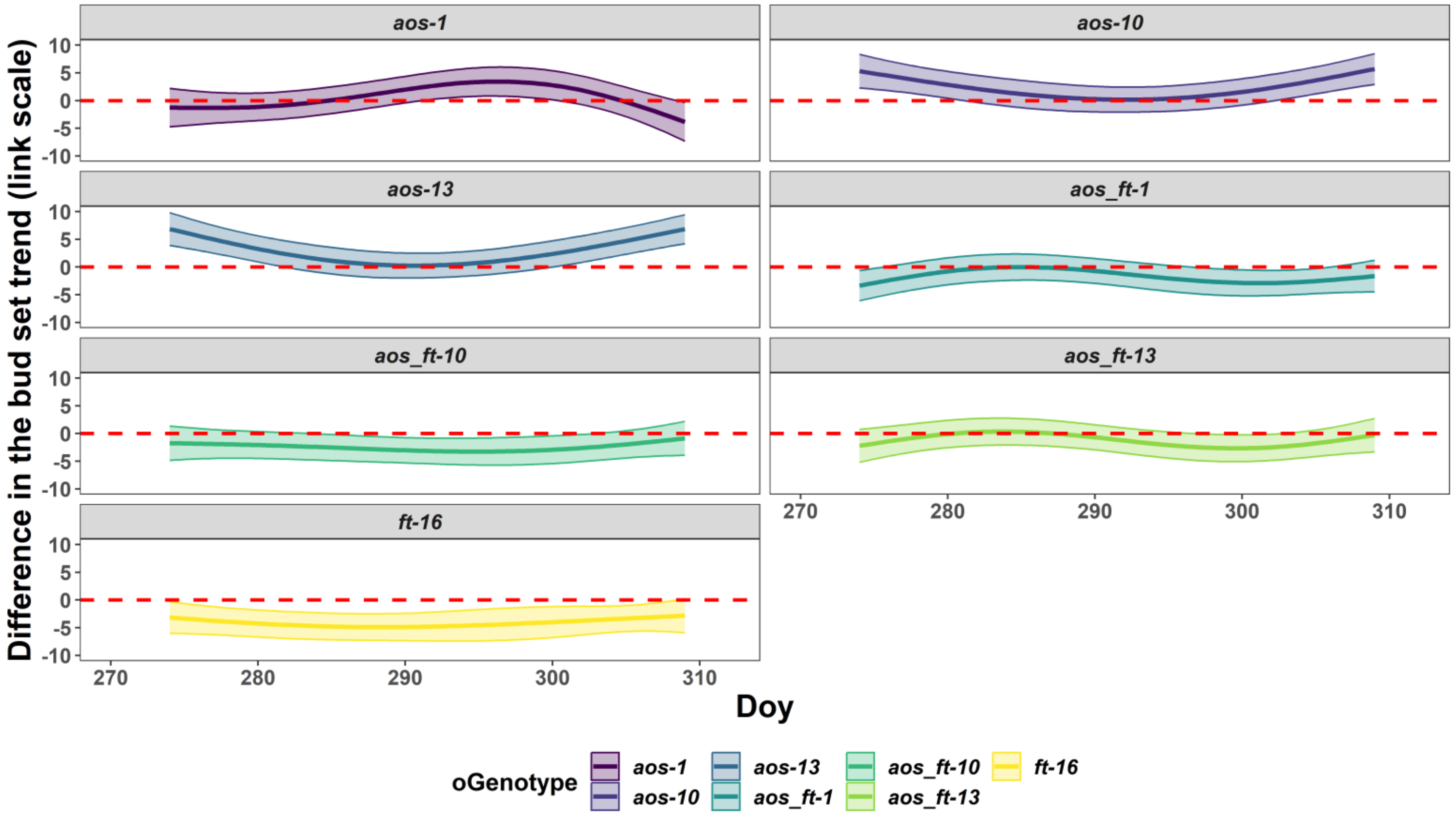

Figure S23. Term plots of the generalized additive (mixed) models with ordered-factor-smooth interaction smoothers modeling the bud set development of *P. tremula* L. x *P. tremuloides* trees during GCE3 under constant short-day (SD) light, temperature, and relative humidity conditions (i.e., 15:9 hours light/dark cycles; 18°C; and 80% relative humidity, respectively). The mean bud set of the reference WT^{T89} is represented by the horizontal red dotted line. In contrast, the mean bud set of the independently RNAi transformed transgenic lines is represented by colored solid lines. The shaded bands around the smooth terms represent Bayesian Wabha/Silverman credible intervals. Doy is the abbreviation for day of the year. Doy counts started on the 1st of January 2015.

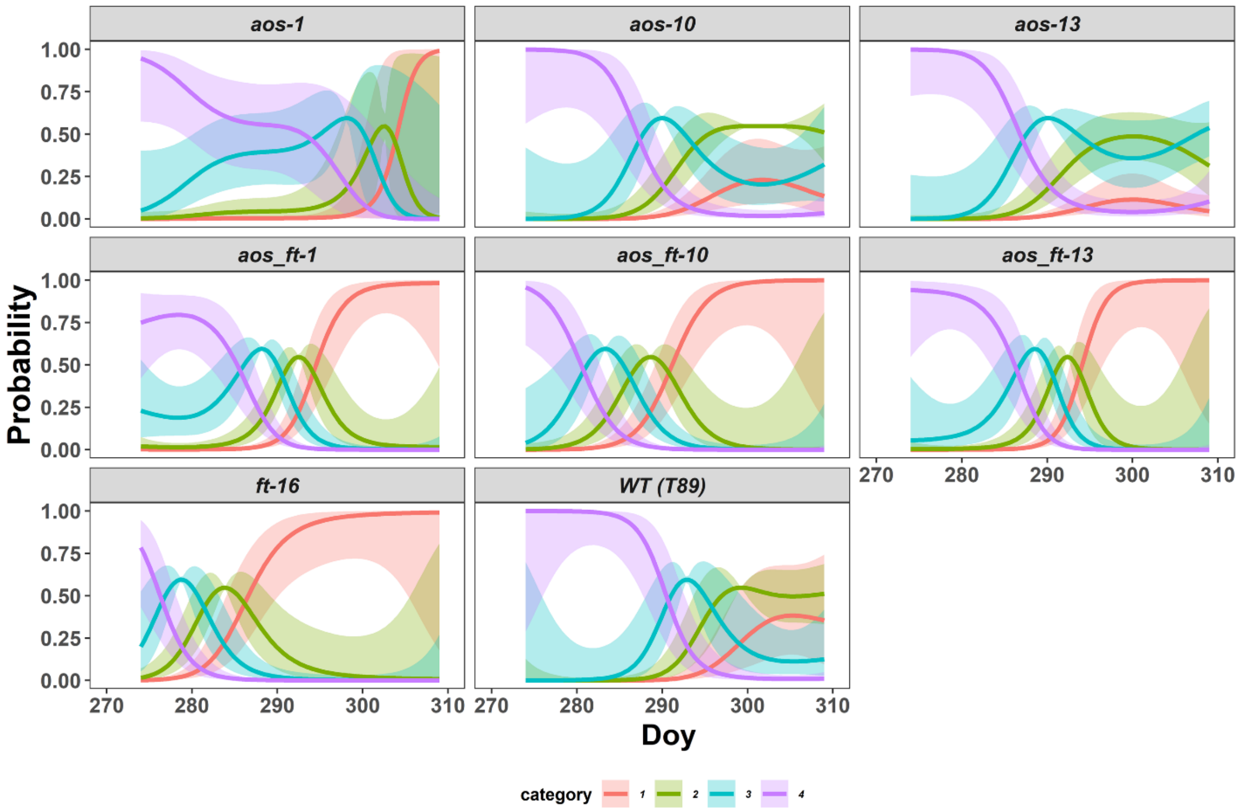

Figure S24. Term plots of the generalized additive (mixed) models with ordered-factor-smooth interaction smoothers modeling the bud set development of *P. tremula* L. x *P. tremuloides* trees during GCE3 under constant short-day (SD) light, temperature, and relative humidity conditions (i.e., 15:9 hours light/dark cycles; 18°C; and 80% relative humidity, respectively). The colored solid lines, given for the reference WT^{T89} and the independently RNAi transformed transgenic lines, represent the predicted probability to have a bud in a certain bud set stage at a particular moment in time. The shaded bands around the colored solid lines represent 95% pointwise confidence intervals. The bud set was scored following UPOV [1] and Ibanez, et al. [2] using the following (opposite) scoring values: a still actively growing shoot and uninitiated bud set (4; purple), initiation of the bud set and cessation of growth (3; teal), formation of the buds (2; light-green), and completed bud set (1; red)[2-4]. Doy is the abbreviation for day of the year. Doy counts started on the 1st of January 2015.

Apical bud burst

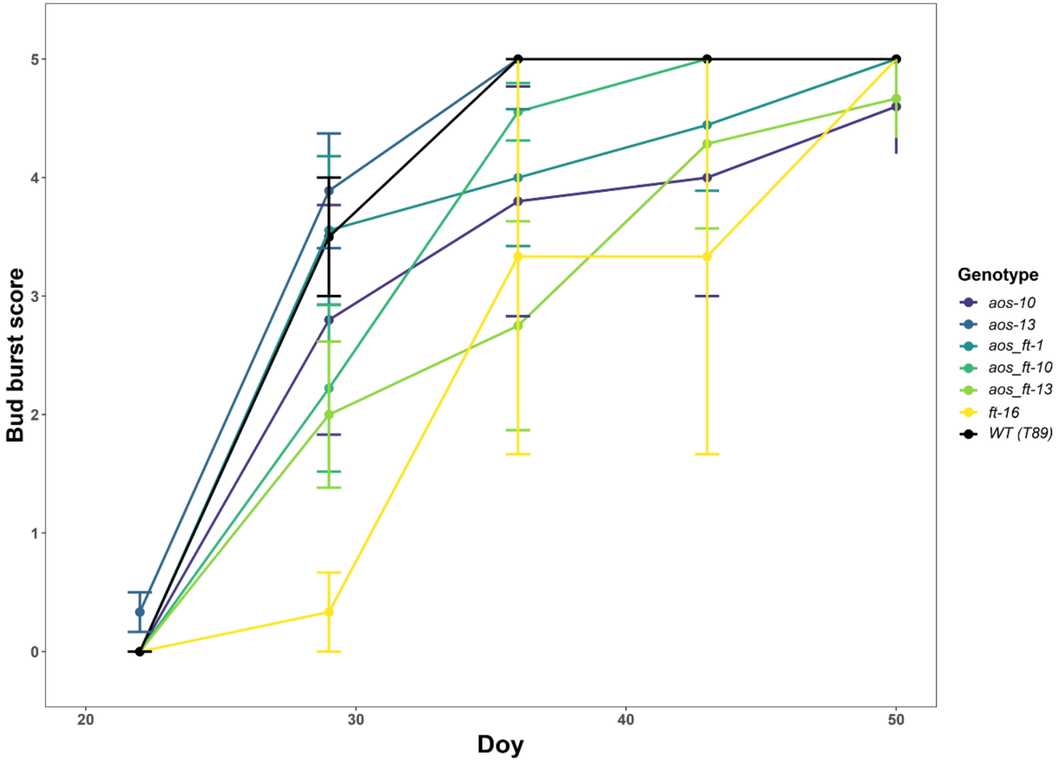

Figure S25. The apical bud burst development in in *P. tremula* L. x *P. tremuloides* trees during GCE3 under constant long-day (LD) light, temperature, and relative humidity conditions (i.e., 18:6 hours light/dark cycles; 18°C; and 80% relative humidity, respectively). The black solid line represents the apical bud burst stage of the reference WT^{T89} while the colored solid lines represent the apical bud burst stage of independently RNAi transformed transgenic lines. Dots and error bars represent mean and standard error values, respectively. The apical bud burst was scored following UPOV [1] using the following scoring values: dormant buds enveloped by scales (0), swelling buds with diverging scales (1), sprouting buds (2), opened buds with leaves clustered (3), diverging leaves with rolled op blades (4), and completely unfolded leaves (5). Doy is the abbreviation for day of the year. Doy counts started on the 1st of January 2015. No means and standard errors could be computed for *aos-1*.

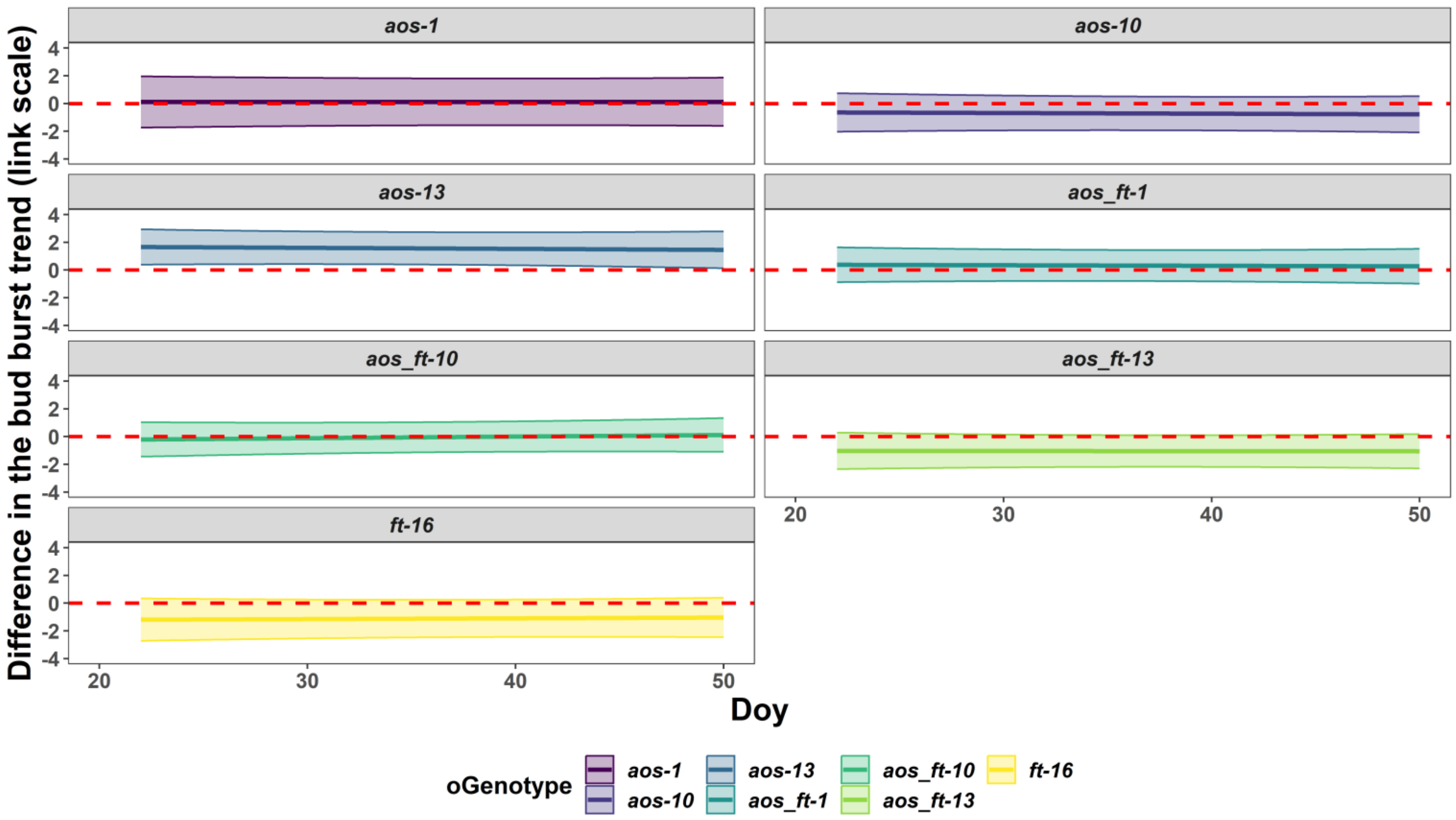

Figure S26. Term plots of the generalized additive (mixed) models with ordered-factor-smooth interaction smoothers modeling the apical bud burst development of *P. tremula* L. x *P. tremuloides* trees during GCE3 under constant long-day (LD) light, temperature, and relative humidity conditions (i.e., 18:6 hours light/dark cycles; 18°C; and 80% relative humidity, respectively). The mean apical bud burst of the reference WT^{T89} is represented by the horizontal red dotted line. In contrast, the mean apical bud burst of the independently RNAi transformed transgenic lines is represented by colored solid lines. The shaded bands around the smooth terms represent Bayesian Wabha/Silverman credible intervals. Doy is the abbreviation for day of the year. Doy counts started on the 1st of January 2015.

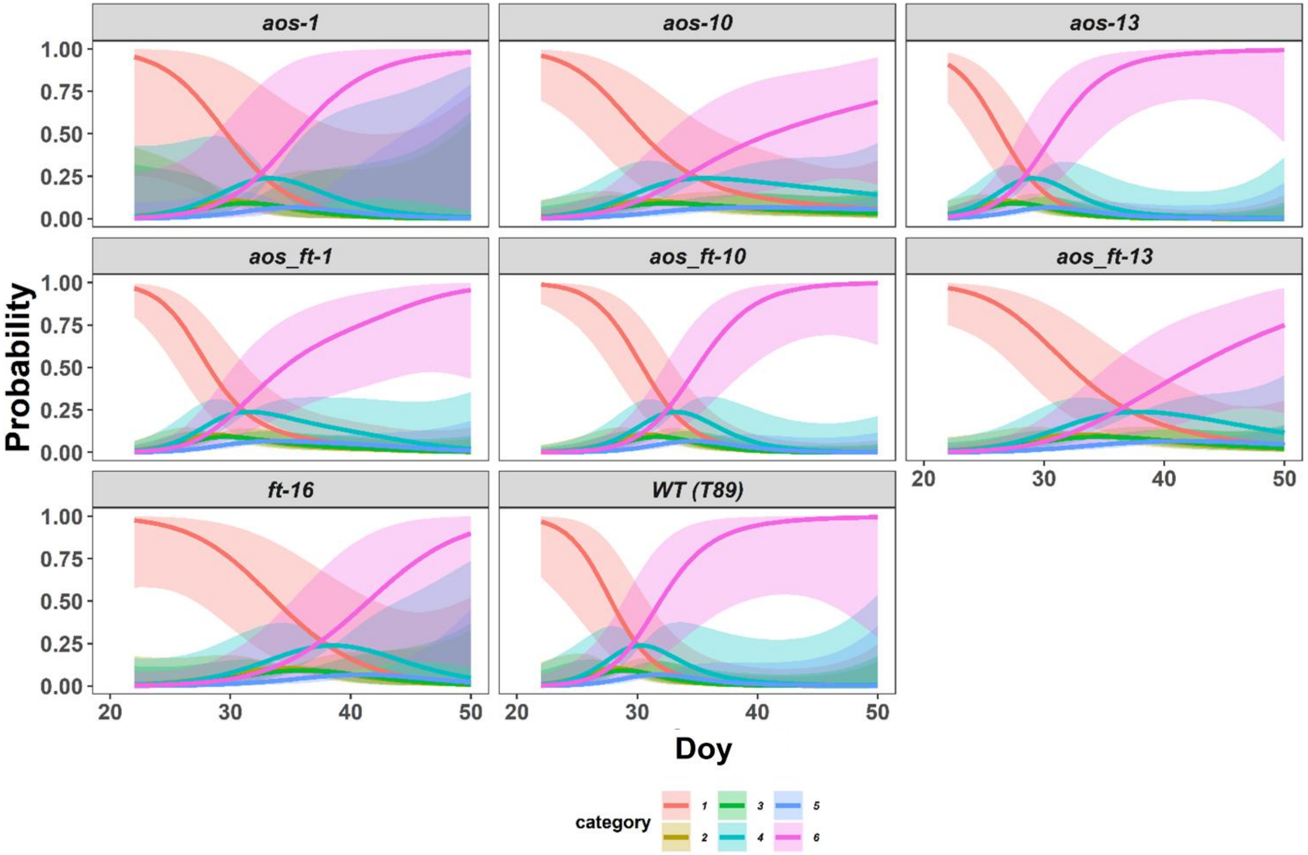

Figure S27. Term plots of the generalized additive (mixed) models with ordered-factor-smooth interaction smoothers modeling the apical bud burst development of *P. tremula* L. x *P. tremuloides* trees during GCE3 under constant long-day (LD) light, temperature, and relative humidity conditions (i.e., 18:6 hours light/dark cycles; 18 °C; and 80% relative humidity, respectively). The colored solid lines, given for the reference WT^{T89} and the independently RNAi transformed transgenic lines, represent the predicted probability to have a bud in a certain apical bud burst stage at a particular moment in time. The shaded bands around the colored solid lines represent 95% pointwise confidence intervals. The apical bud burst was scored following UPOV [1] using the following scoring values: dormant buds enveloped by scales (1; red), swelling buds with diverging scales (2; orange), sprouting buds (3; light-green), opened buds with leaves clustered (4; teal), diverging leaves with rolled op blades (5; blue), and completely unfolded leaves (6; purple). Doy is the abbreviation for day of the year. Doy counts started on the 1st of January 2015.

Lateral bud burst

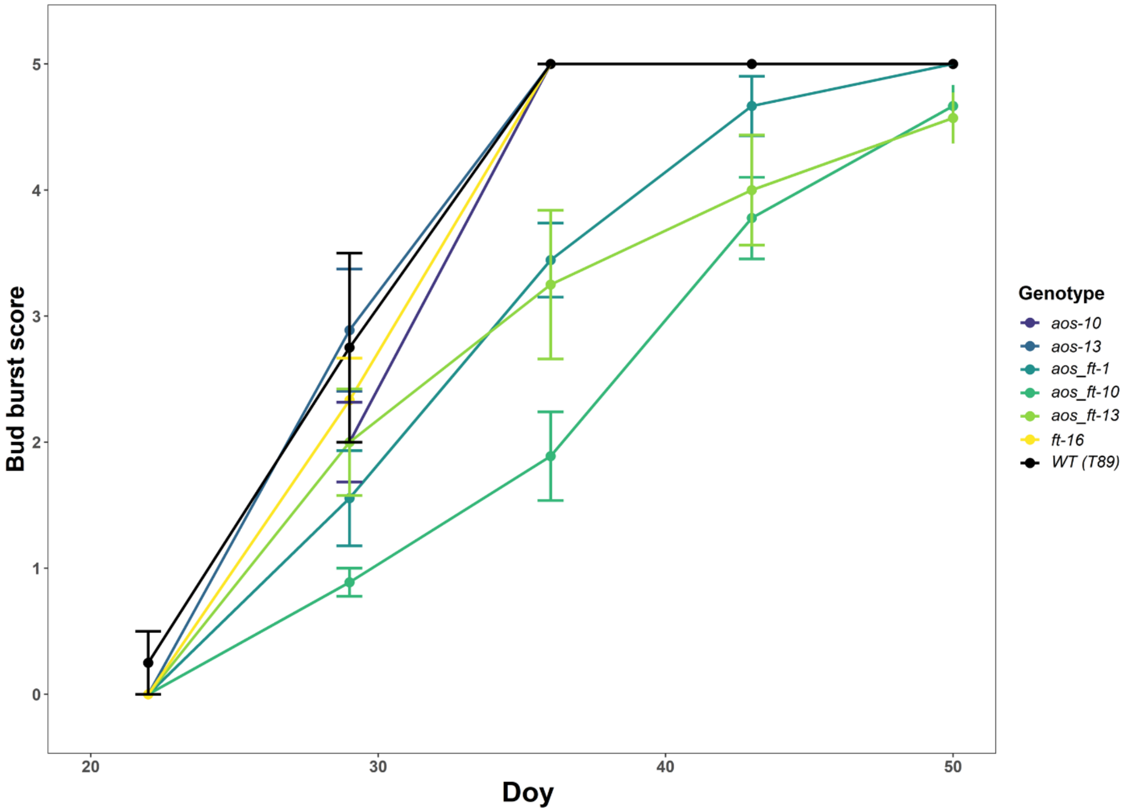

Figure S28. The lateral bud burst development in in *P. tremula* L. x *P. tremuloides* trees during GCE3 under constant long-day (LD) light, temperature, and relative humidity conditions (i.e., 18:6 hours light/dark cycles; 18 °C; and 80% relative humidity, respectively). The black solid line represents the lateral bud burst stage of the reference WT^{T89} while the colored solid lines represent the lateral bud burst stage of independently RNAi transformed transgenic lines. Dots and error bars represent mean and standard error values, respectively. The lateral bud burst was scored following UPOV [1] using the following scoring values: dormant buds enveloped by scales (0), swelling buds with diverging scales (1), sprouting buds (2), opened buds with leaves clustered (3), diverging leaves with rolled op blades (4), and completely unfolded leaves (5). Doy is the abbreviation for day of the year. Doy counts started on the 1st of January 2015. No means and standard errors could be computed for *aos-1*.

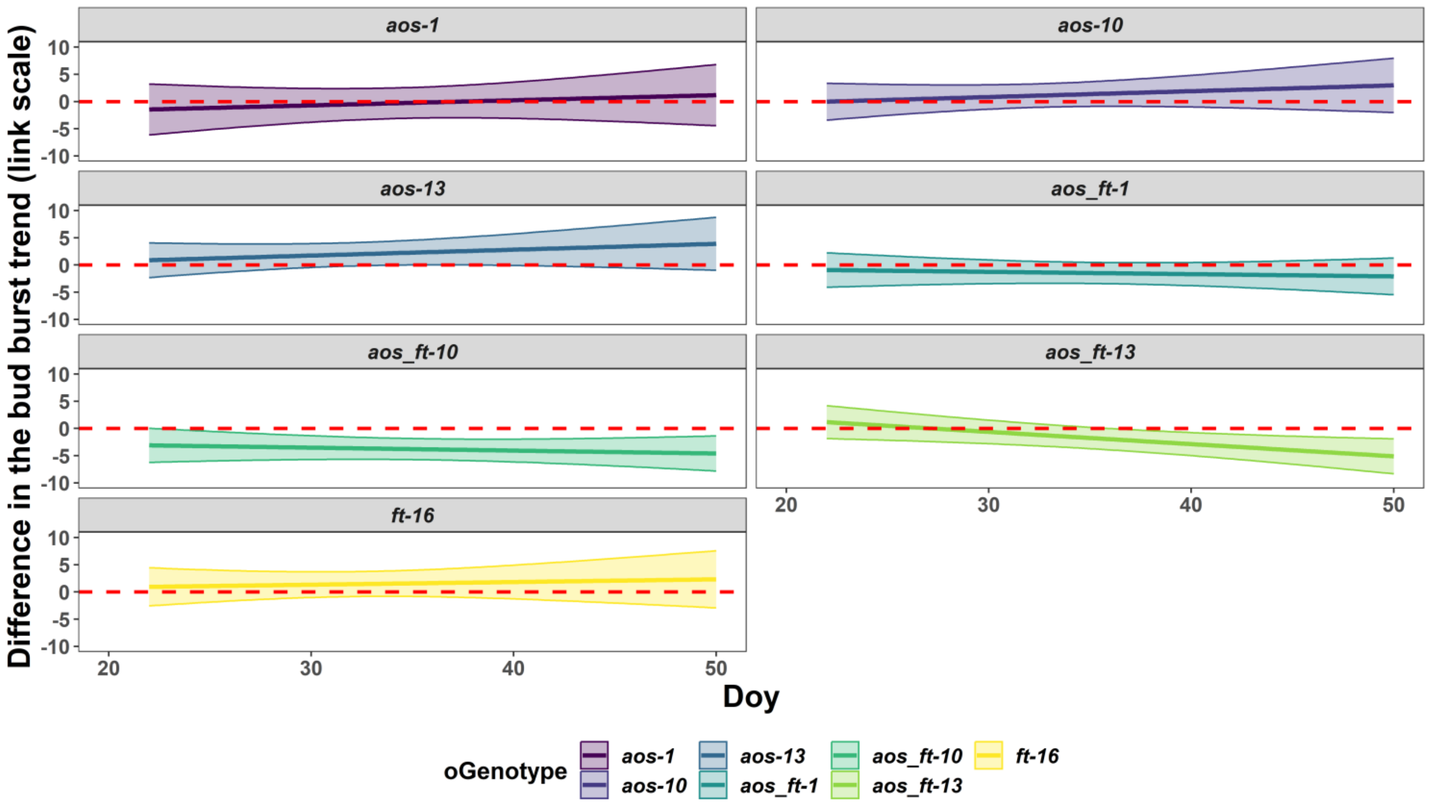

Figure S29. Term plots of the generalized additive (mixed) models with ordered-factor-smooth interaction smoothers modeling the lateral bud burst development of *P. tremula* L. x *P. tremuloides* trees during GCE3 under constant long-day (LD) light, temperature, and relative humidity conditions (i.e., 18:6 hours light/dark cycles; 18 °C; and 80% relative humidity, respectively). The mean lateral bud burst of the reference WT^{T89} is represented by the horizontal red dotted line. In contrast, the mean lateral bud burst of the independently RNAi transformed transgenic lines is represented by colored solid lines. The shaded bands around the smooth terms represent Bayesian Wabha/Silverman credible intervals. Doy is the abbreviation for day of the year. Doy counts started on the 1st of January 2015.

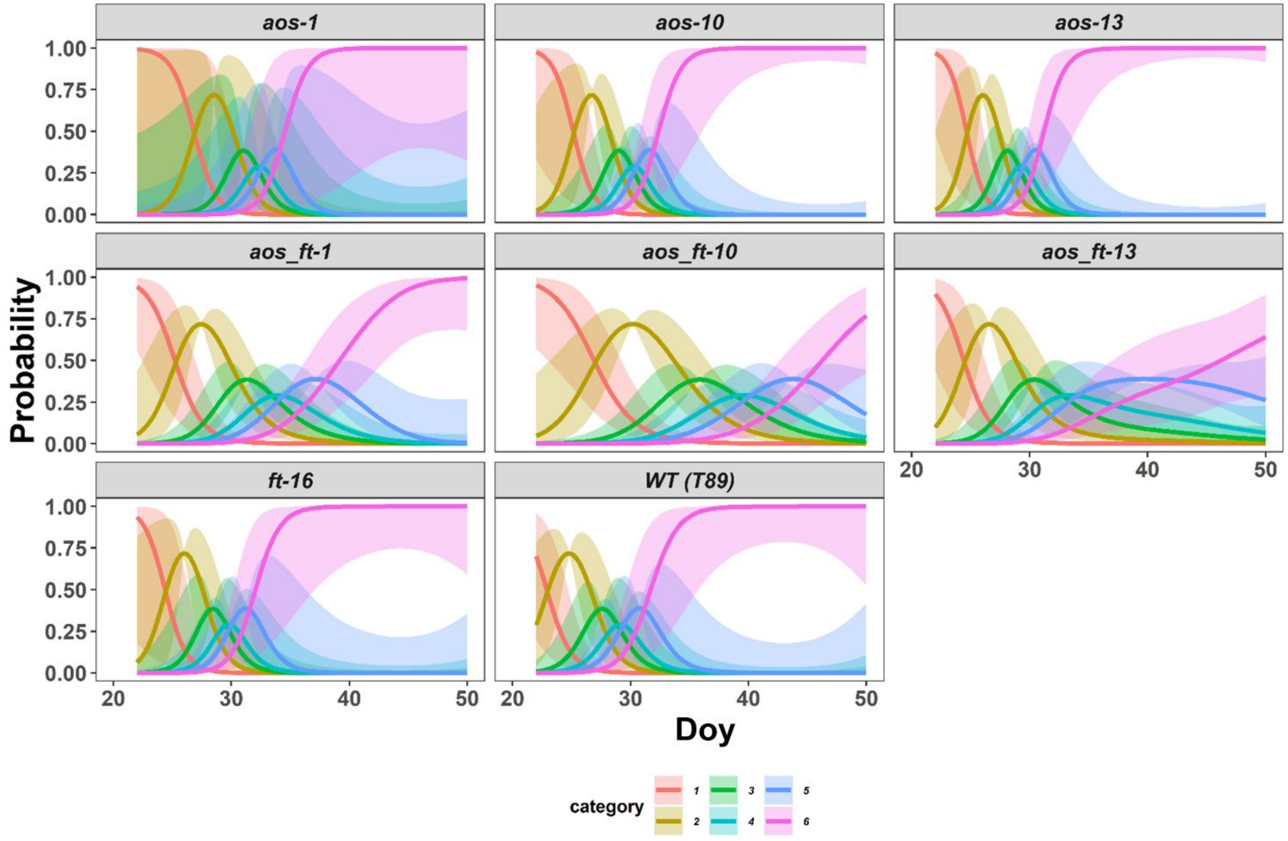

Figure S30. Term plots of the generalized additive (mixed) models with ordered-factor-smooth interaction smoothers modeling the lateral bud burst development of *P. tremula* L. x *P. tremuloides* trees during GCE3 under constant long-day (LD) light, temperature, and relative humidity conditions (i.e., 18:6 hours light/dark cycles; 18 °C; and 80% relative humidity, respectively). The colored solid lines, given for the reference WT^{T89} and the independently RNAi transformed transgenic lines, represent the predicted probability to have a bud in a certain lateral bud burst stage at a particular moment in time. The shaded bands around the colored solid lines represent 95% pointwise confidence intervals. The lateral bud burst was scored following UPOV [1] using the following scoring values: dormant buds enveloped by scales (1; red), swelling buds with diverging scales (2; orange), sprouting buds (3; light-green), opened buds with leaves clustered (4; teal), diverging leaves with rolled op blades (5; blue), and completely unfolded leaves (6; purple). Doy is the abbreviation for day of the year. Doy counts started on the 1st of January 2015.

Growth Chamber Experiment 4 (GCE4)

Primary growth

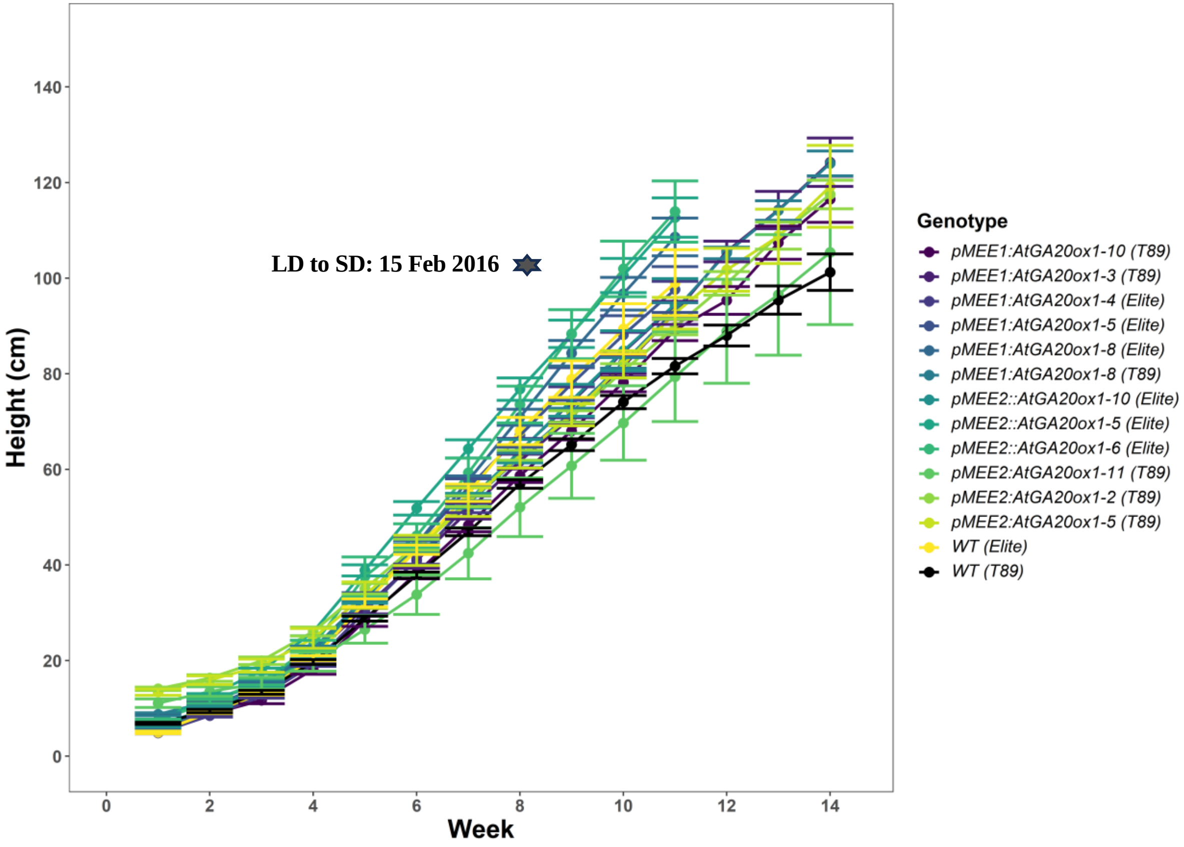

Figure S31. The primary growth of *P. tremula* L. x *P. tremuloides* trees during GCE4 under constant long-day (LD) light, temperature, and relative humidity conditions (i.e., 18:6 hours light/dark cycles; 18 °C; and 80% relative humidity, respectively). Light conditions were changed to short-day (SD) conditions (i.e., 16:8 hours light/dark cycles) on the 15th of February 2016 (Week = 8). The black solid line represents the primary growth of the reference WT^{T89} while the colored solid lines represent the primary growth of independently RNAi transformed transgenic lines. Dots and error bars represent mean and standard error values, respectively. Week counts started on the 3rd week of December 2015.

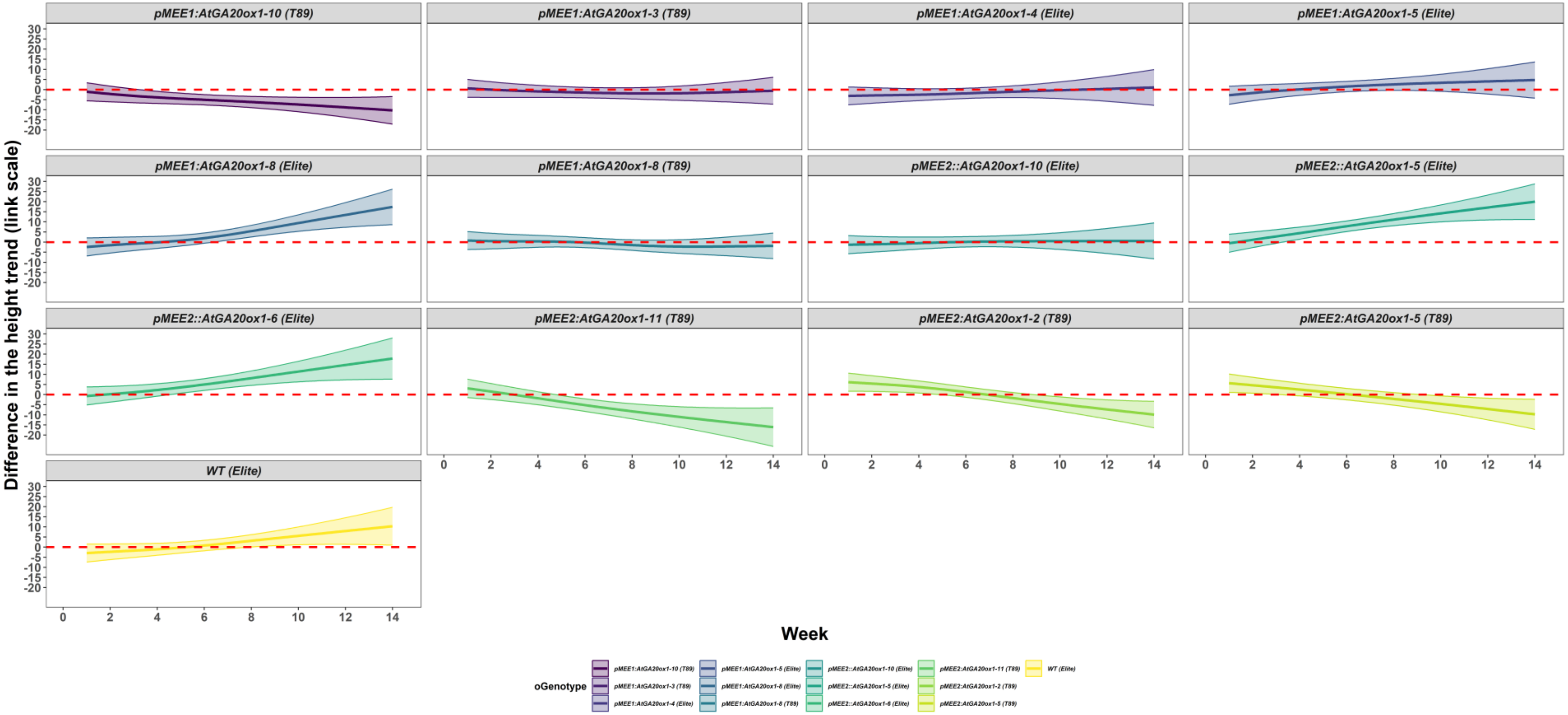

Figure S32. Term plots of the generalized additive (mixed) models with ordered-factor-smooth interaction smoothers modeling the mean primary growth of *P. tremula* L. x *P. tremuloides* trees during GCE4 under constant long-day (LD) light, temperature, and relative humidity conditions (i.e., 18:6 hours light/dark cycles; 18 °C; and 80% relative humidity, respectively). Light conditions were changed to short-day (SD) conditions (i.e., 16:8 hours light/dark cycles) on the 15th of February 2016 (Week = 8). The mean primary growth of the reference WT^{T89} is represented by the horizontal red dotted line. In contrast, the mean primary growth of the independently RNAi transformed transgenic lines is represented by colored solid lines. The shaded bands around the smooth terms represent Bayesian Wabha/Silverman credible intervals. Week counts started on the 3rd week of December 2015.

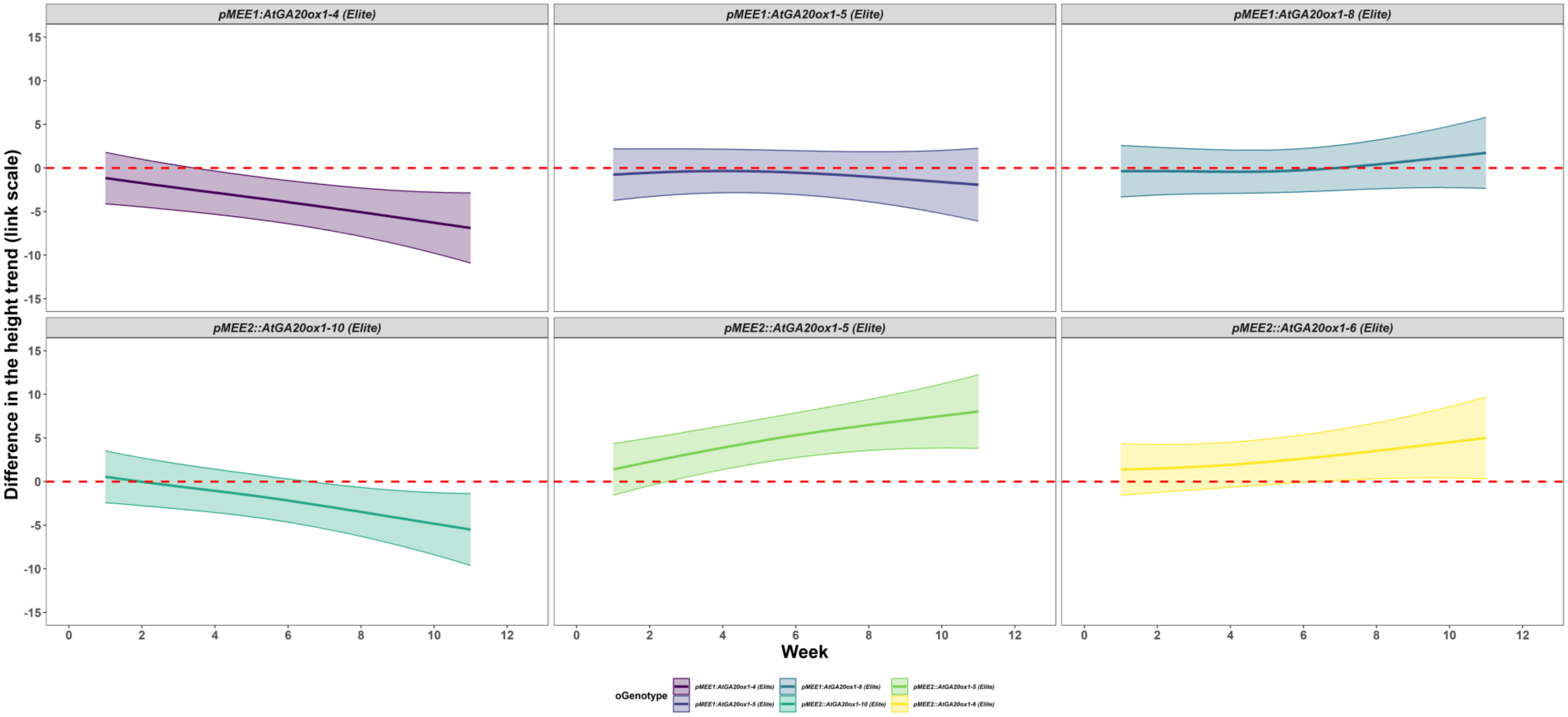

Figure S33. Term plots of the generalized additive (mixed) models with ordered-factor-smooth interaction smoothers modeling the mean primary growth of *P. tremula* L. x *P. tremuloides* trees during GCE4 under constant long-day (LD) light, temperature, and relative humidity conditions (i.e., 18:6 hours light/dark cycles; 18 °C; and 80% relative humidity, respectively). Light conditions were changed to short-day (SD) conditions (i.e., 16:8 hours light/dark cycles) on the 15th of February 2016 (Week = 8). The mean primary growth of the reference WT^{Elite} is represented by the horizontal red dotted line. In contrast, the mean primary growth of the independently RNAi transformed transgenic lines is represented by colored solid lines. The shaded bands around the smooth terms represent Bayesian Wabha/Silverman credible intervals. Week counts started on the 3rd week of December 2015.

Secondary growth

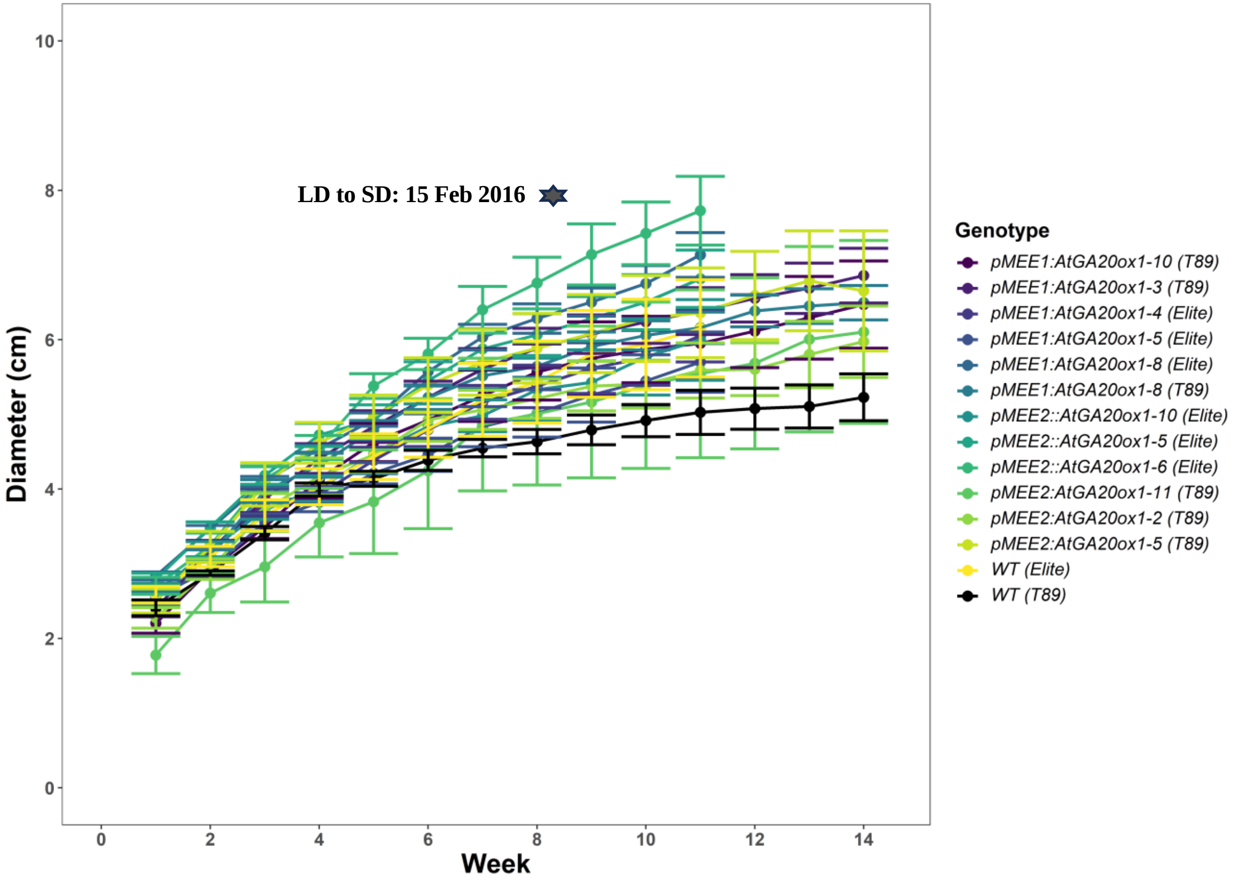

Figure S34. The secondary growth of *P. tremula* L. x *P. tremuloides* trees during GCE4 under constant long-day (LD) light, temperature, and relative humidity conditions (i.e., 18:6 hours light/dark cycles; 18 °C; and 80% relative humidity, respectively). Light conditions were changed to short-day (SD) conditions (i.e., 16:8 hours light/dark cycles) on the 15th of February 2016 (Week = 8). The black solid line represents the secondary growth of the reference WT^{T89} while the colored solid lines represent the secondary growth of independently RNAi transformed transgenic lines. Dots and error bars represent mean and standard error values, respectively. Week counts started on the 3rd week of December 2015.

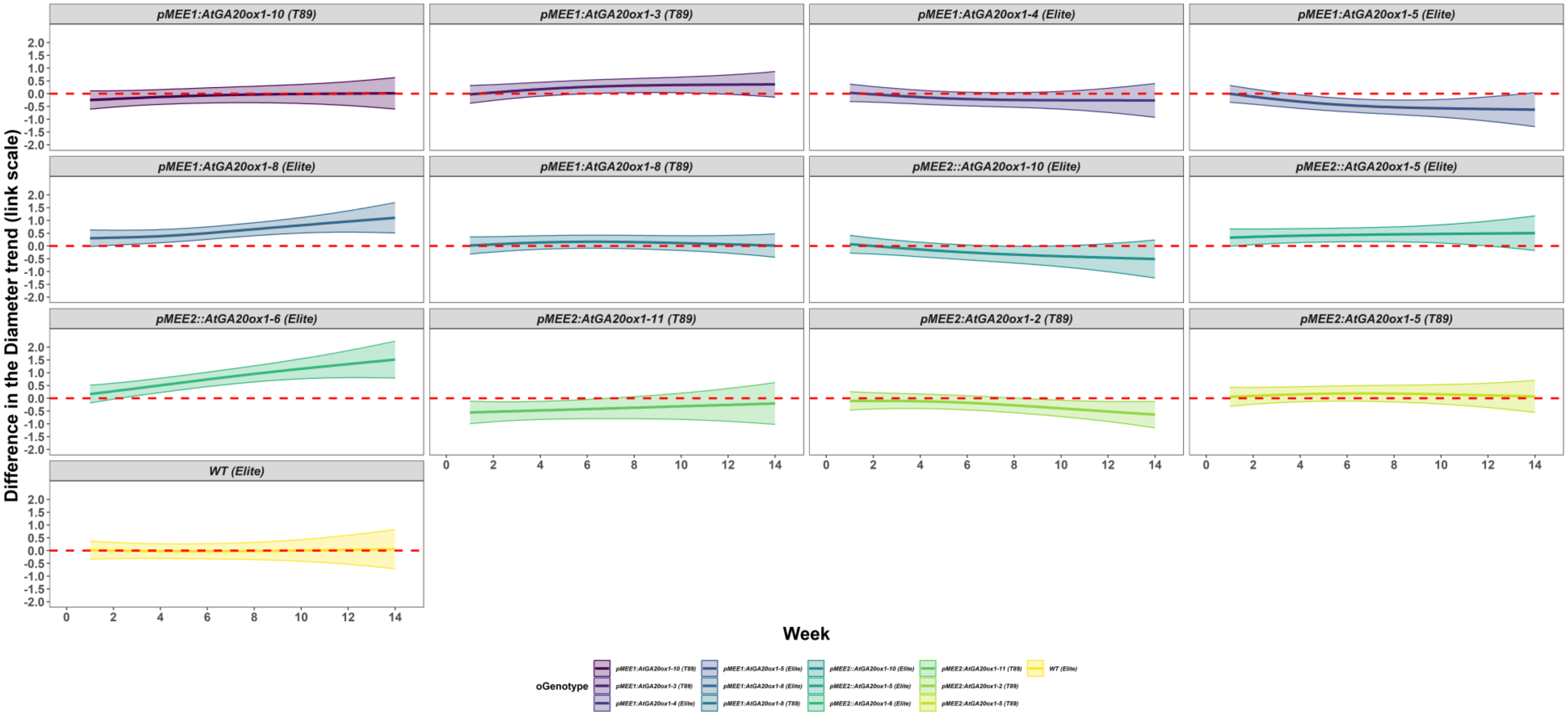

Figure S35. Term plots of the generalized additive (mixed) models with ordered-factor-smooth interaction smoothers modeling the mean secondary growth of *P. tremula* L. x *P. tremuloides* trees during GCE4 under constant long-day (LD) light, temperature, and relative humidity conditions (i.e., 18:6 hours light/dark cycles; 18 °C; and 80% relative humidity, respectively). Light conditions were changed to short-day (SD) conditions (i.e., 16:8 hours light/dark cycles) on the 15th of February 2016 (Week = 8). The mean secondary growth of the reference WT^{T89} is represented by the horizontal red dotted line. In contrast, the mean secondary growth of the independently RNAi transformed transgenic lines is represented by colored solid lines. The shaded bands around the smooth terms represent Bayesian Wabha/Silverman credible intervals. Week counts started on the 3rd week of December 2015.

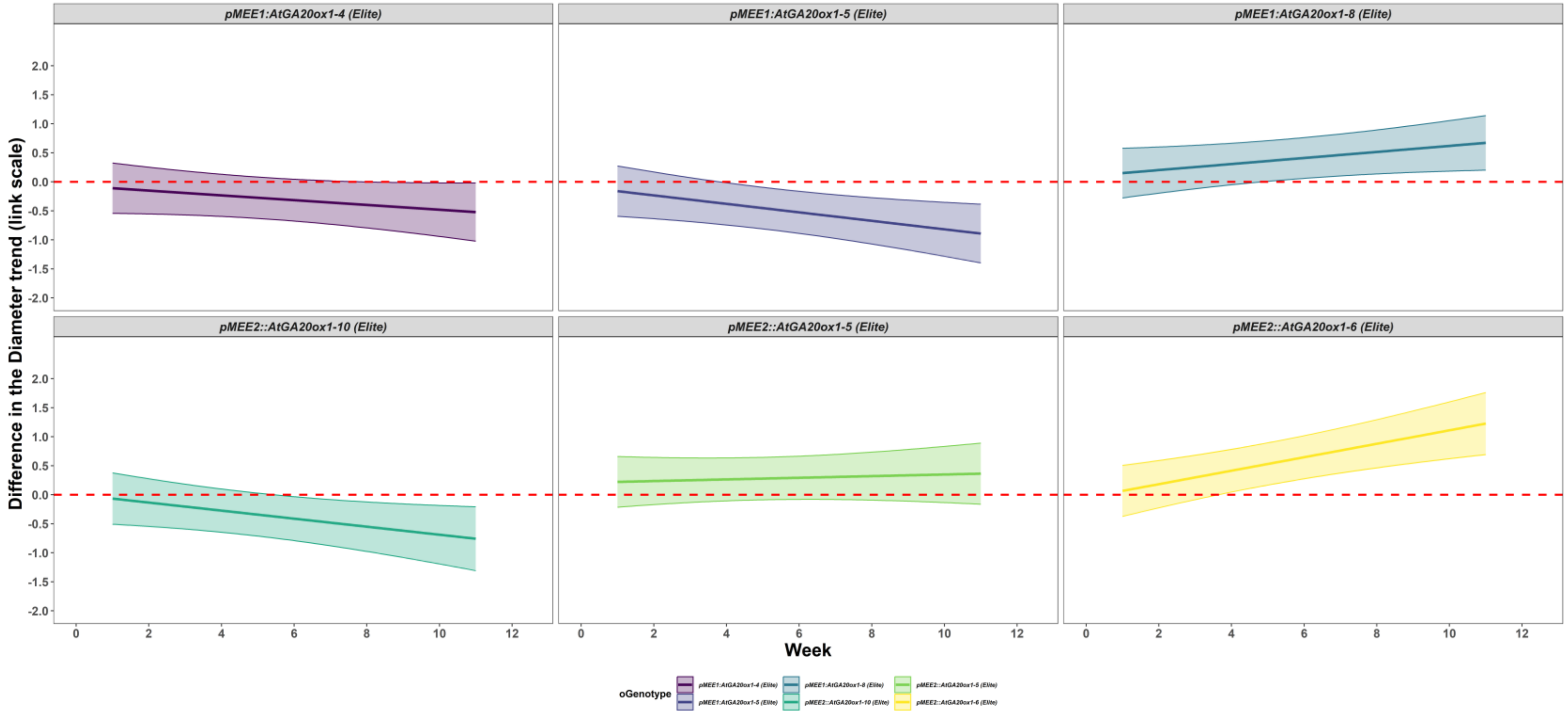

Figure S36. Term plots of the generalized additive (mixed) models with ordered-factor-smooth interaction smoothers modeling the mean secondary growth of *P. tremula* L. x *P. tremuloides* trees during GCE4 under constant long-day (LD) light, temperature, and relative humidity conditions (i.e., 18:6 hours light/dark cycles; 18 °C; and 80% relative humidity, respectively). Light conditions were changed to short-day (SD) conditions (i.e., 16:8 hours light/dark cycles) on the 15th of February 2016 (Week = 8). The mean secondary growth of the reference WT^{Elite} is represented by the horizontal red dotted line. In contrast, the mean secondary growth of the independently RNAi transformed transgenic lines is represented by colored solid lines. The shaded bands around the smooth terms represent Bayesian Wabha/Silverman credible intervals. Week counts started on the 3rd week of December 2015.

Bud set

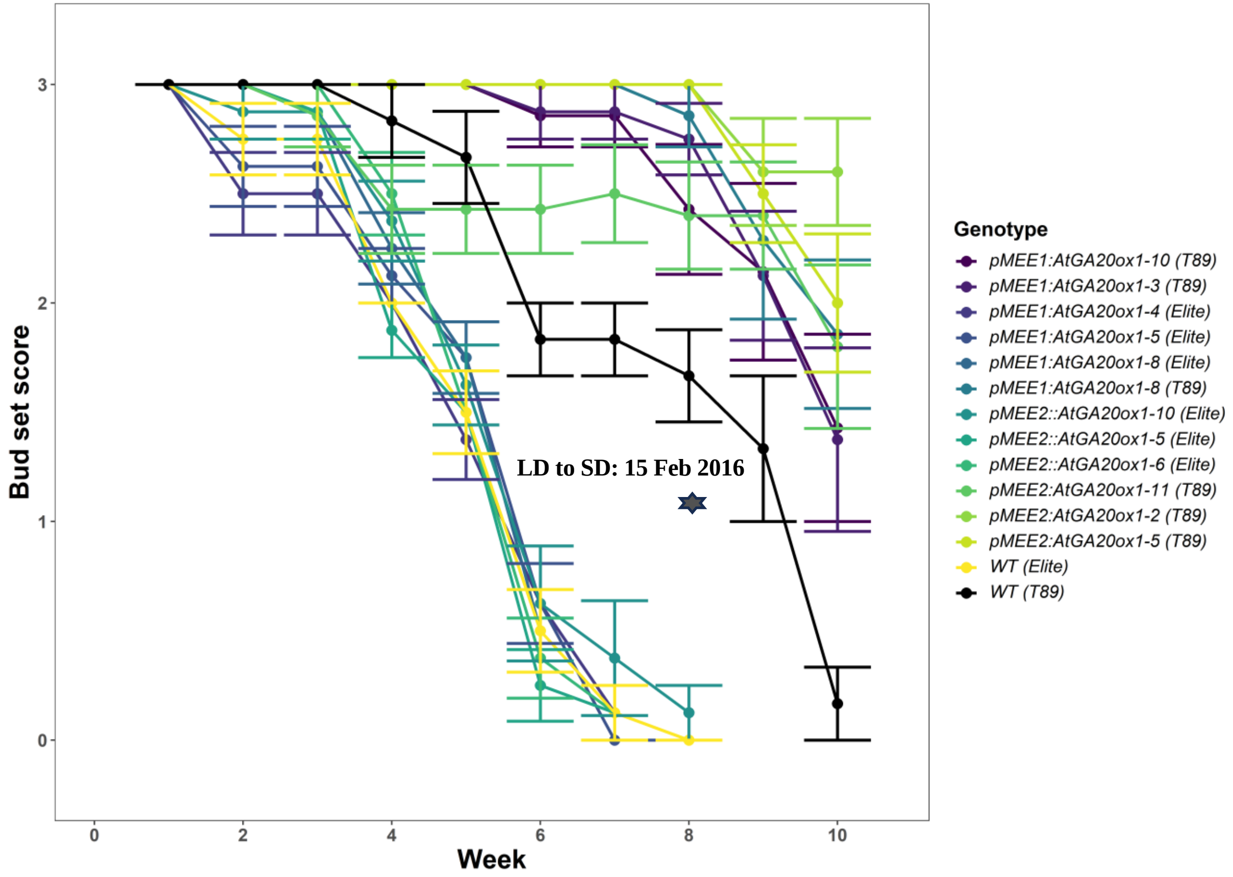

Figure S37. The bud set development in in *P. tremula* L. x *P. tremuloides* trees during GCE4 under constant short-day (SD) conditions (i.e., 15:9 hours light/dark cycles) on the 15th of February 2016 (Week = 8). The black solid line represents the bud set stage of the reference WT^{T89} while the colored solid lines represent the bud set stage of independently RNAi transformed transgenic lines. Dots and error bars represent mean and standard error values, respectively. The bud set was scored following UPOV [1] and Ibanez, et al. [2] using the following (opposite) scoring values: a still actively growing shoot and uninitiated bud set (3), initiation of the bud set and cessation of growth (2), formation of the buds (1), and completed bud set (0)[2-4]. Week counts started on the 3rd week of December 2015.

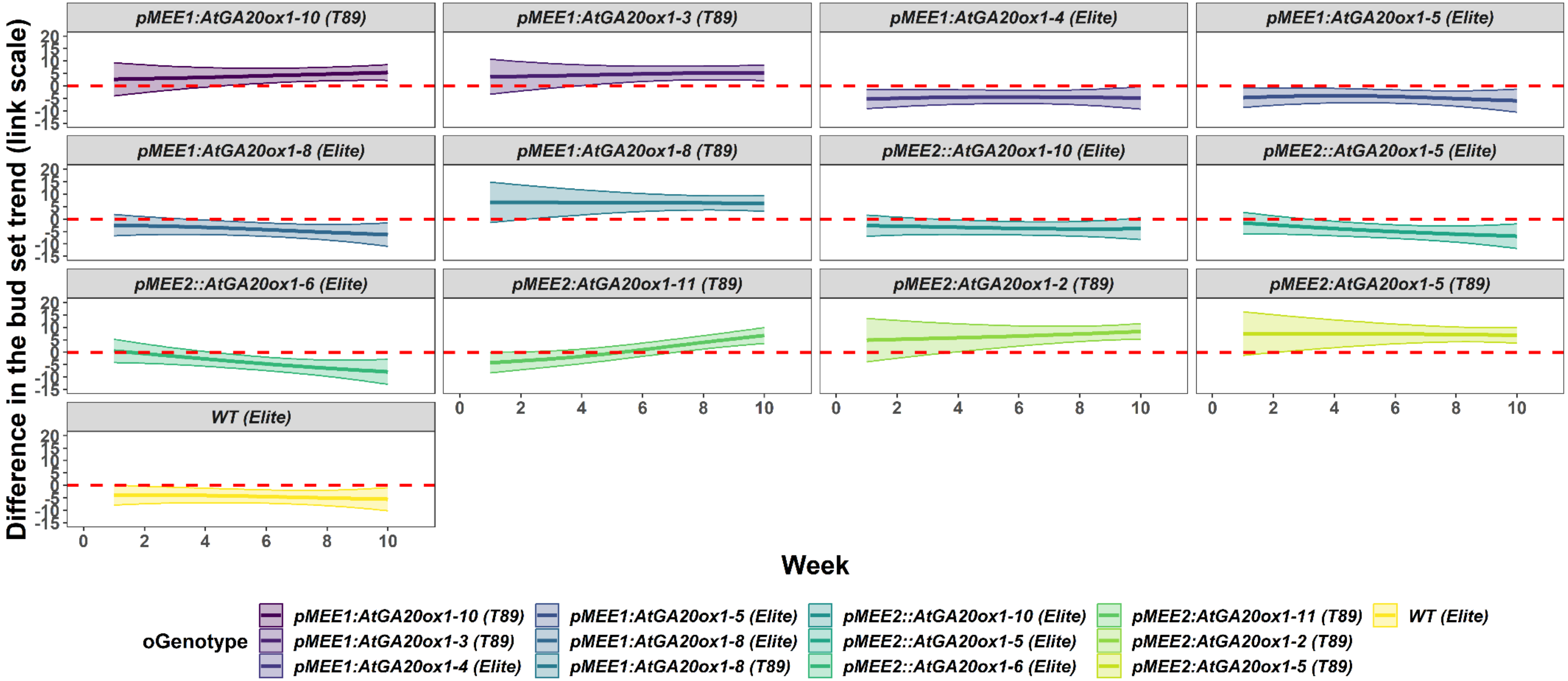

Figure S38. Term plots of the generalized additive (mixed) models with ordered-factor-smooth interaction smoothers modeling the bud set development of *P. tremula* L. x *P. tremuloides* trees during GCE4 under constant short-day SD conditions (i.e., 15:9 hours light/dark cycles; 18 °C; and 80% relative humidity, respectively). The mean bud set of the reference WT^{T89} is represented by the horizontal red dotted line. In contrast, the mean bud set of the independently RNAi transformed transgenic lines is represented by colored solid lines. The shaded bands around the smooth terms represent Bayesian Wabha/Silverman credible intervals. Week counts started on the 3rd week of December 2015.

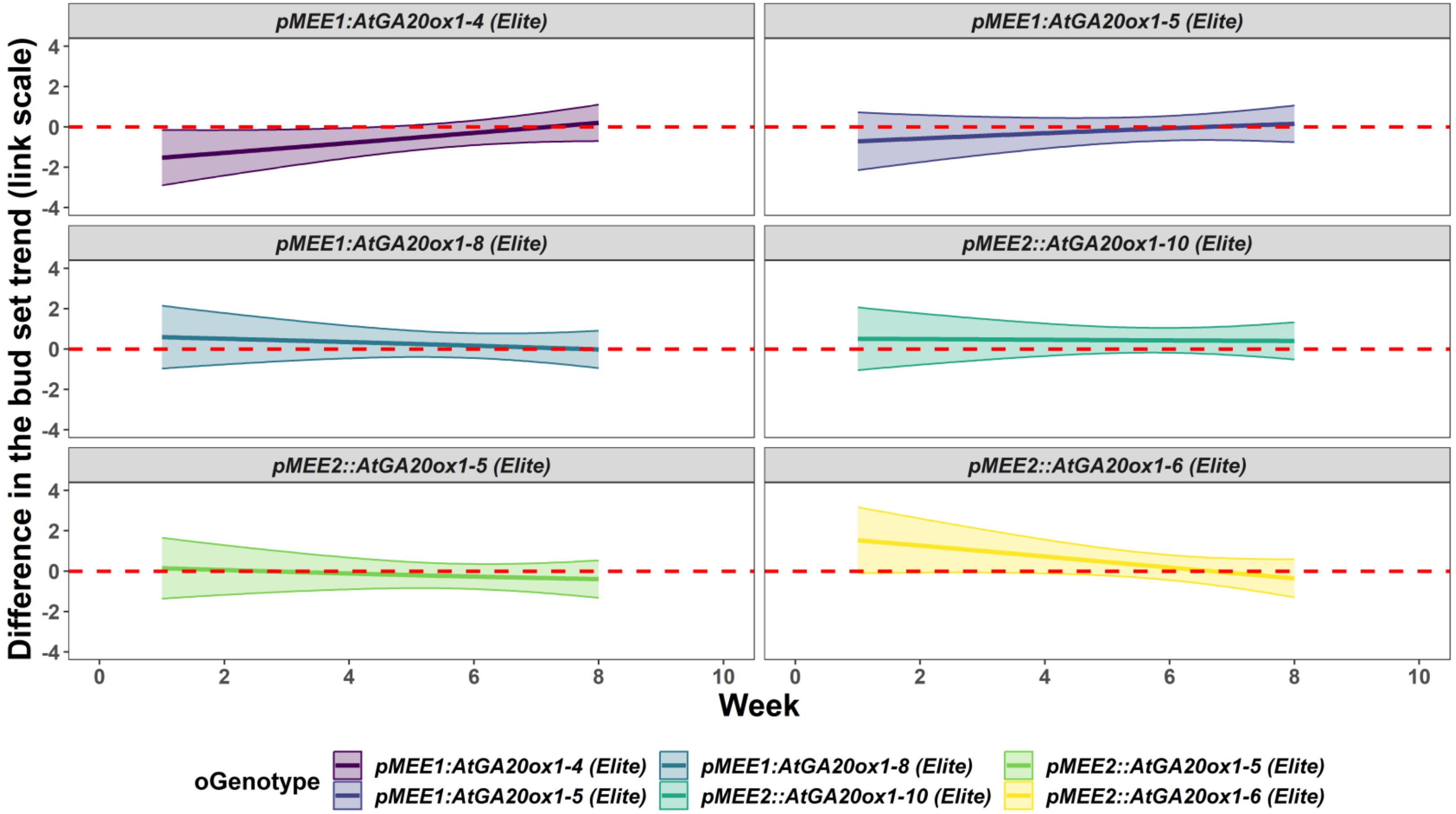

Figure S39. Term plots of the generalized additive (mixed) models with ordered-factor-smooth interaction smoothers modeling the bud set development of *P. tremula* L. x *P. tremuloides* trees during GCE4 under constant short-day SD conditions (i.e., 15:9 hours light/dark cycles; 18 °C; and 80% relative humidity, respectively). The mean bud set of the reference WT^{Elite} is represented by the horizontal red dotted line. In contrast, the mean bud set of the independently RNAi transformed transgenic lines is represented by colored solid lines. The shaded bands around the smooth terms represent Bayesian Wabha/Silverman credible intervals. Doy is the abbreviation for day of the year. Week counts started on the 3rd week of December 2015.

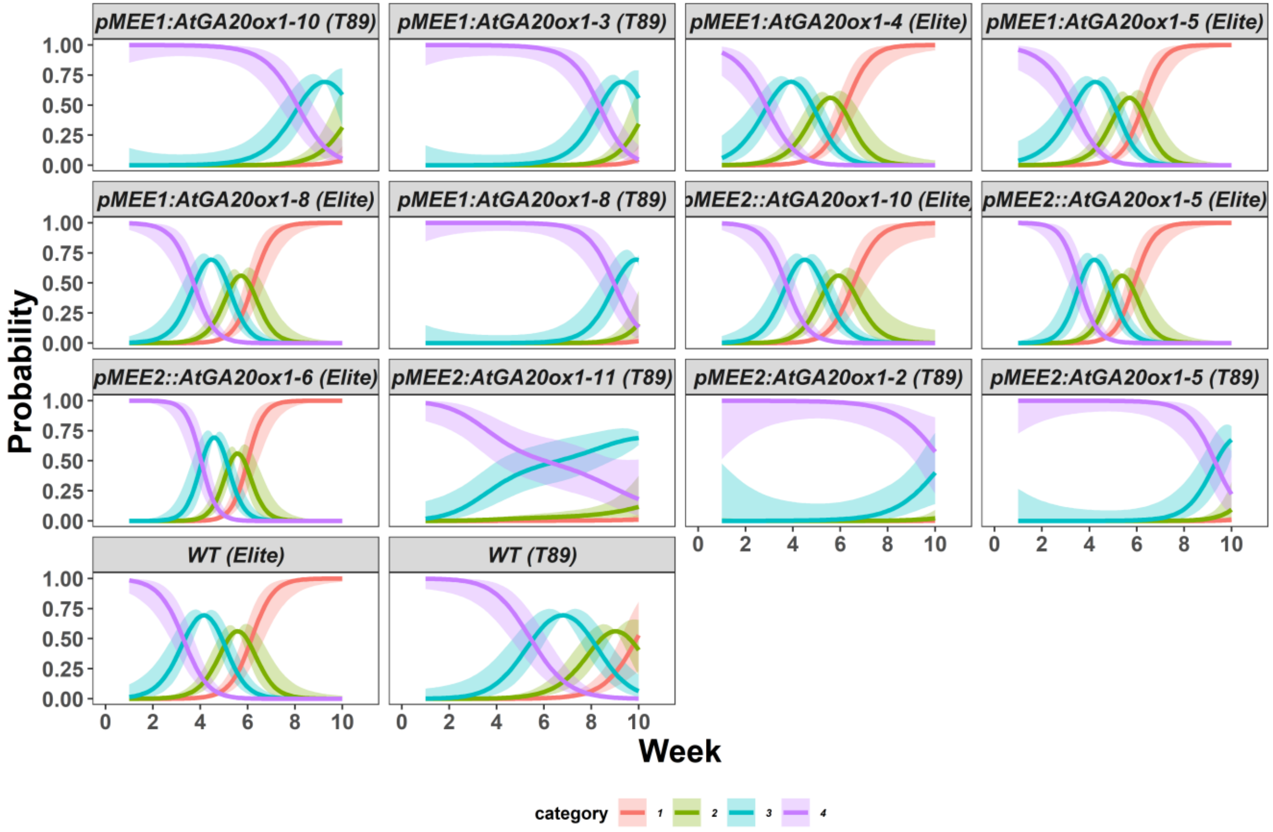

Figure S40. Term plots of the generalized additive (mixed) models with ordered-factor-smooth interaction smoothers modeling the bud set development of *P. tremula* L. x *P. tremuloides* trees during GCE4 under constant short-day (SD) conditions (i.e., 15:9 hours light/dark cycles; 18 °C; and 80% relative humidity, respectively). The colored solid lines, given for the reference WT^{T89} and the independently RNAi transformed transgenic lines, represent the predicted probability to have a bud in a certain bud set stage at a particular moment in time. The shaded bands around the colored solid lines represent 95% pointwise confidence intervals. The bud set was scored following UPOV [1] and Ibanez, et al. [2] using the following (opposite) scoring values: a still actively growing shoot and uninitiated bud set (4; purple), initiation of the bud set and cessation of growth (3; teal), formation of the buds (2; light-green), and completed bud set (1; red)[2-4]. Week counts started on the 3rd week of December 2015.

Apical bud burst

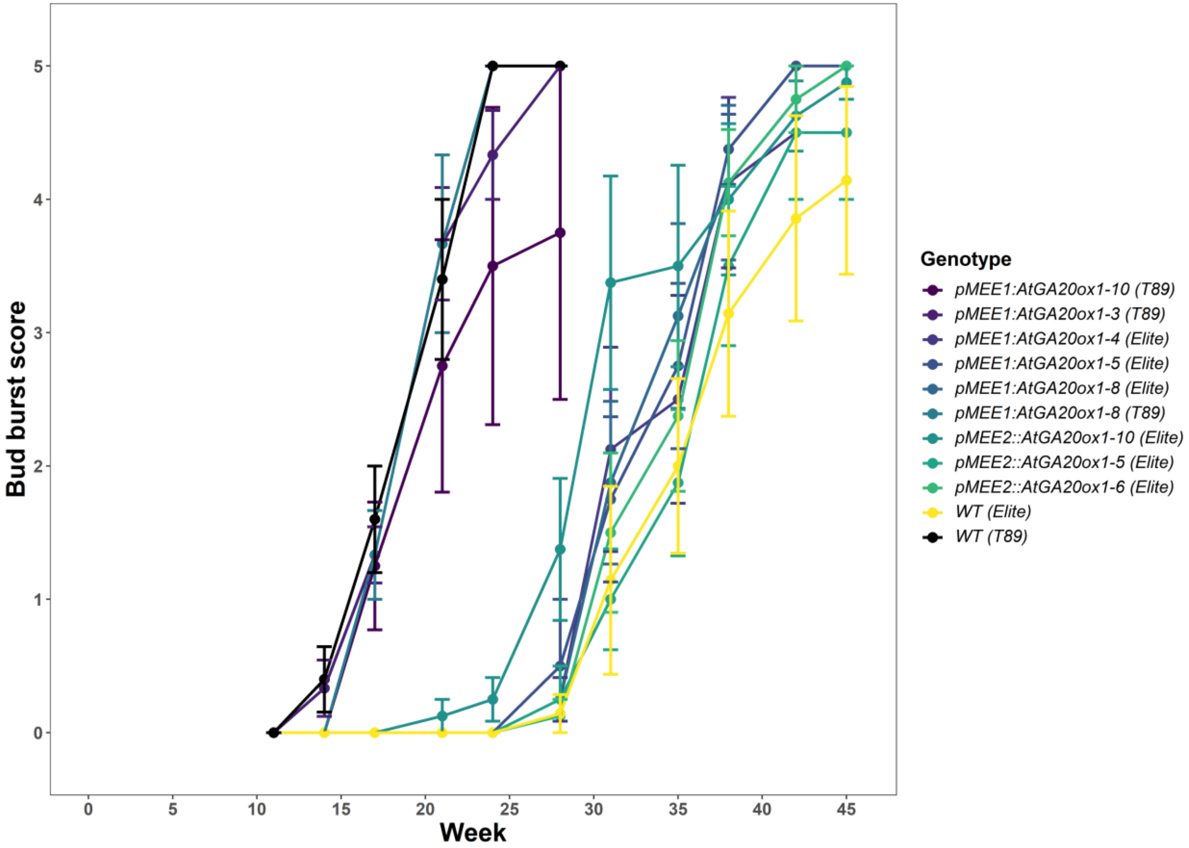

Figure S41. The apical bud burst development in in *P. tremula* L. x *P. tremuloides* trees during GCE4 under constant long-day (LD) conditions (i.e., 18:6 hours light/dark cycles; 18 °C; and 80% relative humidity, respectively). The black solid line represents the apical bud burst stage of the reference WT^{T89} while the colored solid lines represent the apical bud burst stage of independently RNAi transformed transgenic lines. Dots and error bars represent mean and standard error values, respectively. The apical bud burst was scored following UPOV [1] using the following scoring values: dormant buds enveloped by scales (0), swelling buds with diverging scales (1), sprouting buds (2), opened buds with leaves clustered (3), diverging leaves with rolled op blades (4), and completely unfolded leaves (5). Week counts started on the 3rd week of December 2015. No means and standard errors could be computed for $pMEE2::AtGA20ox1\text{-}11^{T89}$.

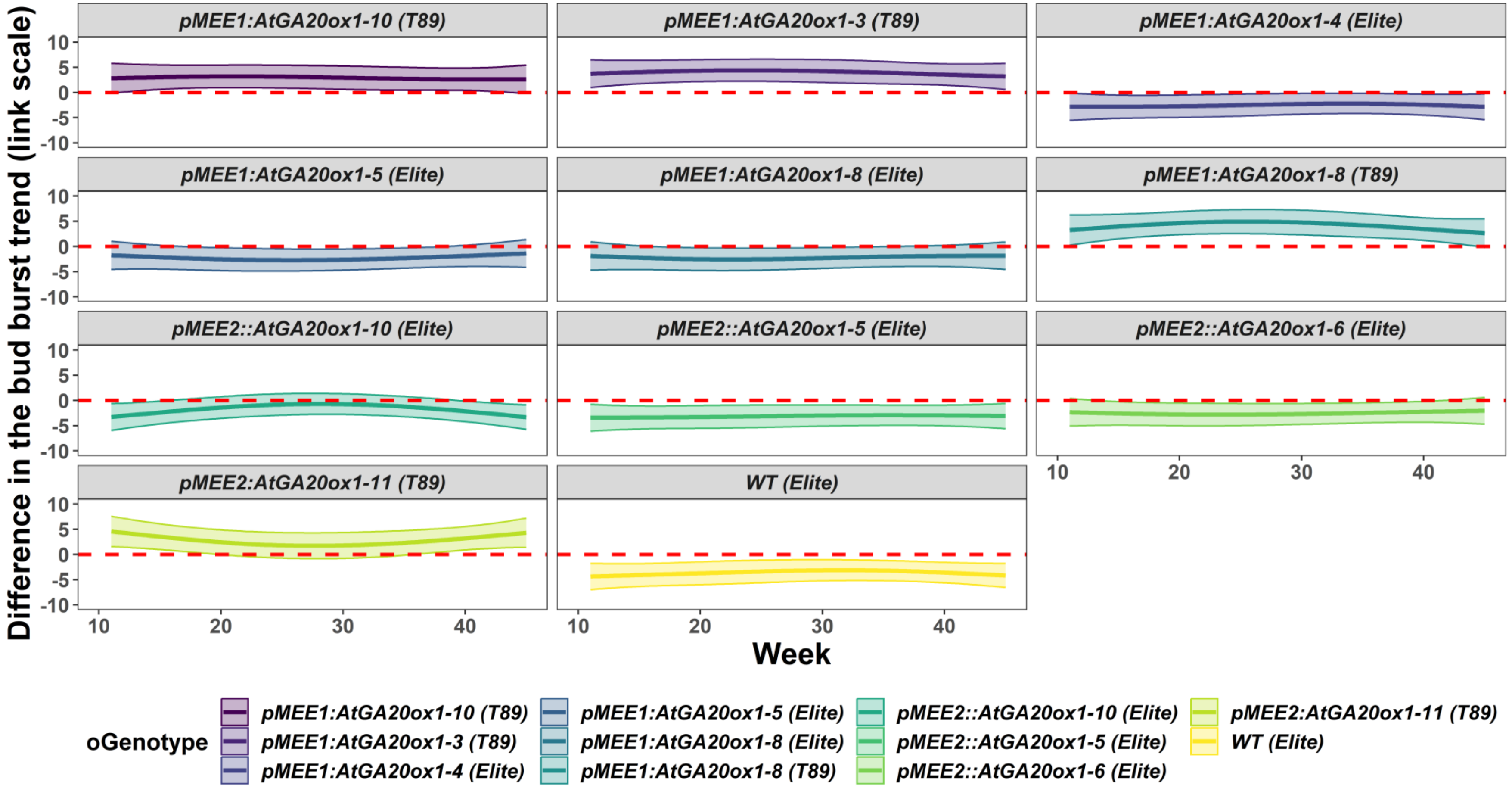

Figure 42. Term plots of the generalized additive (mixed) models with ordered-factor-smooth interaction smoothers modeling the apical bud burst development of *P. tremula* L. x *P. tremuloides* trees during GCE4 under constant long-day (LD) conditions (i.e., 18:6 hours light/dark cycles; 18 °C; and 80% relative humidity, respectively). The mean apical bud burst of the reference WT^{T89} is represented by the horizontal red dotted line. In contrast, the mean apical bud burst of the independently RNAi transformed transgenic lines is represented by colored solid lines. The shaded bands around the smooth terms represent Bayesian Wabha/Silverman credible intervals. Week counts started on the 3rd week of December 2015.

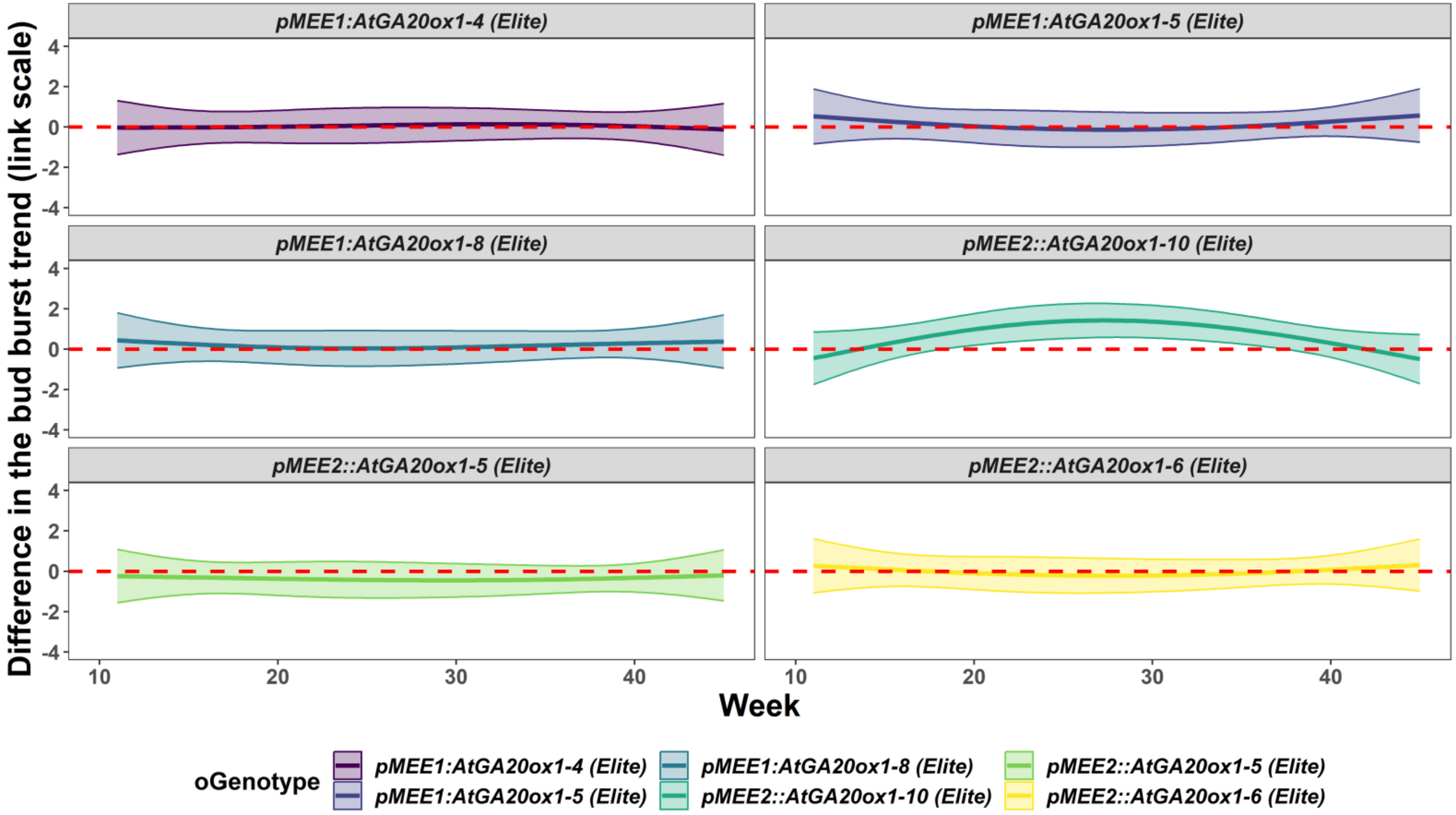

Figure S43. Term plots of the generalized additive (mixed) models with ordered-factor-smooth interaction smoothers modeling the apical bud burst development of *P. tremula* L. x *P. tremuloides* trees during GCE4 under constant long-day (LD) conditions (i.e., 18:6 hours light/dark cycles; 18 °C; and 80% relative humidity, respectively). The mean apical bud burst of the reference WT^{Elite} is represented by the horizontal red dotted line. In contrast, the mean apical bud burst of the independently RNAi transformed transgenic lines is represented by colored solid lines. The shaded bands around the smooth terms represent Bayesian Wabha/Silverman credible intervals. Week counts started on the 3rd week of December 2015.

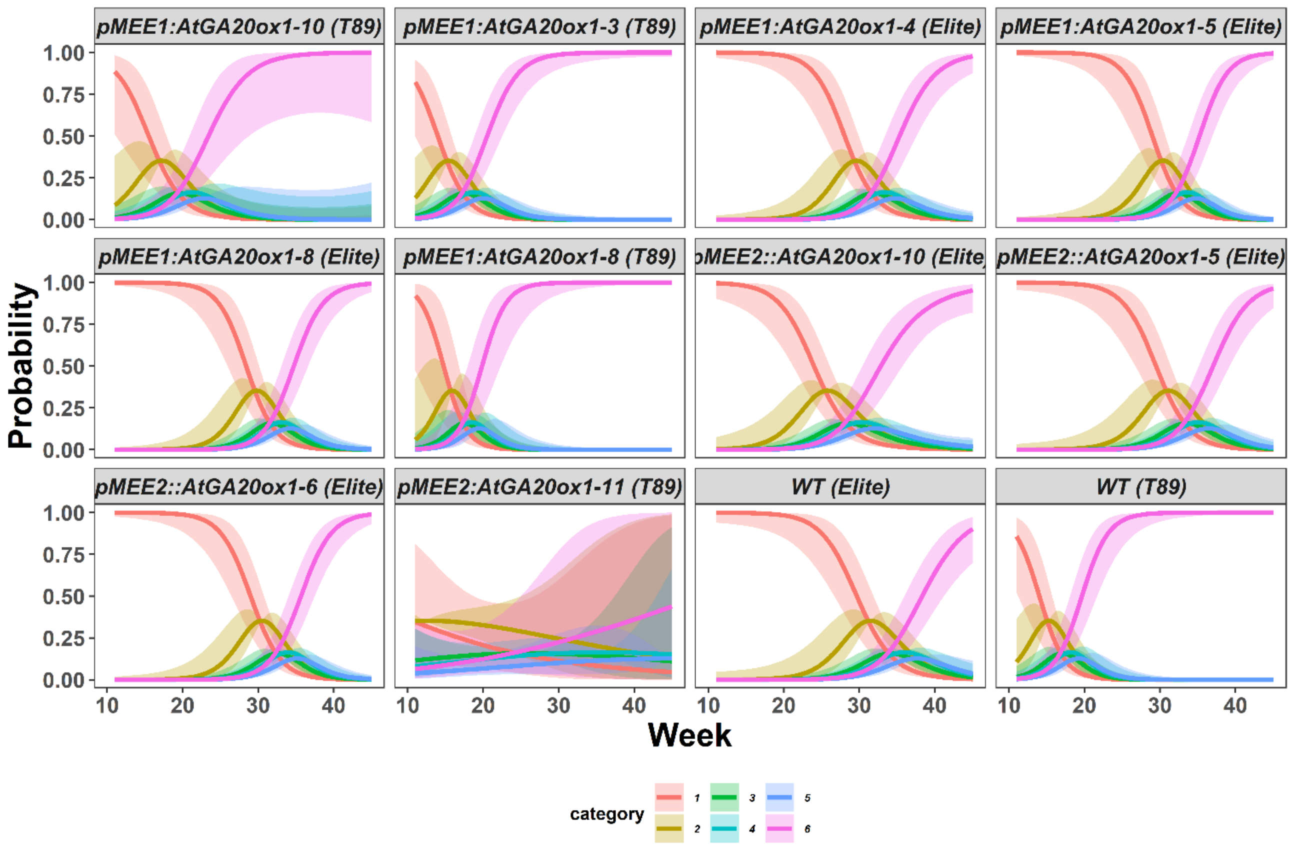

Figure S44. Term plots of the generalized additive (mixed) models with ordered-factor-smooth interaction smoothers modeling the apical bud burst development of *P. tremula* L. x *P. tremuloides* trees during GCE4 under constant long-day (LD) conditions (i.e., 18:6 hours light/dark cycles; 18 °C; and 80% relative humidity, respectively). The colored solid lines, given for the reference WT^{T89} and the independently RNAi transformed transgenic lines, represent the predicted probability to have a bud in a certain apical bud burst stage at a particular moment in time. The shaded bands around the colored solid lines represent 95% pointwise confidence intervals. The apical bud burst was scored following UPOV [1] using the following scoring values: dormant buds enveloped by scales (1; red), swelling buds with diverging scales (2; orange), sprouting buds (3; light-green), opened buds with leaves clustered (4; teal), diverging leaves with rolled op blades (5; blue), and completely unfolded leaves (6; purple). Week counts started on the 3rd week of December 2015.

Lateral bud burst

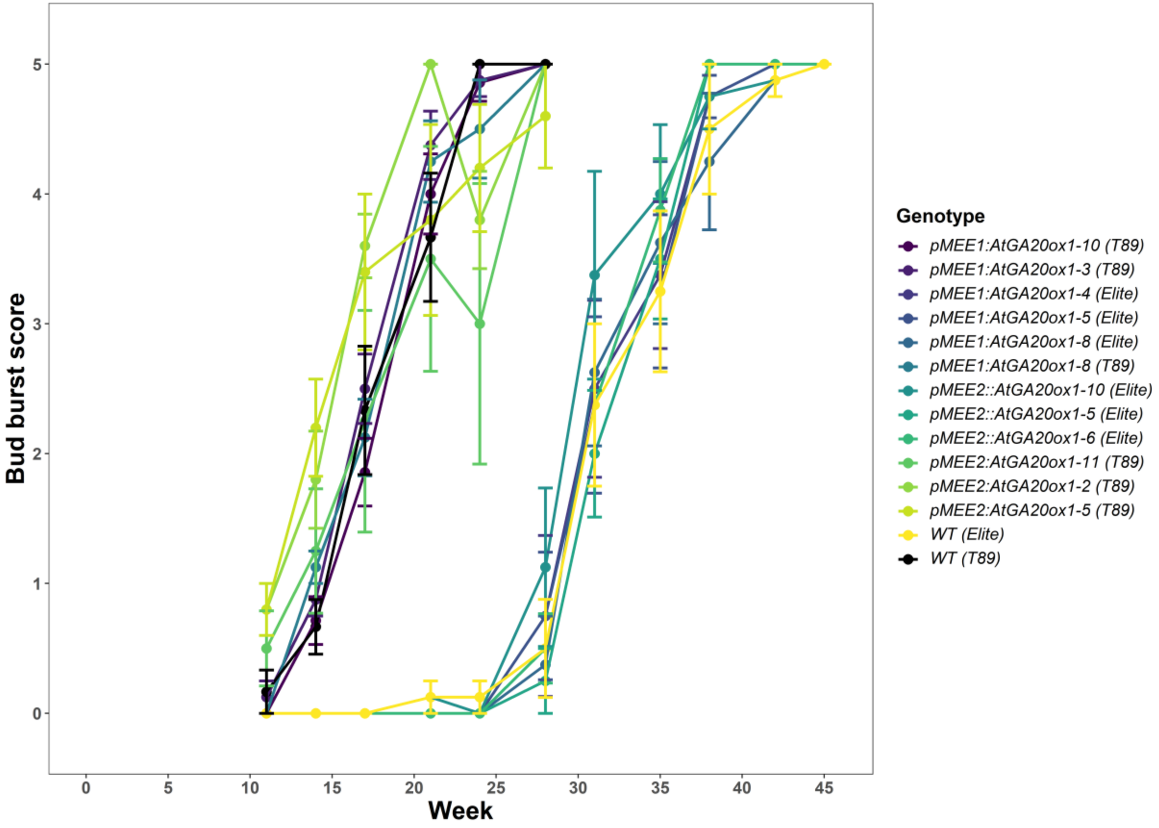

Figure S45. The lateral bud burst development in in *P. tremula* L. x *P. tremuloides* trees during GCE4 under constant long-day (LD) conditions (i.e., 18:6 hours light/dark cycles; 18 °C; and 80% relative humidity, respectively). The black solid line represents the lateral bud burst stage of the reference WT^{T89} while the colored solid lines represent the lateral bud burst stage of independently RNAi transformed transgenic lines. Dots and error bars represent mean and standard error values, respectively. The lateral bud burst was scored following UPOV [1] using the following scoring values: dormant buds enveloped by scales (0), swelling buds with diverging scales (1), sprouting buds (2), opened buds with leaves clustered (3), diverging leaves with rolled op blades (4), and completely unfolded leaves (5). Week counts started on the 3rd week of December 2015.

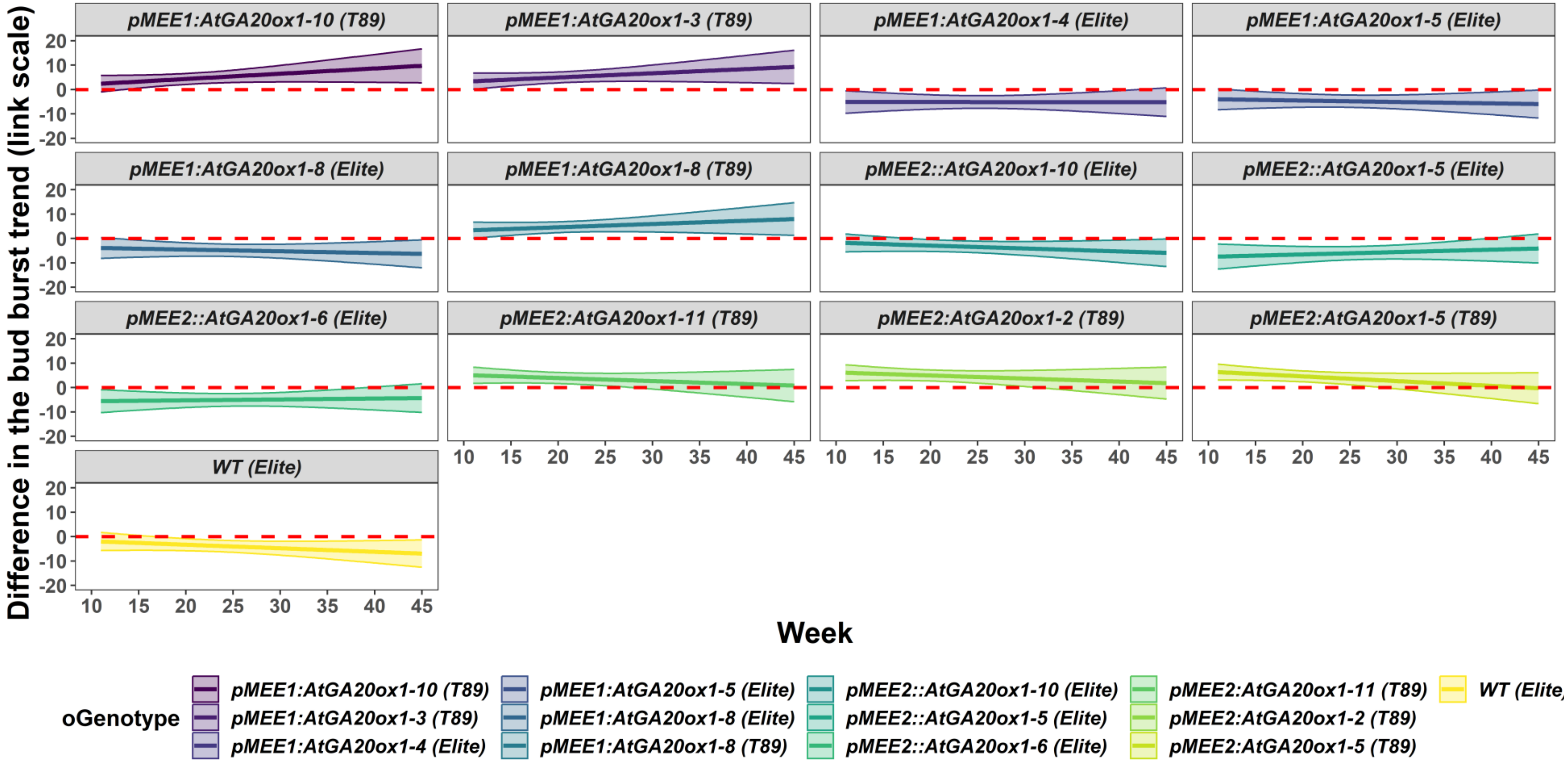

Figure S46. Term plots of the generalized additive (mixed) models with ordered-factor-smooth interaction smoothers modeling the lateral bud burst development of *P. tremula* L. x *P. tremuloides* trees during GCE4 under constant long-day (LD) conditions (i.e., 18:6 hours light/dark cycles; 18 °C; and 80% relative humidity, respectively). The mean lateral bud burst of the reference WT^{T89} is represented by the horizontal red dotted line. In contrast, the mean lateral bud burst of the independently RNAi transformed transgenic lines is represented by colored solid lines. The shaded bands around the smooth terms represent Bayesian Wabha/Silverman credible intervals. Week counts started on the 3rd week of December 2015.

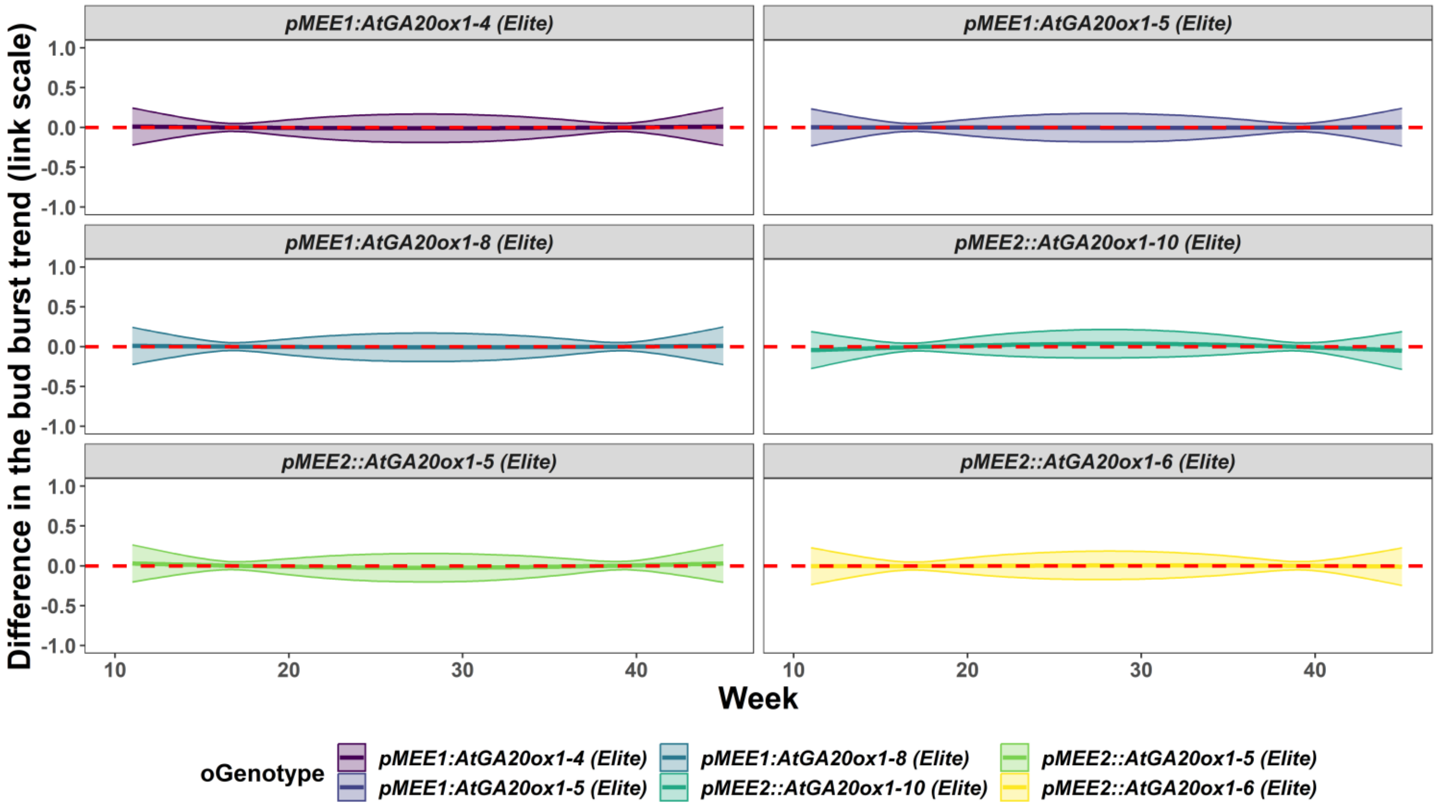

Figure S47. Term plots of the generalized additive (mixed) models with ordered-factor-smooth interaction smoothers modeling the lateral bud burst development of *P. tremula* L. x *P. tremuloides* trees during GCE4 under constant long-day (LD) conditions (i.e., 18:6 hours light/dark cycles; 18 °C; and 80% relative humidity, respectively) on the 15th of February 2016 (Week = 8). The mean lateral bud burst of the reference WT^{Elite} is represented by the horizontal red dotted line. In contrast, the mean lateral bud burst of the independently RNAi transformed transgenic lines is represented by colored solid lines. The shaded bands around the smooth terms represent Bayesian Wabha/Silverman credible intervals. Week counts started on the 3rd week of December 2015.

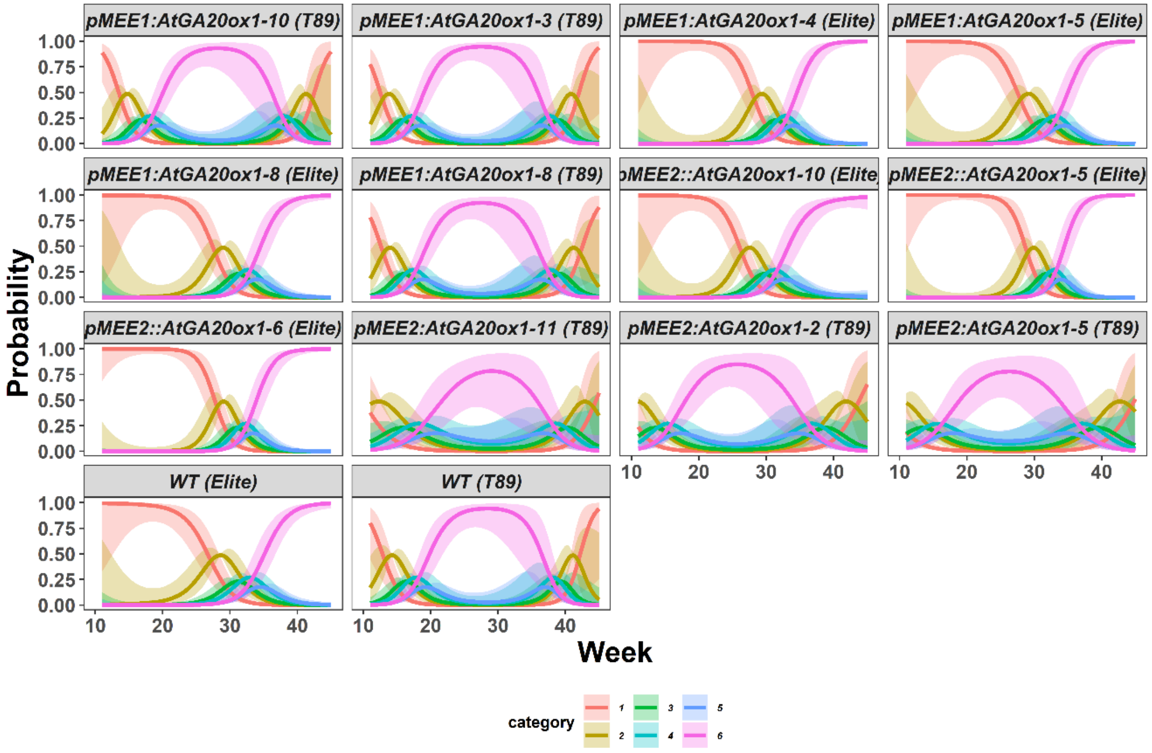

Figure S48. Term plots of the generalized additive (mixed) models with ordered-factor-smooth interaction smoothers modeling the lateral bud burst development of *P. tremula* L. x *P. tremuloides* trees during GCE4 under constant long-day (LD) conditions (i.e., 16:8 hours light/dark cycles; 18 °C; and 80% relative humidity, respectively). The colored solid lines, given for the reference WT^{T89} and the independently RNAi transformed transgenic lines, represent the predicted probability to have a bud in a certain lateral bud burst stage at a particular moment in time. The shaded bands around the colored solid lines represent 95% pointwise confidence intervals. The lateral bud burst was scored following UPOV [1] using the following scoring values: dormant buds enveloped by scales (1; red), swelling buds with diverging scales (2; orange), sprouting buds (3; light-green), opened buds with leaves clustered (4; teal), diverging leaves with rolled op blades (5; blue), and completely unfolded leaves (6; purple). Week counts started on the 3rd week of December 2015.

Growth Chamber Experiment 5 (GCE5)

Primary growth

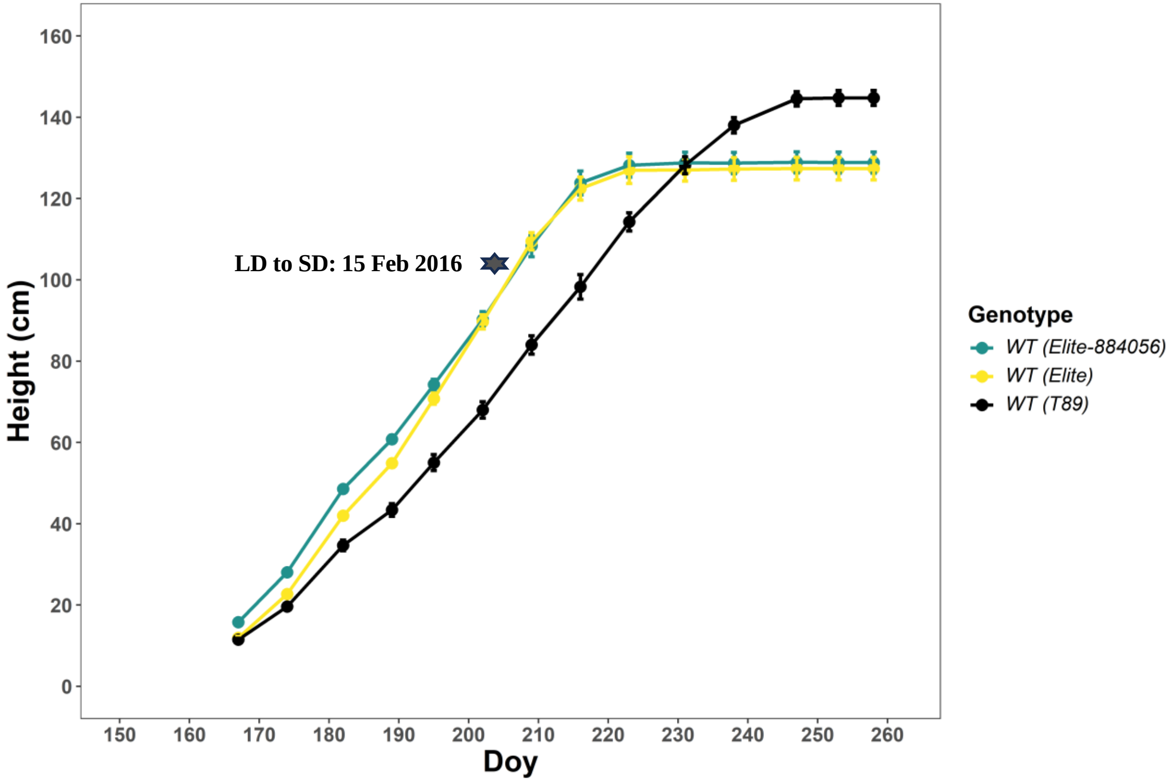

Figure S49. The primary growth of *P. tremula* L. x *P. tremuloides* trees during GCE5 under constant long-day (LD) light, temperature, and relative humidity conditions (i.e., 18:6 hours light/dark cycles; 18 °C; and 80% relative humidity, respectively). Light conditions were changed to short-day (SD) conditions (i.e., 16:8 hours light/dark cycles) on the 14th of July 2016 (Doy = 196). The black solid line represents the primary growth of the reference WT^{T89} while the colored solid lines represent the primary growth of independently RNAi transformed transgenic lines. Dots and error bars represent mean and standard error values, respectively. Doy is the abbreviation for day of the year. Doy counts started on the 1st of January 2016.

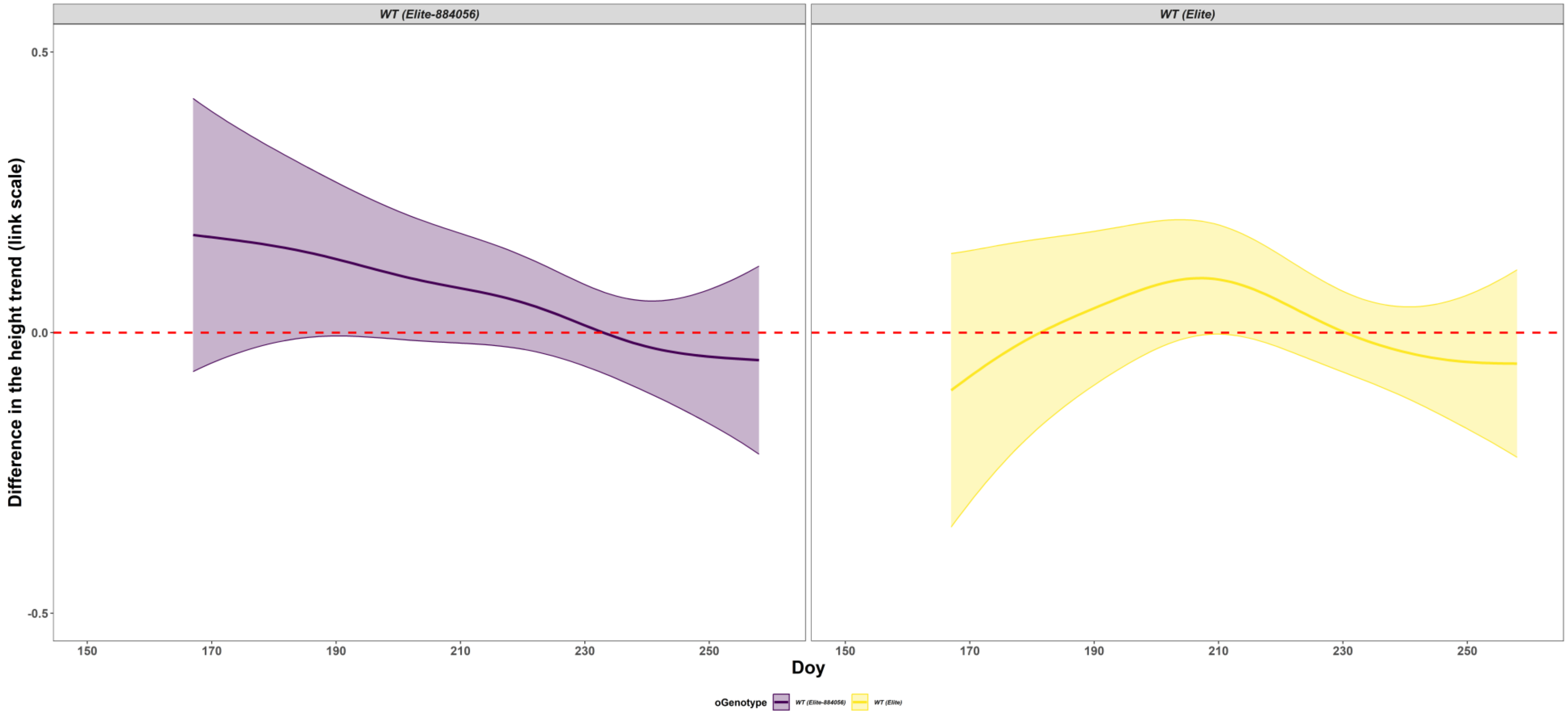

Figure S50. Term plots of the generalized additive (mixed) models with ordered-factor-smooth interaction smoothers modeling the mean primary growth of *P. tremula* L. x *P. tremuloides* trees during GCE5 under constant long-day (LD) light, temperature, and relative humidity conditions (i.e., 18:6 hours light/dark cycles; 18 °C; and 80% relative humidity, respectively). Light conditions were changed to short-day (SD) conditions (i.e., 16:8 hours light/dark cycles) on the 14th of July 2016 (Doy = 196). The mean primary growth of the reference WT^{T89} is represented by the horizontal red dotted line. In contrast, the mean primary growth of the independently RNAi transformed transgenic lines is represented by colored solid lines. The shaded bands around the smooth terms represent Bayesian Wabha/Silverman credible intervals. Doy is the abbreviation for day of the year. Doy counts started on the 1st of January 2016.

Field Experiment 2 (FE2)

Primary growth

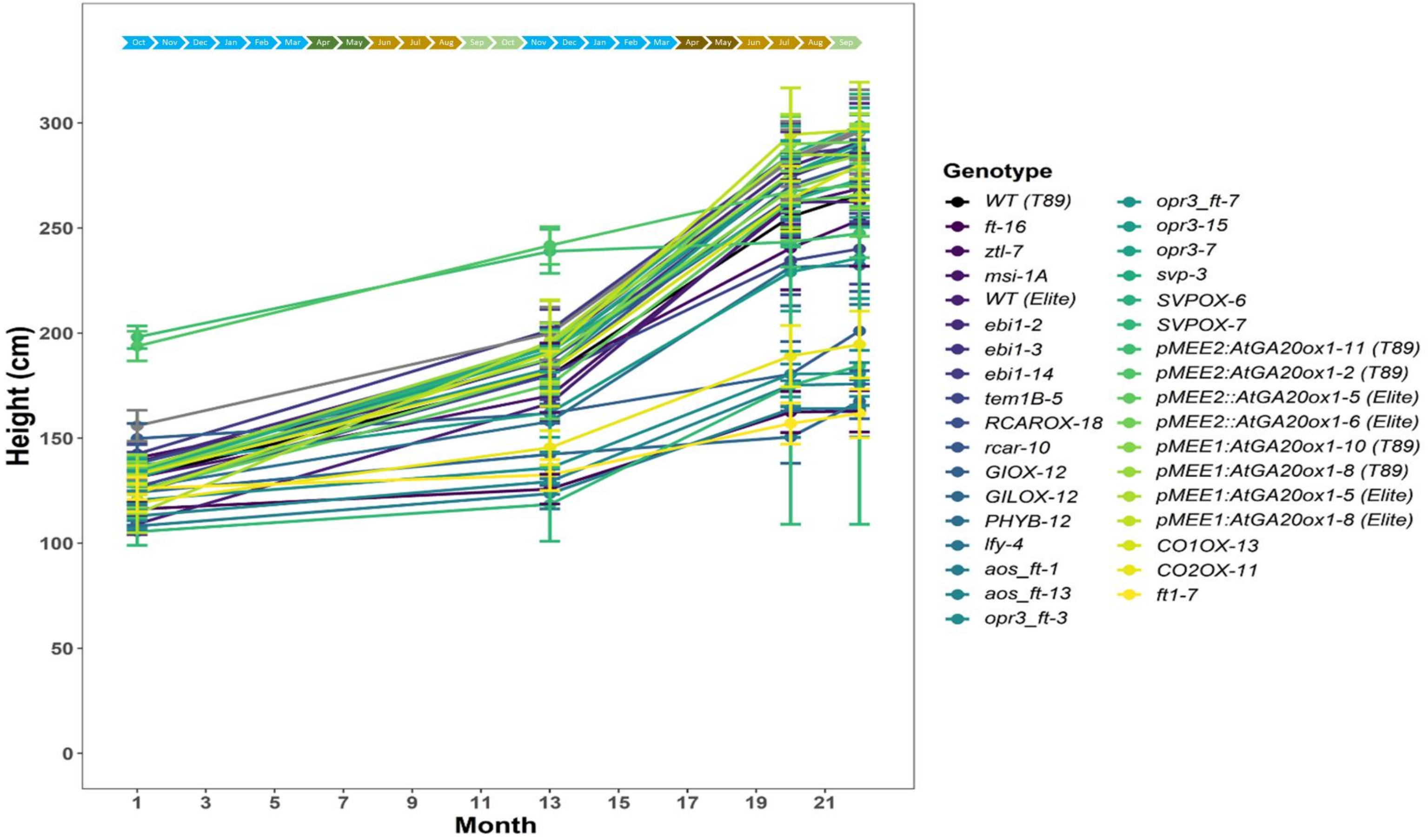

Figure S51. The primary growth of *P. tremula* L. x *P. tremuloides* trees during FE2. The black solid line represents the primary growth of the reference WT^{T89} while the colored solid lines represent the primary growth of independently RNAi transformed transgenic lines. Dots and error bars represent mean and standard error values, respectively. Month counts started in October 2018.

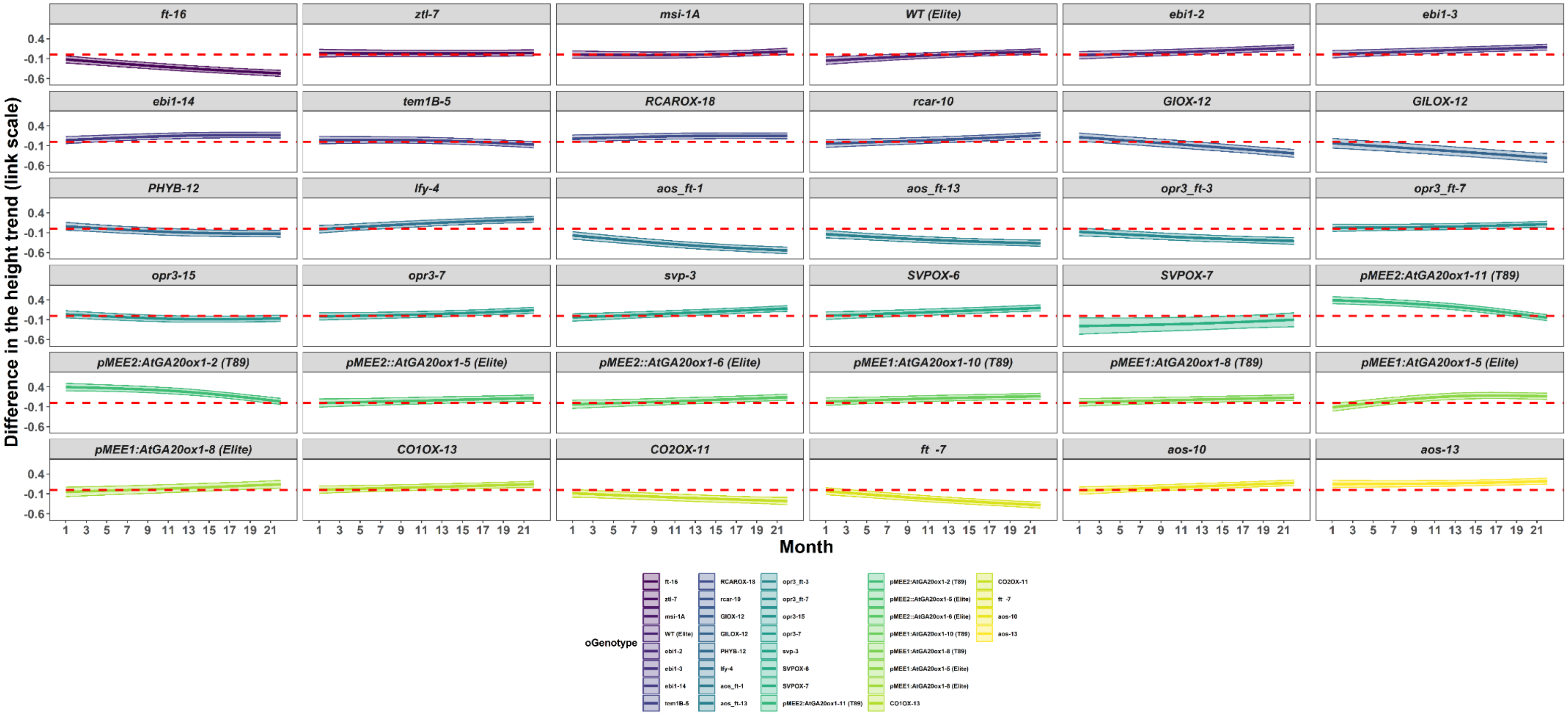

Figure S52. Term plots of the generalized additive (mixed) models with ordered-factor-smooth interaction smoothers modeling the mean primary growth of *P. tremula* L. x *P. tremuloides* trees during FE2. The mean primary growth of the reference WT^{T89} is represented by the horizontal red dotted line. In contrast, the mean primary growth of the independently RNAi transformed transgenic lines is represented by colored solid lines. The shaded bands around the smooth terms represent Bayesian Wabha/Silverman credible intervals. Month counts started in October 2018.

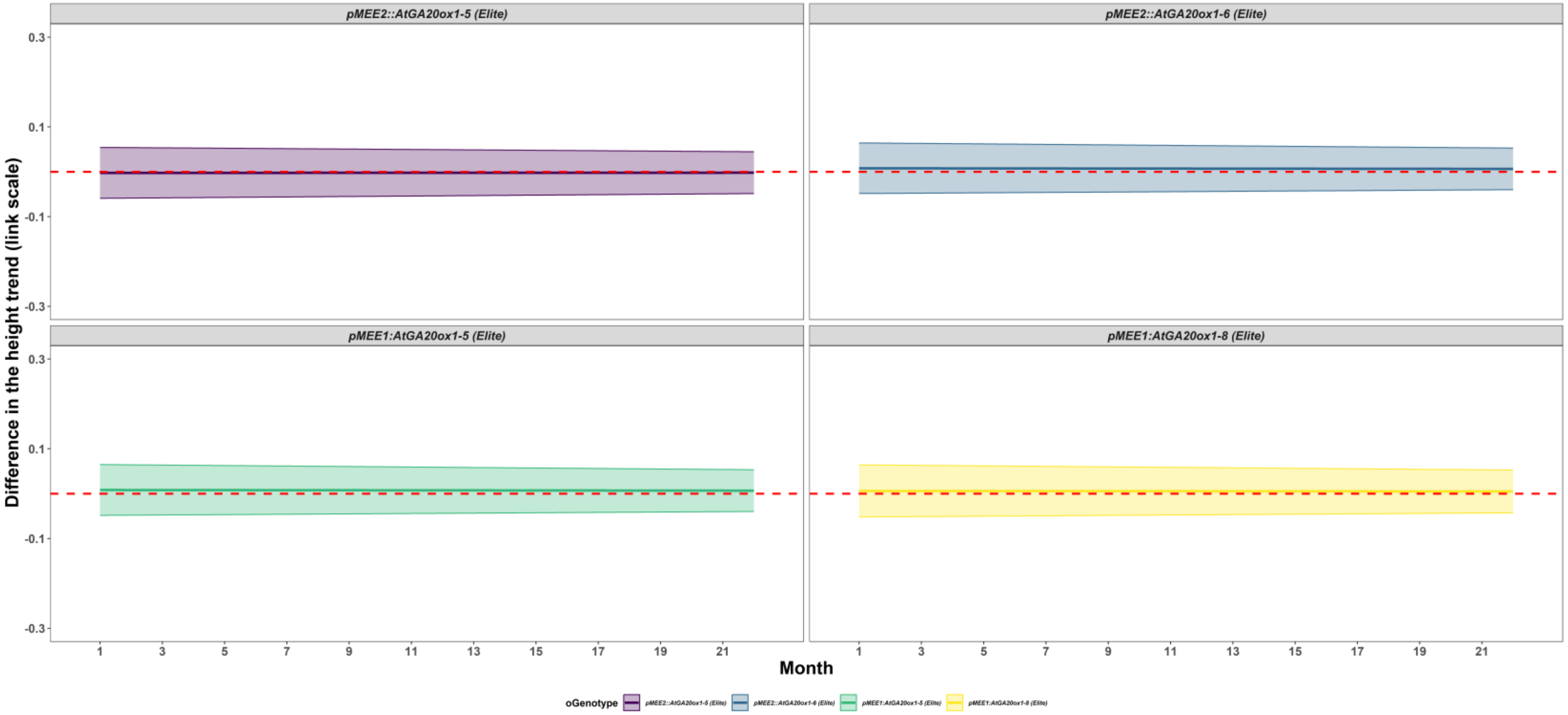

Figure S53: Term plots of the generalized additive (mixed) models with ordered-factor-smooth interaction smoothers modeling the mean primary growth of *P. tremula* L. x *P. tremuloides* trees during FE2. The mean primary growth of the reference WT^{Elite} is represented by the horizontal red dotted line. In contrast, the mean primary growth of the independently RNAi transformed transgenic lines is represented by colored solid lines. The shaded bands around the smooth terms represent Bayesian Wabha/Silverman credible intervals. Month counts started in October 2018.

Secondary growth

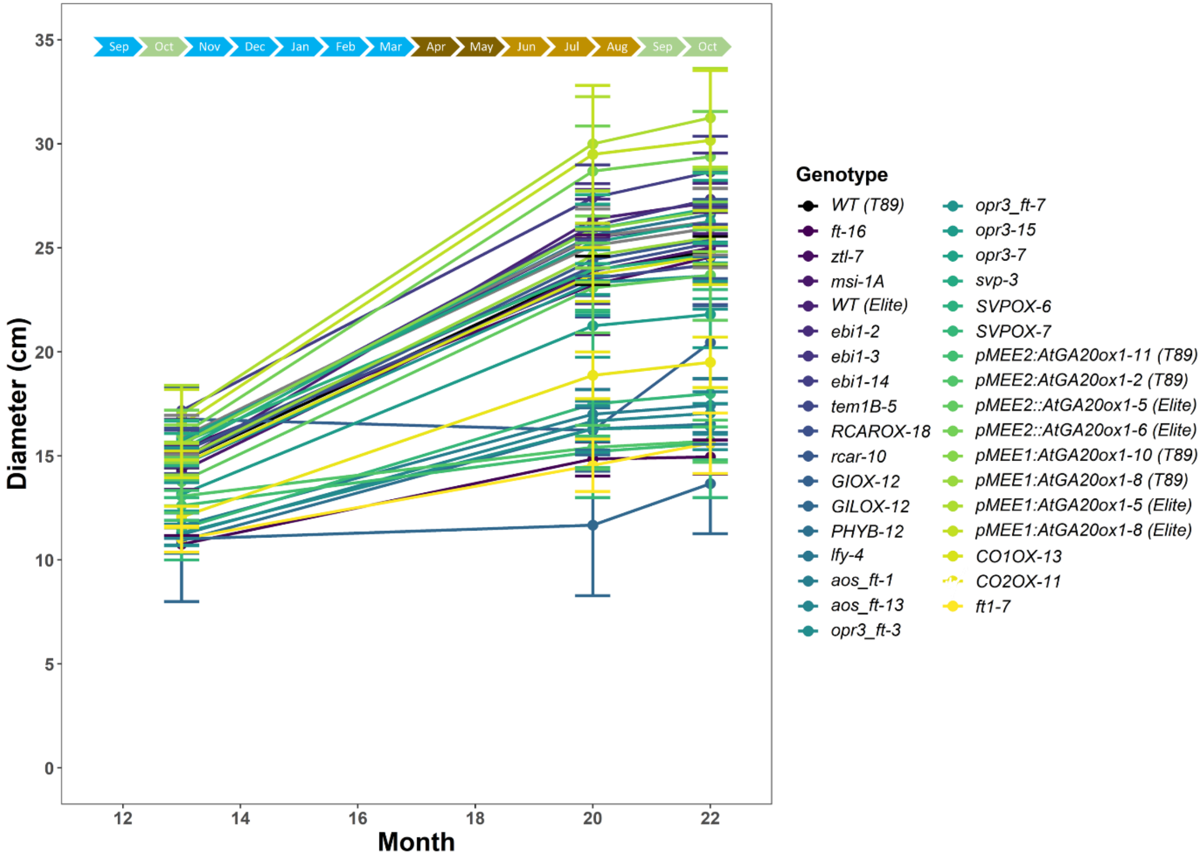

Figure S54.The secondary growth of *P. tremula* L. x *P. tremuloides* trees during FE2. The black solid line represents the secondary growth of the reference WT^{T89} while the colored solid lines represent the secondary growth of independently RNAi transformed transgenic lines. Dots and error bars represent mean and standard error values, respectively. Month counts started in October 2018.

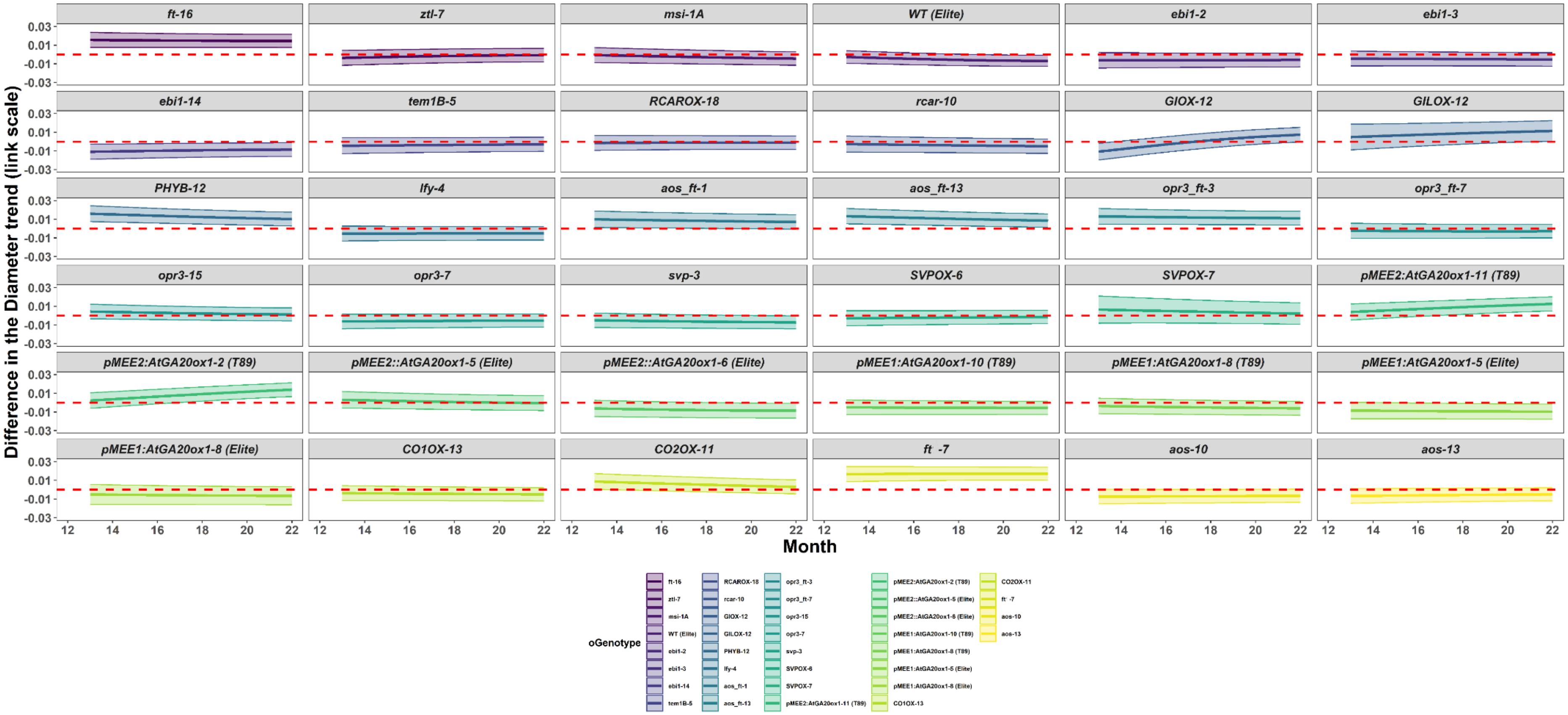

Figure S55. Term plots of the generalized additive (mixed) models with ordered-factor-smooth interaction smoothers modeling the mean secondary growth of *P. tremula* L. x *P. tremuloides* trees during FE2. The mean secondary growth of the reference WT^{T89} is represented by the horizontal red dotted line. In contrast, the mean secondary growth of the independently RNAi transformed transgenic lines is represented by colored solid lines. The shaded bands around the smooth terms represent Bayesian Wabha/Silverman credible intervals. Month counts started in October 2018.

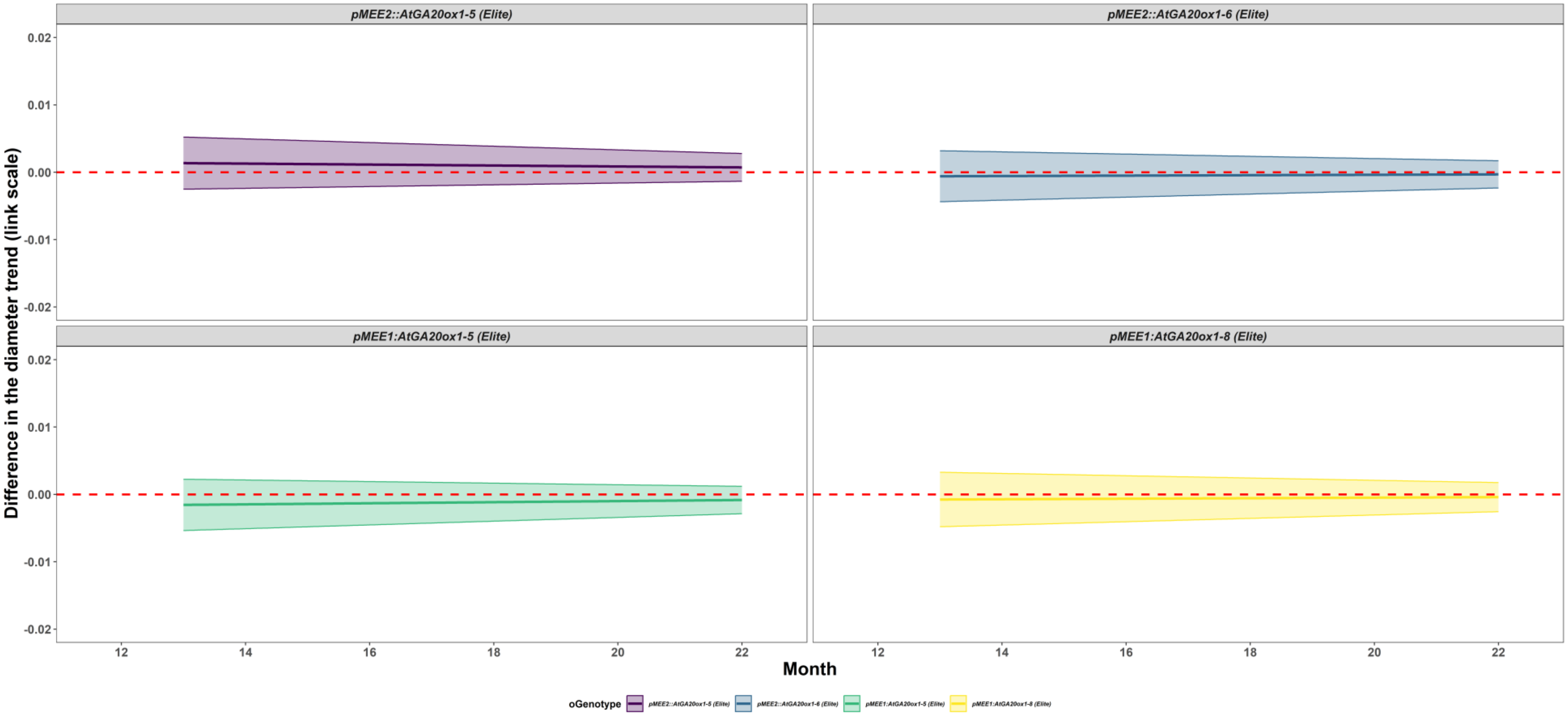

Figure S56. Term plots of the generalized additive (mixed) models with ordered-factor-smooth interaction smoothers modeling the mean secondary growth of *P. tremula* L. x *P. tremuloides* trees during FE2. The mean secondary growth of the reference WT^{Elite} is represented by the horizontal red dotted line. In contrast, the mean secondary growth of the independently RNAi transformed transgenic lines is represented by colored solid lines. The shaded bands around the smooth terms represent Bayesian Wabha/Silverman credible intervals. Month counts started in October 2018.

Quantitative reverse transcription polymerase chain reactions (RT-qPCR)

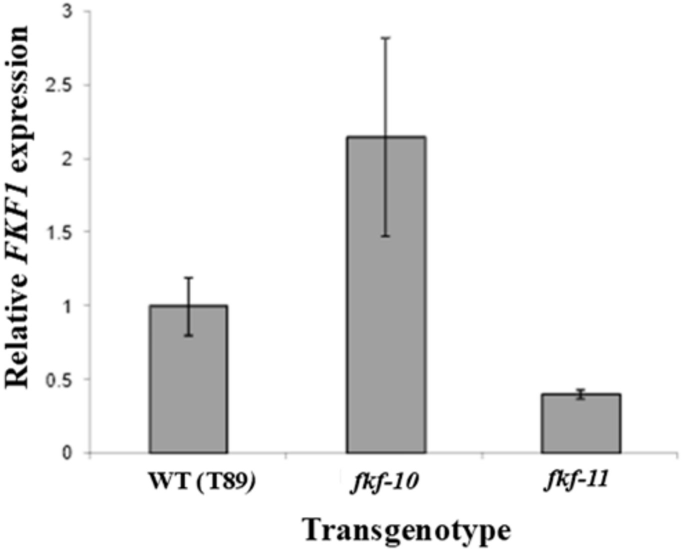

Figure S57. Relative expression of *FLAVIN-BINDING, KELCH REPEAT, F-BOX1* (*FKF1*) gene expression measured in samples collected at ZT 8 from WT^{T89} and *fkf* transgenotype trees. Bars and error plot represent mean and standard technical error values from leaf samples pooled from three to four biological replicates, respectively. Expression levels were normalized against expression of 18s rRNA with the ratio set to 1 in untreated WT^{T89}. Results are representative of repeated measures.

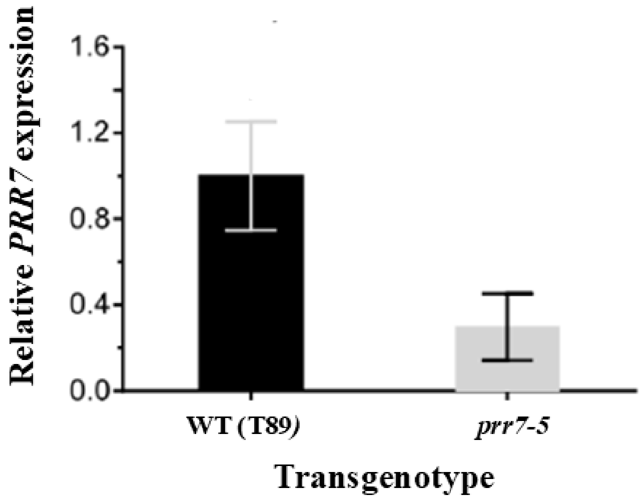

Figure S58. Relative expression of *PSEUDO-RESPONSE REGULATOR 7* (*PRR7*) gene expression measured in root samples collected at ZT 8 from WT^{T89} and *prr7* transgenotype trees. Bars and error plot represent mean and standard error values from three to four biological replicates, respectively. Expression levels were normalized against expression of *ELONGATION FACTOR 1 ALPHA* (*EF1a)* with the ratio set to 1 in untreated WT^{T89}. Data reproduced with permission from Ibanez *et al.* The *Populus* clock affects the regulation of gene expression and the metabolome in roots in response to ionic and osmotic stressors (unpublished).

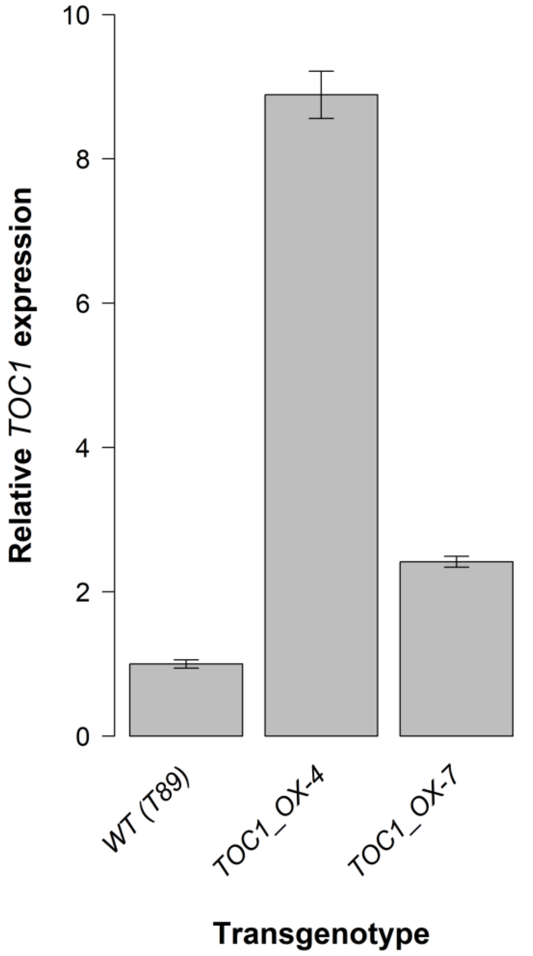

Figure S59. Relative ectopic *Populus nigra TIMING OF CAB EXPRESSION 1* (*TOC1*) gene expression, measured in samples collected at ZT 1 from WT^{T89} and *TOC1_OX* transgenotype trees. This represent a time point when TOC1 is not normally expressed in WT^{T89} . Bars and error plot represent mean and standard error values of three biological replicates, respectively. Expression levels were normalized against expression of *ELONGATION FACTOR 1 ALPHA* (*EF1a)* with the ratio set to 1 in untreated WT^{T89}.

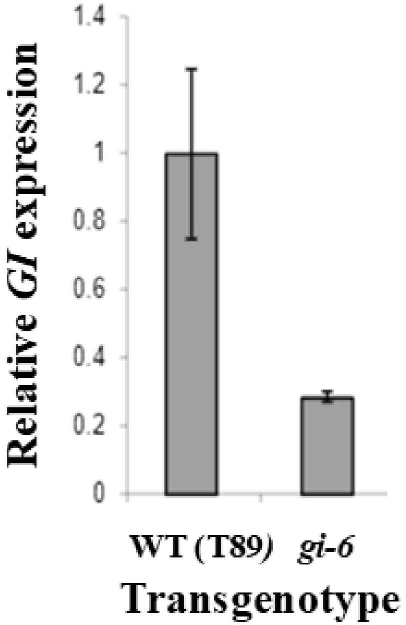

Figure S60. Relative expression of GIGANTEA (*GI*) gene expression measured in samples collected from leaves at ZT 8 from WT^{T89} and *gi* transgenotype trees. Bars and error plot represent mean and standard technical error values from pools of leaves from three to four biological replicates, respectively. Expression levels were normalized against expression of *ELONGATION FACTOR 1 ALPHA* (*EF1a)* with the ratio set to 1 in untreated WT^{T89}. Results are representative of repeated measures. Data reproduced with permission from Ibanez *et al.* The *Populus* clock affects the regulation of gene expression and the metabolome in roots in response to ionic and osmotic stressors (unpublished).

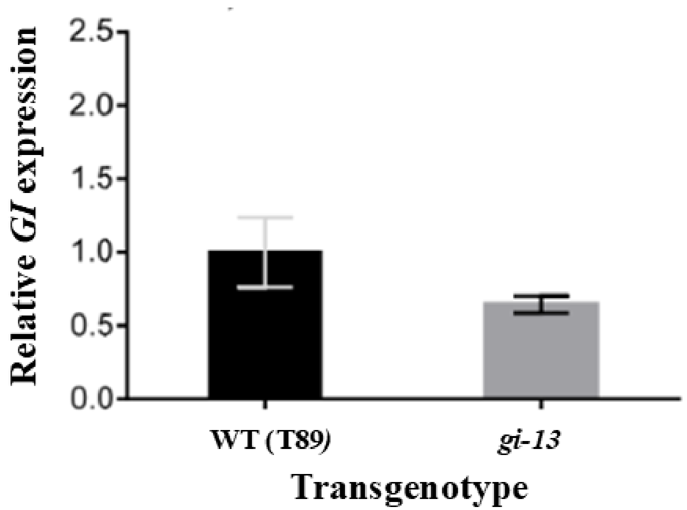

Figure S61. Relative expression of GIGANTEA (GI) gene expression measured in root samples collected at ZT 8 from WT^{T89} and *gi* transgenotype trees. Bars and error plot represent mean and standard error values from three to four biological replicates, respectively. Expression levels were normalized against expression of *ELONGATION FACTOR 1 ALPHA* (*EF1a)* with the ratio set to 1 in untreated WT^{T89}. Data reproduced with permission from Ibanez *et al.* The *Populus* clock affects the regulation of gene expression and the metabolome in roots in response to ionic and osmotic stressors (unpublished).

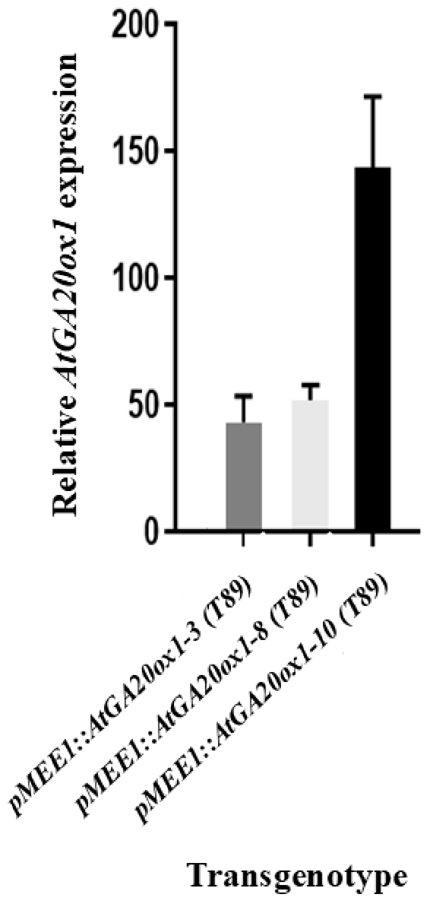

Figure S62. Relative expression of *GA20-OXIDASE1* gene expression measured in samples collected at ZT 8 from WT^{T89} and *pAIL1::AtGA20ox1* (T89) transgenotype trees. Bars and error plot represent mean and standard error values from three to four biological replicates, respectively. Expression levels were normalized against expression of *ELONGATION FACTOR 1 ALPHA* (*EF1a)* with the ratio set to 1 in untreated WT^{T89}.

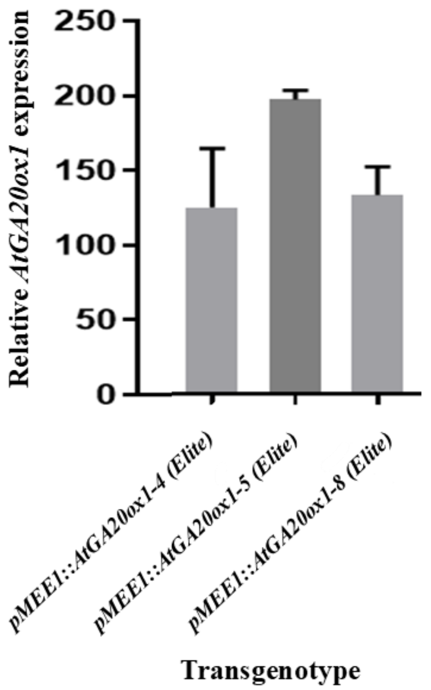

Figure S63. Relative expression of *GA20-OXIDASE1* gene expression measured in samples collected at ZT 8 from WT^{T89} and *pAIL1::AtGA20ox1* (Elite) transgenotype trees. Bars and error plot represent mean and standard error values from three to four biological replicates, respectively. Expression levels were normalized against expression of *ELONGATION FACTOR 1 ALPHA* (*EF1a)* with the ratio set to 1 in untreated WT^{T89}.

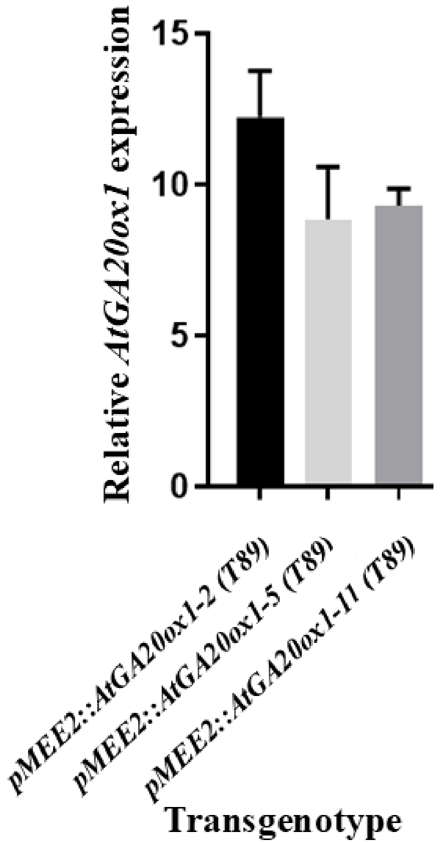

Figure S64. Relative expression of *GA20-OXIDASE1* gene expression measured in samples collected at ZT 8 from WT^{T89} and *pEL1.2::AtGA20ox1* (T89) transgenotype trees. Bars and error plot represent mean and standard error values, respectively. Expression levels were normalized against expression of *ELONGATION FACTOR 1 ALPHA* (*EF1a)* with the ratio set to 1 in untreated WT^{T89}.

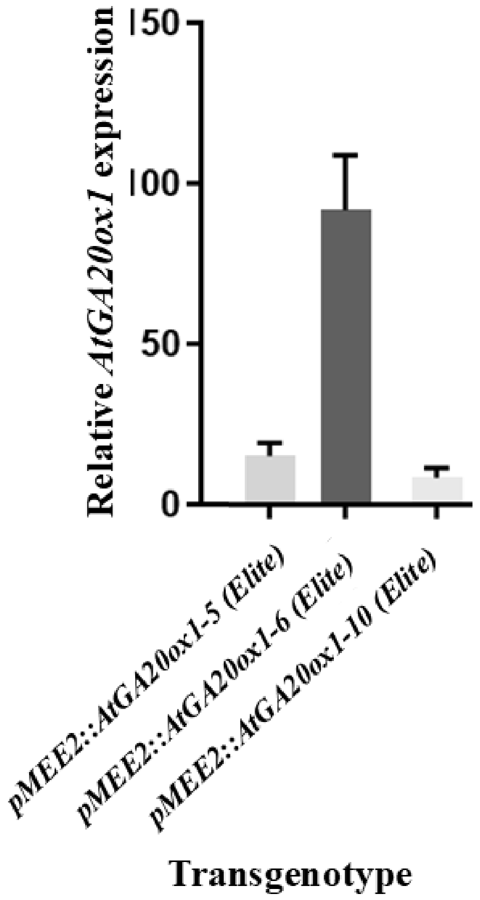

Figure S65. Relative expression of *GA20-OXIDASE1* gene expression measured in samples collected at ZT 8 from WT^{T89} and *pEL1.2::AtGA20ox1* (Elite) transgenotype trees. Bars and error plot represent mean and standard error values from three to four biological replicates, respectively. Expression levels were normalized against expression of *ELONGATION FACTOR 1 ALPHA* (*EF1a)* with the ratio set to 1 in untreated WT^{T89}.

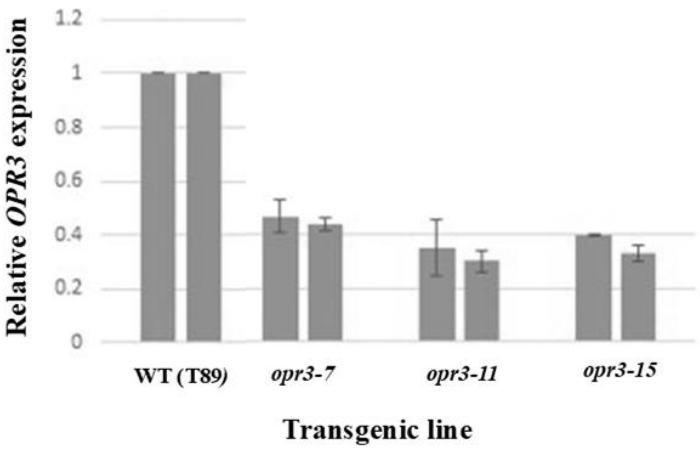

Figure S66. Relative expression of *12-OXOPHYTODIENOATE REDUCTASE 3* gene expression measured in samples collected from leaves at ZT 8 from WT^{T89} and *opr3* transgenotype trees. Bars and error plot represent mean and standard error values from three to four biological replicates, respectively. Expression levels were normalized against expression of *ELONGATION FACTOR 1 ALPHA* (*EF1a)* with the ratio set to 1 in untreated WT^{T89}.

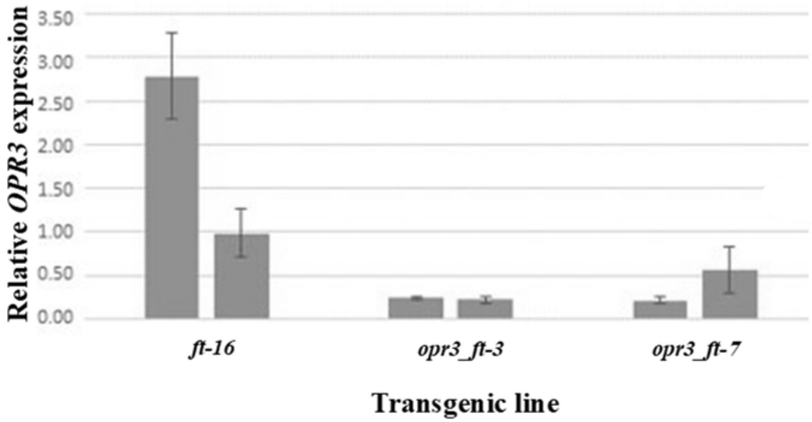

Figure S67. Relative expression of *12-OXOPHYTODIENOATE REDUCTASE 3* gene expression measured in samples in leaves collected at ZT 8 from WT^{T89} and *opr3_ft* transgenotype trees. Bars and error plot represent mean and standard error values from three to four biological replicates, respectively. Expression levels were normalized against expression of *ELONGATION FACTOR 1 ALPHA* (*EF1a)* with the ratio set to 1 in untreated *ft-16*.

Supplemental Tables

Table S1. The growth and phenology behavior of *Populus sp.* circadian clock mutant lines in contrast to WT^{T89}. The name stands for either an experiment done in growth chambers (GCE), the phenotyping platform (PPE) or the field (FE). ID and Ref. stands for identity and reference line, while Ht., Diam., CCI, Bs. and Bb. stand for the height, diameter, chlorophyll content index, bud set and bud burst, respectively. Measures of significance are symbolized as + and – for positive and negative significant differences between the growth or phenology of a circadian clock mutant and WT^{T89} (i.e., clock mutants with + or – grow significantly faster or slower, respectively. Likewise, clock mutants with D or A display a significant delay or advance, than WT^{T89}, respectively). Non-significant results are abbreviated as ns. In other words, + and – represent here significant delays and advancements in the according phenological event compared to the occurrence of the same phenological event in WT^{T89}. n/a stands for not available. The colors facilitate the comparison of results with +, - being highlighted by red, green, and yellow cell colors.

Transgenotype	ID	Line	GCE1		PPE		FE1				GCE2	GCE3				GCE4					GCE5	FE2	
			Ht.	Diam.	Ht.	Diam.	Ht.	Diam.	Bs.	CCI	Ht.	Ht.	Bs.	Bb. (ap)	Bb. (lat)	Ht.	Diam.	Bs.	Bb. (ap)	Bb. (lat)	Ht.	Ht.	Diam.
aos_ft-1	588	1	n/a	n/a	n/a	n/a	n/a	n/a	n/a	n/a	n/a	ns	A	ns	ns	n/a	n/a	n/a	n/a	n/a	n/a	-	+
aos_ft-10	588	10	n/a	n/a	n/a	n/a	n/a	n/a	n/a	n/a	n/a	+	A	ns	D	n/a	n/a	n/a	n/a	n/a	n/a	n/a	n/a
aos_ft-13	588	13	n/a	n/a	n/a	n/a	n/a	n/a	n/a	n/a	n/a	-	A	ns	D	n/a	n/a	n/a	n/a	n/a	n/a	-	+
aos-1	589	1	n/a	n/a	n/a	n/a	n/a	n/a	n/a	n/a	n/a	ns	D	ns	ns	n/a	n/a	n/a	n/a	n/a	n/a	n/a	n/a
aos-10	589	10	n/a	n/a	ns	ns	n/a	n/a	n/a	n/a	n/a	ns	D	ns	ns	n/a	n/a	n/a	n/a	n/a	n/a	+	ns
aos-13	589	13	n/a	n/a	ns	+	n/a	n/a	n/a	n/a	n/a	ns	D	D	ns	n/a	n/a	n/a	n/a	n/a	n/a	+	ns
CO1OX-13	688	13	n/a	n/a	n/a	n/a	n/a	n/a	n/a	n/a	n/a	n/a	n/a	n/a	n/a	n/a	n/a	n/a	n/a	n/a	n/a	+	ns
CO2OX-11	689	11	n/a	n/a	n/a	n/a	n/a	n/a	n/a	n/a	n/a	n/a	n/a	n/a	n/a	n/a	n/a	n/a	n/a	n/a	n/a	-	ns
ebi1-14	345	14	+	ns	ns	ns	+	+	ns	ns	n/a	n/a	n/a	n/a	n/a	n/a	n/a	n/a	n/a	n/a	n/a	+	-
ebi1-2	312	2	+	+	+	+	+	+	ns	ns	n/a	n/a	n/a	n/a	n/a	n/a	n/a	n/a	n/a	n/a	n/a	+	ns
ebi1-3	312	3	+	+	ns	ns	+	+	ns	+	n/a	n/a	n/a	n/a	n/a	n/a	n/a	n/a	n/a	n/a	n/a	+	ns
ebi2-6	313	6	n/a	n/a	n/a	n/a	+	+	ns	ns	n/a	n/a	n/a	n/a	n/a	n/a	n/a	n/a	n/a	n/a	n/a	n/a	n/a
ebi2-7	313	7	n/a	n/a	-	-	-	ns	A	ns	n/a	n/a	n/a	n/a	n/a	n/a	n/a	n/a	n/a	n/a	n/a	n/a	n/a
ebi2-8	313	8	n/a	n/a	-	-	ns	ns	A	ns	n/a	n/a	n/a	n/a	n/a	n/a	n/a	n/a	n/a	n/a	n/a	n/a	n/a
fkf1-10	124	10	n/a	n/a	ns	ns	n/a	n/a	n/a	n/a	n/a	n/a	n/a	n/a	n/a	n/a	n/a	n/a	n/a	n/a	n/a	n/a	n/a
fkf1-11	124	11	n/a	n/a	ns	ns	-	-	A	-	n/a	n/a	n/a	n/a	n/a	n/a	n/a	n/a	n/a	n/a	n/a	n/a	n/a
ft-16	1044	16	n/a	n/a	n/a	n/a	-	-	A	-	-	+	A	D	ns	n/a	n/a	n/a	n/a	n/a	n/a	-	+
ft-7	707	1	n/a	n/a	n/a	n/a	n/a	n/a	n/a	n/a	n/a	n/a	n/a	n/a	n/a	n/a	n/a	n/a	n/a	n/a	n/a	-	+
gi-13	125	13	ns	ns	ns	-	+	ns	D	+	n/a	n/a	n/a	n/a	n/a	n/a	n/a	n/a	n/a	n/a	n/a	n/a	n/a
gi-6	125	6	n/a	n/a	-	ns	n/a	n/a	n/a	n/a	n/a	n/a	n/a	n/a	n/a	n/a	n/a	n/a	n/a	n/a	n/a	n/a	n/a
GILOX-12	532	12	n/a	n/a	n/a	n/a	n/a	n/a	n/a	n/a	n/a	n/a	n/a	n/a	n/a	n/a	n/a	n/a	n/a	n/a	n/a	-	ns
GIOX-12	523	12	n/a	n/a	n/a	n/a	n/a	n/a	n/a	n/a	n/a	n/a	n/a	n/a	n/a	n/a	n/a	n/a	n/a	n/a	n/a	-	ns
lfy-4	581	4	n/a	n/a	n/a	n/a	n/a	n/a	n/a	n/a	n/a	n/a	n/a	n/a	n/a	n/a	n/a	n/a	n/a	n/a	n/a	+	ns
lhy-10	3	10	-	-	-	ns	+	+	D	ns	n/a	n/a	n/a	n/a	n/a	n/a	n/a	n/a	n/a	n/a	n/a	n/a	n/a
msi-1A	26	1A	n/a	n/a	n/a	n/a	n/a	n/a	n/a	n/a	n/a	n/a	n/a	n/a	n/a	n/a	n/a	n/a	n/a	n/a	n/a	ns	ns
opr3_ft-3	590	3	n/a	n/a	n/a	n/a	n/a	n/a	n/a	n/a	ns	n/a	n/a	n/a	n/a	n/a	n/a	n/a	n/a	n/a	n/a	-	+
opr3_ft-7	590	7	n/a	n/a	ns	ns	n/a	n/a	n/a	n/a	ns	n/a	n/a	n/a	n/a	n/a	n/a	n/a	n/a	n/a	n/a	ns	ns
opr3_ft-12	590	12	n/a	n/a	n/a	n/a	n/a	n/a	n/a	n/a	-	n/a	n/a	n/a	n/a	n/a	n/a	n/a	n/a	n/a	n/a	n/a	n/a
opr3-11	591	11	n/a	n/a	n/a	n/a	n/a	n/a	n/a	n/a	ns	n/a	n/a	n/a	n/a	n/a	n/a	n/a	n/a	n/a	n/a	n/a	n/a
opr3-15	591	15	n/a	n/a	+	ns	n/a	n/a	n/a	n/a	+	n/a	n/a	n/a	n/a	n/a	n/a	n/a	n/a	n/a	n/a	ns	ns

opr3-7	591	7	n/a	n/a	n/a	n/a	n/a	n/a	n/a	n/a	ns	n/a	n/a	n/a	n/a	n/a	n/a	n/a	n/a	n/a	n/a	ns	ns
pAtCCR2::LUC PHYA1-1	128	1	n/a	n/a	n/a	n/a	-	-	ns	ns	n/a	n/a	n/a	n/a	n/a	n/a	n/a	n/a	n/a	n/a	n/a	n/a	n/a
pAtCCR2::LUC PHYA22-2	134	2	n/a	n/a	-	-	-	+	D	+	n/a	n/a	n/a	n/a	n/a	n/a	n/a	n/a	n/a	n/a	n/a	n/a	n/a
pAtCCR2::LUC T89-5	127	5	n/a	n/a	ns	ns	n/a	n/a	n/a	n/a	n/a	n/a	n/a	n/a	n/a	n/a	n/a	n/a	n/a	n/a	n/a	n/a	n/a
PHYB-12	537	12	n/a	n/a	+	ns	n/a	n/a	n/a	n/a	n/a	n/a	n/a	n/a	n/a	n/a	n/a	n/a	n/a	n/a	n/a	-	+
PHYB2KO-6	848	6	n/a	n/a	+	ns	n/a	n/a	n/a	n/a	n/a	n/a	n/a	n/a	n/a	n/a	n/a	n/a	n/a	n/a	n/a	n/a	n/a
pMEE1::AtGA20ox1-10 (T89)	640	10	n/a	n/a	n/a	n/a	n/a	n/a	n/a	n/a	n/a	n/a	n/a	n/a	n/a	-	ns	D	A	A	n/a	+	ns
pMEE1::AtGA20ox1-8 (T89)	640	8	n/a	n/a	ns	ns	n/a	n/a	n/a	n/a	n/a	n/a	n/a	n/a	n/a	ns	ns	D	A	A	n/a	ns	ns
pMEE1::AtGA20ox1-3 (T89)	640	3	n/a	n/a	n/a	n/a	n/a	n/a	n/a	n/a	n/a	n/a	n/a	n/a	n/a	ns	ns	D	A	A	n/a	n/a	n/a
pMEE1::AtGA20ox1-4 (Elite)	641	4	n/a	n/a	n/a	n/a	n/a	n/a	n/a	n/a	n/a	n/a	n/a	n/a	n/a	ns	ns	A	D	D	n/a	n/a	n/a
pMEE1::AtGA20ox1-5 (Elite)	641	5	n/a	n/a	n/a	n/a	n/a	n/a	n/a	n/a	n/a	n/a	n/a	n/a	n/a	ns	-	A	D	D	n/a	+	-
pMEE1::AtGA20ox1-8 (Elite)	641	8	n/a	n/a	n/a	n/a	n/a	n/a	n/a	n/a	n/a	n/a	n/a	n/a	n/a	+	+	A	D	D	n/a	ns	ns
pMEE2::AtGA20ox1-10 (Elite)	638	10	n/a	n/a	n/a	n/a	n/a	n/a	n/a	n/a	n/a	n/a	n/a	n/a	n/a	ns	ns	A	D	D	n/a	n/a	n/a
pMEE2::AtGA20ox1-5 (Elite)	638	5	n/a	n/a	n/a	n/a	n/a	n/a	n/a	n/a	n/a	n/a	n/a	n/a	n/a	+	+	A	D	D	n/a	ns	ns
pMEE2::AtGA20ox1-6 (Elite)	638	6	n/a	n/a	n/a	n/a	n/a	n/a	n/a	n/a	n/a	n/a	n/a	n/a	n/a	+	+	A	D	D	n/a	ns	ns
pMEE2::AtGA20ox1-11 (T89)	637	11	n/a	n/a	n/a	n/a	n/a	n/a	n/a	n/a	n/a	n/a	n/a	n/a	n/a	-	ns	D	A	A	n/a	+	+
pMEE2::AtGA20ox1-2 (T89)	637	2	n/a	n/a	+	+	n/a	n/a	n/a	n/a	n/a	n/a	n/a	n/a	n/a	-	-	D	n/a	A	n/a	+	+
pMEE2::AtGA20ox1-5 (T89)	637	5	n/a	n/a	n/a	n/a	n/a	n/a	n/a	n/a	n/a	n/a	n/a	n/a	n/a	-	ns	D	n/a	A	n/a	n/a	n/a
prr7-5	261	5	ns	ns	n/a	n/a	ns	ns	ns	ns	n/a	n/a	n/a	n/a	n/a	n/a	n/a	n/a	n/a	n/a	n/a	n/a	n/a
rcar-10	474	10	n/a	n/a	n/a	n/a	n/a	n/a	n/a	n/a	n/a	n/a	n/a	n/a	n/a	n/a	n/a	n/a	n/a	n/a	n/a	+	ns
RCAROX-18	473	18	n/a	n/a	n/a	n/a	n/a	n/a	n/a	n/a	n/a	n/a	n/a	n/a	n/a	n/a	n/a	n/a	n/a	n/a	n/a	+	ns
svp-3	603	3	n/a	n/a	n/a	n/a	n/a	n/a	n/a	n/a	n/a	n/a	n/a	n/a	n/a	n/a	n/a	n/a	n/a	n/a	n/a	+	ns
SVPOX-6	624	6	n/a	n/a	n/a	n/a	n/a	n/a	n/a	n/a	n/a	n/a	n/a	n/a	n/a	n/a	n/a	n/a	n/a	n/a	n/a	+	ns
SVPOX-7	624	7	n/a	n/a	n/a	n/a	n/a	n/a	n/a	n/a	n/a	n/a	n/a	n/a	n/a	n/a	n/a	n/a	n/a	n/a	n/a	-	ns
tem1B-5	365	5	n/a	n/a	n/a	n/a	n/a	n/a	n/a	n/a	n/a	n/a	n/a	n/a	n/a	n/a	n/a	n/a	n/a	n/a	n/a	ns	ns
TOC1_OX-4	1119	4	n/a	n/a	ns	ns	n/a	n/a	ns	ns	n/a	n/a	n/a	n/a	n/a	n/a	n/a	n/a	n/a	n/a	n/a	n/a	n/a
TOC1_OX-7	1119	7	n/a	n/a	ns	ns	+	ns	n/a	n/a	n/a	n/a	n/a	n/a	n/a	n/a	n/a	n/a	n/a	n/a	n/a	n/a	n/a
toc1-5	1	5	n/a	n/a	ns	ns	+	+	ns	ns	n/a	n/a	n/a	n/a	n/a	n/a	n/a	n/a	n/a	n/a	n/a	n/a	n/a
WT (Elite)	3011	864012	n/a	n/a	+	+	n/a	n/a	n/a	n/a	n/a	n/a	n/a	n/a	n/a	+	ns	A	D	D	ns	ns	ns
WT (Elite-884056)	3011	884056	n/a	n/a	n/a	n/a	n/a	n/a	n/a	n/a	n/a	n/a	n/a	n/a	n/a	n/a	n/a	n/a	n/a	n/a	ns	n/a	n/a
WT (T89)	n/a	T89	Ref.	Ref.	Ref.	Ref.	Ref.	Ref.	Ref.	Ref.	Ref.	Ref.	Ref.	Ref.	Ref.	Ref.	Ref.	Ref.	Ref.	Ref.	Ref.	Ref.	Ref.
ztl-3	11	3	n/a	n/a	n/a	n/a	ns	ns	ns	ns	n/a	n/a	n/a	n/a	n/a	n/a	n/a	n/a	n/a	n/a	n/a	n/a	n/a
ztl-5	11	5	-	-	ns	ns	ns	ns	ns	-	n/a	n/a	n/a	n/a	n/a	n/a	n/a	n/a	n/a	n/a	n/a	n/a	n/a
ztl-7	11	7	ns	ns	n/a	n/a	+	ns	ns	+	n/a	n/a	n/a	n/a	n/a	n/a	n/a	n/a	n/a	n/a	n/a	ns	ns

Table S2: Overview of the introduced transgenotypes. Information is provided on important construct aspects (i.e., the binary vector, length of the base pairs target sequences, and gene models in relevant species with *Populus trichocarpa v.4* genome, https://phytozome-next.jgi.doe.gov/, as general *Populus sp.* reference). Detailed information (e.g., RNAi, promoter:gene construct or overexpressor) is provided below. Sequences of the included targeted gene fragment are indicated in forward (fwd) and reverse (rev) orientation, respectively. Underlined sequences refer to specific vector sequences.

Transgenotype	*Binary vector*	*Gene(s)*	*Base pairs*	*Gene model*	*Construct target sequences*
aos<br>*aos-10*<br>*aos-13*<br>*aos-1*	pGWB502	*AOS*	20	Potri.014G038700,<br>Potri.002G130700	5'-AGCAAAGCGTGATCTGATAA-3'
aos_ft<br>*aos_ft-1*<br>*aos_ft-13*<br>*aos_ft-10*	pK7GWIWG2	*AOS/ FT1, 2*	230	Potri.014G038700,<br>Potri.002G130700,<br>Potri.008G077700<br>Potri.010G179700	5'-AGCAAAGCGTGATCTGATAA-3'<br>in line 16c RNAi *FT1* & *2*[5]
ebi1<br>*ebi1-2*<br>*ebi1-3*<br>*ebi1-14*	pANDA35HK	*EBI1a, b*	354	Potri.008G068500,<br>Potri.010G188700	5'-CACCGCGGCCGCGGACTTGGACTTCTTCCT-3' (fwd),<br>5'-GATTCGTGGATGTCTTCTTCTGTG-3' (rev)
ebi2<br>*ebi2-6*<br>*ebi2-7*<br>*ebi2-8*	pANDA35HK	*EBI2a, b*	399	Potri.012G043700,<br>Potri.015G034500	5'-CACCGCGGCCGCCCATCTCGTGTGATTGGC-3' (fwd),<br>5'-CTTCCACGAAGTTCCCTTCAGAG-3' (rev)
fkf1<br>*fkf1-10*<br>*fkf1-11*	pHELLSGATE8	*FKF1, 2*	354	Potri.010G105700,<br>Potri.008G135200	5'-GGGGACAAGTTTGTACAAAAAAGCAGGC<br>ATGAGCAGGTAAGGAAGATGG-3' (fwd),<br>5'-GGGGACCACTTTGTACAAGAAAGCTGGGT<br>ACAACACCAACCAGGAACC-3' (rev)
**GI**<br>*gi-6*<br>gi-13	pHELLSGATE8	*GI1, 2*	375	Potri.005G196700,<br>Potri.002G064400	5'-GGGGACAAGTTTGTACAAAAAAGCAGGC-3' (fwd),<br>5'-GGGGACCACTTTGTACAAGAAAGCTGGGT-3' (rev)
opr3<br>*opr3-7,*<br>*opr3-11,*<br>*opr3-15*	pGWB502	*OPR3*	20	Potri.006G142800	5'-CAGCCACGGTACACAGCTTA-3'
opr3_ft<br>*opr3_ft-3*<br>*opr3_ft-7*<br>*opr3_ft-12*	pK7GWIWG2	*OPR3/ FT1, 2*	230	Potri.006G142800,<br>Potri.008G077700<br>Potri.010G179700	5'-CAGCCACGGTACACAGCTTA-3'<br>in line 16c RNAi *FT1* & *2*[5]
pAIL1::AtGA20ox1<br>*pMEE1::AtGA20ox1-8 (T89)*<br>*pMEE1::AtGA20ox1-10 (T89)*<br>*pMEE1::AtGA20ox1-3 (T89)*	pK2GW7	*Promoter AIL1, AtGA20ox1*	2683,<br>1281	Potri.002G114800[6]	5'-GCAGAGCTCGGGGAATGATAGGCTGACAAG-3' (*Sac I*)<br>5'-GCAACTAGTCCCAAAATCTTGCCTACTTCCAT-3' (Spe *I*)<br>driving *AtGA20ox1* expression[7]
pMEE1::AtGA20ox1-5 (Elite)<br>*pMEE1::AtGA20ox1-8 (Elite)*<br>*pMEE1::AtGA20ox1-4 (Elite)*	pK2GW7	*Promoter AIL1, AtGA20ox1*	2683,<br>281	Potri.002G114800[6]	5'-GCAGAGCTCGGGGAATGATAGGCTGACAAG-3' (*Sac I*)<br>5'-GCAACTAGTCCCAAAATCTTGCCTACTTCCAT-3' (Spe *I*)<br>driving *AtGA20ox1* expression[7]
pEL1.2::AtGA20ox1<br>*pMEE2:AtGA20ox1-2 (T89)*<br>*pMEE2:AtGA20ox1-11 (T89)*<br>*pMEE2:AtGA20ox1-5 (T89)*<br>*pMEE2:AtGA20ox1-6 (Elite)*	pK2GW7	*Promoter EL1.2, AtGA20ox1*	1800,<br>1281	*Eucalyptus globulus*<br>Eucgr.B03013.1<br>EgRBCS3	See Jonsen, et al. [8] and Suzuki, et al. [9]
pMEE2::AtGA20ox1-5 (Elite)<br>*pMEE2::AtGA20ox1-10 (Elite)*	pK2GW7	*Promoter EL1.2, AtGA20ox1*	1800,<br>1281	*Eucalyptus globulus*<br>*Eucgr.B03013.1*<br>EgRBCS3	See Jonsen, et al. [8] and Suzuki, et al. [9]
prr7<br>*prr7-5*	pK7GWIWG2	*PRR7a, b*	420	Potri.008G046200<br>Potri.010G215200	5'-CAATGACATGGGCTCTACAAATAATATTAC-3' (fwd),<br>5'-GGAAGGCAGAAGGATATTGGCT-3' (rev)
TOC1_OX<br>*TOC1_OX-4,*<br>*TOC1_OX-7*	pGWB2	*TOC1*	1600	*Populus nigra*<br>Potri.015G061900	5'-ATATCGACCTGATGTGCCAGGGAT -3' (fwd)<br>5'-GCTCGCCACATACTTGAAGTTTCTCT -3' (rev)

Table S3. Overview of transgenotypes occurring in the literature. Information is provided in relation to *Populus* (P) and *Arabidopsis* (A). n/a stands for not available.

Genotype	Transgenotypes	Ref. (P)	Ref. (A)
CO1OX	*CO1OX-13*	10,11	5,12
CO2OX	*CO2OX-11*	10,11	5,12
ebi1	*ebi1-2, ebi1-3, ebi1-14*	13	14,15
ebi2	*ebi2-6, ebi2-7, ebi2-8*	13	14,15
ft	*ft-16, ft-7*	3,5,16	n/a
GILOX	*GILOX-12*	12,13	n/a
GIOX	*GIOX-12*	12	n/a
lfy	*lfy-4*	17,18	17,18
lhy	*lhy-10*	2,19	n/a
msi	*msi-1A*	20	n/a
pAtCCR2::LUC PHYA1	*pAtCCR2::LUC PHYA1-1*	21-23	n/a
pAtCCR2::LUC PHYA22	*pAtCCR2::LUC PHYA22-2*	21,22	n/a
pAtCCR2::LUC T89	*pAtCCR2::LUC T89-5*	2,21,23	n/a
phyB	*PHYB-12*		
PHYB2KO	*PHYB2KO-6*	24	n/a
rcar	*rcar-10*	25	n/a
RCAROX	*RCAROX-18*	25	n/a
svp	*svp-3*	16	26,27
SVPOX	*SVPOX-6, SVPOX-7*	16	1,248
tem1B	*tem1B-5*	11,28	n/a
toc1	*toc1-4, toc1-, toc1-7*	2,19	29,30
WT	WT (T89)	22	n/a
WT (Elite)	WT (Elite-864012)	31	n/a
WT (Elite-884056)	WT (Elite-884056)	31	n/a
ztl	*ztl-3, ztl-5, ztl-7*	7	n/a

References

1 UPOV. Guidelines for the Conduct of Test for Distinctiveness, Uniformity and Stability Populus L., 9 (International Union For The Protection Of New Varieties Of Plants, Geneva, 1981).

2 Ibanez, C. *et al.* Circadian clock components regulate entry and affect exit of seasonal dormancy as well as winter hardiness in Populus trees. *Plant Physiology* **153**, 1823-1833 (2010). https://doi.org/10.1104/pp.110.158220

3 Wang, J. *et al.* A major locus controls local adaptation and adaptive life history variation in a perennial plant. *Genome Biology* **19**, 72 (2018). https://doi.org/10.1186/s13059-018-1444-y

4 Robinson, K. M. *et al.* Variation in non-target traits in genetically modified hybrid aspens does not exceed natural variation. *New Biotechnology* **64**, 27-36 (2021). https://doi.org/10.1016/j.nbt.2021.05.005

5 Böhlenius, H. *et al.* CO/FT Regulatory Module Controls Timing of Flowering and Seasonal Growth Cessation in Trees. *Science* **312**, 1040-1043 (2006). https://doi.org/10.1126/science.1126038

6 Rigal, A. *et al.* The AINTEGUMENTA LIKE1 homeotic transcription factor PtAIL1 controls the formation of adventitious root primordia in poplar. *Plant Physiology* **160**, 1996-2006 (2012). https://doi.org/10.1104/pp.112.204453

7 Jurca, M. *et al.* ZEITLUPE Promotes ABA-Induced Stomatal Closure in Arabidopsis and Populus. *Frontiers in Plant Science* **13** (2022). https://doi.org/10.3389/fpls.2022.829121

8 Jonsen, D. *et al.* Plants with improved growth. Sweden patent (2018).

9 Suzuki, Y. *et al.* Differences in Rubisco content and its synthesis in leaves at different positions in Eucalyptus globulus seedlings. *Plant, Cell & Environment* **33**, 1314-1323 (2010). https://doi.org/10.1111/j.1365-3040.2010.02149.x

10 Hsu, C. Y. *et al.* Overexpression of CONSTANS homologs CO1 and CO2 fails to alter normal reproductive onset and fall bud set in woody perennial poplar. *PloS One* **7**, e45448 (2012). https://doi.org/10.1371/journal.pone.0045448

11 Triozzi, P. M. *et al.* Photoperiodic Regulation of Shoot Apical Growth in Poplar. *Frontiers in Plant Science* **9** (2018). https://doi.org/10.3389/fpls.2018.01030

12 Ding, J. *et al.* GIGANTEA-like genes control seasonal growth cessation in Populus. *New Phytologist* **218**, 1491-1503 (2018). https://doi.org/10.1111/nph.15087

13 Eriksson, M. E. *et al.* Plants with improved growth characteristics. (2015).

14 Ashelford, K. *et al.* Full genome re-sequencing reveals a novel circadian clock mutation in Arabidopsis. *Genome biology* **12**, R28 (2011). https://doi.org/10.1186/gb-2011-12-3-r28

15 Johansson, M. *et al.* Partners in time: EARLY BIRD associates with ZEITLUPE and regulates the speed of the Arabidopsis clock. *Plant Physiology* **155**, 2108-2122 (2011). https://doi.org/10.1104/pp.110.167155

16 Andre, D. *et al.* FLOWERING LOCUS T paralogs control the annual growth cycle in Populus trees. *Current Biology* **32**, 2988-2996 e2984 (2022). https://doi.org/10.1016/j.cub.2022.05.023

17 Weigel, D. *et al.* A developmental switch sufficient for flower initiation in diverse plants. *Nature* **377**, 495-500 (1995). https://doi.org/10.1038/377495a0

18 Böhlenius, H.

19 Edwards, K. D. *et al.* Circadian clock components control daily growth activities by modulating cytokinin levels and cell division-associated gene expression in Populus trees. *Plant, Cell & Environment* **41**, 1468-1482 (2018). https://doi.org/10.1111/pce.13185

20 Englund, M. *Molecular control of activity–dormancy transitions in Populus* PhD thesis, Swedish University of Agricultural Sciences, (2010).

21 Nilsson, O. *et al.* Spatial pattern of cauliflower mosaic virus 35S promoter-luciferase expression in transgenic hybrid aspen trees monitored by enzymatic assay and non-destructive imaging. *Transgenic Research* **1**, 209-220 (1992). https://doi.org/10.1007/BF02524751

22 Olsen, J. E. *et al.* Ectopic expression of oat phytochrome A in hybrid aspen changes critical daylength for growth and prevents cold acclimatization. *The Plant Journal* **12**, 1339-1350 (1997). https://doi.org/10.1046/j.1365-313x.1997.12061339.x
23 Kozarewa, I. *et al.* Alteration of PHYA expression change circadian rhythms and timing of bud set in Populus. *Plant Molecular Biology* **73**, 143-156 (2010). https://doi.org/10.1007/s11103-010-9619-2
24 Ding, J. *et al.* Phytochrome B and PHYTOCHROME INTERACTING FACTOR8 modulate seasonal growth in trees. *New Phytologist* **232**, 2339-2352 (2021). https://doi.org/10.1111/nph.17350
25 Singh, R. K. *et al.* A genetic network mediating the control of bud break in hybrid aspen. *Nature Communications* **9**, 4173 (2018). https://doi.org/10.1038/s41467-018-06696-y
26 Gregis, V. *et al.* Identification of pathways directly regulated by SHORT VEGETATIVE PHASE during vegetative and reproductive development in Arabidopsis. *Genome Biology* **14**, R56 (2013). https://doi.org/10.1186/gb-2013-14-6-r56
27 Hartmann, U. *et al.* Molecular cloning of SVP: a negative regulator of the floral transition in Arabidopsis. *Plant Journal* **21**, 351-360 (2000). https://doi.org/10.1046/j.1365-313x.2000.00682.x
28 Castillejo, C. *et al.* The balance between CONSTANS and TEMPRANILLO activities determines FT expression to trigger flowering. *Current Biology* **18**, 1338-1343 (2008). https://doi.org/10.1016/j.cub.2008.07.075
29 Millar, A. J. *et al.* Circadian clock mutants in Arabidopsis identified by luciferase imaging. *Science* **267**, 1161-1163 (1995). https://doi.org/10.1126/science.7855595
30 Yanovsky, M. J. *et al.* Molecular basis of seasonal time measurement in Arabidopsis. *Nature* **419**, 308-312 (2002). https://doi.org/10.1038/nature00996
31 Stener, L.-G. *et al.* Resultat från klontester av hybridasp och poppel efter två års tillväxt. 30 (Skogforsk, 2018).
