# Supplementary material for: Nature’s Master of Ceremony: The Populus Circadian Clock as Orchestrator of Tree Growth and Phenology
Source: NPJ Biol Timing Sleep. 2025 Apr 7;2:16. doi: 10.1038/s44323-025-00034-4 (PMC11976295; doi:10.1038/s44323-025-00034-4)
Supplement: Supplementary file 3 — Supplementary information [file 44323_2025_34_MOESM3_ESM.zip › Natures_MC_data_and_scripts/Growth/FE1_height.html]

Field\_experiment\_(2014)


# Field\_experiment\_(2014)

### Part 1: given the treatment, do trees of a specific genotype show a unique growth pattern in their height?

#### Bertold Mariën

#### June 2023, University of Umea

# 1 **Field\_experiment\_(2014)**

## 1.1 **Given the treatment, do trees of a specific genotype show a unique growth pattern in their height?**

### 1.1.1 **Loading the data**

We begin with importing data.

```
data <- data.frame(read.csv("~/Umea_postdoc/Umea_data/CSV_datasets/Field_trials_growth_Ingrid.csv"))
head(data)
```

```
##   Column Row Year    Month Month_n Month_n_c Plot            Genotype
## 1      1  36 2015     June       6         8   11           Aet (8.2)
## 2      7  62 2015    April       4         6   18           KR941 (1)
## 3      8  60 2015    April       4         6   18 201 (35S:FT) (skum)
## 4     10  41 2014 November      11         1   12           Aet (8.2)
## 5     10  41 2015    April       4         6   12           Aet (8.2)
## 6      5   1 2014 November      11         1    1           Aet (8.2)
##   Individual Height..cm. Diameter..cm.
## 1        136          NA            NA
## 2        762          NA            NA
## 3        860           7            NA
## 4       1041          13            NA
## 5       1041          13            NA
## 6         51          14             2
```

```
names(data)
```

```
##  [1] "Column"        "Row"           "Year"          "Month"        
##  [5] "Month_n"       "Month_n_c"     "Plot"          "Genotype"     
##  [9] "Individual"    "Height..cm."   "Diameter..cm."
```

Our data contains eleven columns describing plant growth of tree
saplings in field conditions in 2014 and 2015. Each sampling date, the
height and/or diameter were measured in mm from each tree.

The first two columns return the position of the saplings, as does
the 7th plot column. The following four columns return the Year, Month,
Month number and the Month (starting from November 2014). The 8th column
describes the genotype (Genotype) of the trees that were measured; wild
type trees or mutant trees. The 9th (Individual) column shows the unique
tree number given to each tree individual of a particular genotype.
Finally, columns ten and eleven return the tree height (Height) and
diameter (Diameter) measured in cm.

The columns we will use are loaded and subsequently combined into a
data frame.

```
Month <- as.integer(as.character(data[,6])) 
Plot <- as.factor(as.character(data[,7]))
Genotype <- as.factor(as.character(data[,8]))
Individual <- as.factor(as.character(data[,9]))
Height <- as.numeric(as.character(data[,10]))
Diameter <- as.numeric(as.character(data[,11]))

data1 <- data.frame(Month, Plot, Genotype, Individual, Height, Diameter)
head(data1)
```

```
##   Month Plot            Genotype Individual Height Diameter
## 1     8   11           Aet (8.2)        136     NA       NA
## 2     6   18           KR941 (1)        762     NA       NA
## 3     6   18 201 (35S:FT) (skum)        860      7       NA
## 4     1   12           Aet (8.2)       1041     13       NA
## 5     6   12           Aet (8.2)       1041     13       NA
## 6     1    1           Aet (8.2)         51     14        2
```

```
names(data1)
```

```
## [1] "Month"      "Plot"       "Genotype"   "Individual" "Height"    
## [6] "Diameter"
```

### 1.1.2 **Loading the R packages**

Before starting any analyses, we also have to load multiple R
packages.

```
library(dplyr)
library(ggplot2)
library(mgcv)
library(dplyr)
library(gratia)
library(nlme)
library(MASS)
library(Rmisc)
library(mgcViz)
library(urca) 
library(fNonlinear) 
library(forecast) 
library(ggpubr)  
library(nortest) 
library(CADFtest) 
library(longmemo) 
library(goftest) 
library(mgcViz) 
library(hwwntest) 
library(normwhn.test) 
library(viridis)
library(e1071)
library(psych)
library(sur)
library(diptest)
library(modes)
library(multimode)
library(see)
library(ggeffects)
library(marginaleffects)
library(performance)
library(directlabels)
library(see)
library(ggeffects)
```

### 1.1.3 **Subset the data of interest & removing NA’s**

Now that the data and R packages are loaded, we can subset the part
of our data that interests us. In this particular case, we begin our
analyses by investigating whether trees of a specific genotype show a
unique growth patterns in their height.

```
data1a <- data.frame(Month, Plot, Genotype, Individual, Height)
head(data1a)
```

```
##   Month Plot            Genotype Individual Height
## 1     8   11           Aet (8.2)        136     NA
## 2     6   18           KR941 (1)        762     NA
## 3     6   18 201 (35S:FT) (skum)        860      7
## 4     1   12           Aet (8.2)       1041     13
## 5     6   12           Aet (8.2)       1041     13
## 6     1    1           Aet (8.2)         51     14
```

```
names(data1a)
```

```
## [1] "Month"      "Plot"       "Genotype"   "Individual" "Height"
```

```
data1aa <- subset(data1a, !(Genotype %in% c("201 (35S:FT) (\"normal\")",
                                            "201 (35S:FT) (skum)",
                                            "Aet (8.2)",
                                            "ami TEM (12)",
                                            "CO (21c)",
                                            "FIE::MSI2 (1)s (8A)",
                                            "KR940 (13)",
                                            "KR940 (2)",
                                            "KR940 (6)",
                                            "KR941 (1)",
                                            "KR941 (4)",
                                            "KR942 (14)",
                                            "mR156cX_35s (3)",
                                            "MSI1 (1A)",
                                            "MSI2 (1)",
                                            "PHYA (1 - 1A)",
                                            "TEM1AK (8)",
                                            "TEM1BK (5)")))
```

When the data is subset, we should remove those data that is
unavailable

```
data2 <- na.omit(data1aa)
head(data2)
```

```
##     Month Plot          Genotype Individual Height
## 22      6   17      ZTL (11 - 5)        461     17
## 96      1   11               T89        641     30
## 97      6   11               T89        641     30
## 110     1   16 HpFKF1 (124 - 11)        756     32
## 111     6   16 HpFKF1 (124 - 11)        756     32
## 116     8   16 HpFKF1 (124 - 11)        756     35
```

```
names(data2)
```

```
## [1] "Month"      "Plot"       "Genotype"   "Individual" "Height"
```

```
# Load dplyr library
library(dplyr)
```

### 1.1.4 **Plotting the data first**

#### 1.1.4.1 **Lineplot of the data per individual tree**

We can also create a lineplot for each individual tree.

```
plot <- ggplot(data = data2, aes(x= Month, y = Height, color = Individual)) +
  geom_line(size = 1.2) +
  ylab(label = 'Height (cm)') +
  geom_dl(aes(label=Individual, color = Individual, font = "bold"), method= list('top.bumptwice', cex = 0.5, hjust = -0.3, vjust = 0, rot = 30)) + 
  scale_x_continuous(name = "Month",
                     breaks =  seq(1,12,1), 
                     limits = c(0,13)) +
   scale_y_continuous(name = "Height (cm)",
                     breaks =  seq(0,300,50), 
                     limits = c(0,300)) +
  facet_wrap(. ~ Genotype)

plot <- plot + 
  theme_bw() +
  theme(panel.grid.major = element_blank(),
        panel.grid.minor = element_blank(),
        axis.title.x = element_text(size=18, face="bold"),
        axis.text.x = element_text(size=12, face="bold"),
        axis.title.y = element_text( size=18, face="bold"),
        axis.text.y = element_text(size=12, face="bold"),
        legend.title = element_text(size=14, face="bold"),
        legend.text = element_text(size=12, face="bold.italic"),
        strip.text = element_text(size=12, face = "bold.italic"),
        legend.position ="none") 
plot
```

#### 1.1.4.2 **Lineplot of the data per Genotype**

We can also create a lineplot for each genotype with standard
errors.

```
data2b=data2 %>%
  dplyr::group_by(Month, Genotype) %>%
  dplyr::summarise(mean_height = mean(Height), SDE_height = sd(Height)/sqrt(length(Height)))
data2c <- na.omit(data2b)

vir_col <- viridis(33, option = 'D')
cols <- c(
          "EBI1 (312 - 2)" = vir_col[5],
          "EBI1 (312 - 3)" = vir_col[6],
          "EBI2 (313 - 6)" = vir_col[7] ,
          "EBI2 (313 - 7)" = vir_col[8],
          "EBI2 (313 - 8)" = vir_col[9],
          "EBI2 (345 - 14)" = vir_col[10],
          "FT (16c)" = vir_col[12] ,
          "GI (12 - 13)" = vir_col[13],
          "HpCCA T89 (10)" = vir_col[14],
          "HpFKF1 (124 - 11)" = vir_col[15],
          "HpTOC1 (1 - 5)" = vir_col[16] ,
          "OxTOC1 (1119 - 7)" = vir_col[25] ,
          "PHYA (1 - 1A)" = vir_col[26],
          "PHYA (22)" = vir_col[27],
          "T89" = "black",
          "PRR7 (261 - 5)" = vir_col[28],
          "ZTL (11 - 3)" = vir_col[31],
          "ZTL (11 - 5)" = vir_col[32],
          "ZTL (11 - 7)" = vir_col[33])
labels <- c("EBI1 (312 - 2)" = expression(italic("ebi1-2")),
          "EBI1 (312 - 3)" = expression(italic("ebi1-3")),
          "EBI2 (313 - 6)" = expression(italic("ebi2-6")),
          "EBI2 (313 - 7)" = expression(italic("ebi2-7")),
          "EBI2 (313 - 8)" = expression(italic("ebi2-8")),
          "EBI2 (345 - 14)" = expression(italic("ebi1-14")),
          "FT (16c)" = expression(italic("ft-16")),
          "GI (12 - 13)" = expression(italic("gi-13")),
          "HpCCA T89 (10)" = expression(italic("lhy-10")),
          "HpFKF1 (124 - 11)" = expression(italic("fkf1-11")),
          "HpTOC1 (1 - 5)" = expression(italic("toc1-5")),
          "OxTOC1 (1119 - 7)" = expression(italic("TOC1_OX-7")),
          "PHYA (1 - 1A)" = expression(italic("pAtCCR2::LUC PHYA1-1")),
          "PHYA (22)" = expression(italic("pAtCCR2::LUC PHYA22-2")),
          "T89" = expression(italic("WT  (T89)")),
          "PRR7 (261 - 5)" = expression(italic("prr7-5")),
          "ZTL (11 - 3)" = expression(italic("ztl-3")),
          "ZTL (11 - 5)" = expression(italic("ztl-5")),
          "ZTL (11 - 7)" = expression(italic("ztl-7")))

plot <- ggplot(data = data2c, aes(x= Month, y = mean_height,
                                      color = Genotype,
                                      fill= Genotype)) +
  geom_point(aes(x=Month,
                 y=mean_height, shape = Genotype), size=3) +
  geom_line(aes(x=Month,
                y=mean_height), size=1) +
  geom_errorbar(aes(x=Month, 
                    ymin=mean_height-SDE_height,
                    ymax=mean_height+SDE_height),
                size=1, width=0.6) +
  ylab(label = 'Height (cm)') +
  scale_x_continuous(name = "Month",
                     breaks =  seq(1,12,1), 
                     limits = c(0.5,12.5)) +
   scale_y_continuous(name = "Height (cm)",
                     breaks =  seq(50,200,50), 
                     limits = c(50,220))

plot <- plot +
  scale_color_manual(name = "Genotype",
                     values = cols,
                     labels = labels) +
    scale_fill_manual(name = "Genotype",
                    values = cols,
                    labels = labels) 

plot <- plot + 
  theme_bw() +
  theme(panel.grid.major = element_blank(),
        panel.grid.minor = element_blank(),
        axis.title.x = element_text(size=18, face="bold"),
        axis.text.x = element_text(size=12, face="bold"),
        axis.title.y = element_text( size=18, face="bold"),
        axis.text.y = element_text(size=12, face="bold"),
        legend.title = element_text(size=14, face="bold"),
        legend.text = element_text(size=12, face="bold.italic"),
        strip.text = element_text(size=12, face = "bold.italic"),
        legend.position ="right") 
plot
```

```
View(data2)
```

```
# Save
png('C:/Users/berthold/Documents/Umea_postdoc/Umea_markdown_documents/Ingrid_data_height_F2.png',
    units = 'px', width=7000, height=5000, res=600)
plot(plot)
dev.off()
```

```
## png 
##   2
```

### 1.1.5 **Making the GAM(M) model**

First, we decide whether we want to use R/mgcv’ *gam* or
*gamm* function. Remember that generalized additive mixed models
(GAMMs), unlike GAMs, allow to address the residual temporal
autocorrelation by implementing autoregressive or moving average terms
(Wood, 2004). There are also some small differences in the arguments
used for each function.

To model the *height* as a function of its covariates, we
decided to use the *gam* function in the package R/mgcv to build
GAMMs (Wood, 2017; Wood, 2004; Wood, 2011; Zuur et al., 2007). The fixed
covariates of the Height were the *Genotype* (categorical with 33
levels) and *Month* (continuous). The interaction term was
modeled as a factor-smooth interaction between the covariates
*Genotype* and *Month*, and smoothed using Random factor
smooth interaction smoothers. Finally, the dependency among observations
of the same site was incorporated by using *Individual* as random
effect (a coefficient for each level). The interaction between the
*Plot* and *Individual* could note be included as random
effect due to issue that the model would have more coefficients than
data.

```
m10 <- gam(list(Height ~  s(Month, Genotype, bs = 'fs', k =4)  +
            s(Individual, bs = "re"),
          ~ s(Month, Genotype, bs = 'fs', k = 4)  +
            s(Individual, bs = "re")),
            family = gaulss(link=list("sqrt", "logb")), 
            method = "REML",
          data = data2)
```

#### 1.1.5.1 **Assessing smoother dynamics of GAMMs**

We assessed the factor-smooth interaction smoother dynamics of the
GAMM model using the *smooth\_estimate* function from the R/gratia
package (Simpson, 2020). This function computes the Bayesian credible
intervals for the smooth terms of a fitted GAM model.

```
# Get the smooth estimates and CIs
sm1 <- smooth_estimates(m10, "Month", partial_match = TRUE) |>
    add_confint() |>
    add_constant(coef(m10)[1]) |>
    transform_fun(inv_link(m10)) 

# Subset the relevant smoothers
sm2 <- subset(sm1, .type == "Factor smooth")
sm3 <- subset(sm2, .smooth != "s.1(Month,Genotype)")

# Make the plot
vir_col <- viridis(33, option = 'D')
cols <- c(
          "EBI1 (312 - 2)" = vir_col[5],
          "EBI1 (312 - 3)" = vir_col[6],
          "EBI2 (313 - 6)" = vir_col[7] ,
          "EBI2 (313 - 7)" = vir_col[8],
          "EBI2 (313 - 8)" = vir_col[9],
          "EBI2 (345 - 14)" = vir_col[10],
          "FT (16c)" = vir_col[12] ,
          "GI (12 - 13)" = vir_col[13],
          "HpCCA T89 (10)" = vir_col[14],
          "HpFKF1 (124 - 11)" = vir_col[15],
          "HpTOC1 (1 - 5)" = vir_col[16] ,
          "OxTOC1 (1119 - 7)" = vir_col[25] ,
          "PHYA (1 - 1A)" = vir_col[26],
          "PHYA (22)" = vir_col[27],
          "T89" = "black",
          "PRR7 (261 - 5)" = vir_col[28],
          "ZTL (11 - 3)" = vir_col[31],
          "ZTL (11 - 5)" = vir_col[32],
          "ZTL (11 - 7)" = vir_col[33])
labels <- c("EBI1 (312 - 2)" = expression(italic("ebi1-2")),
          "EBI1 (312 - 3)" = expression(italic("ebi1-3")),
          "EBI2 (313 - 6)" = expression(italic("ebi2-6")),
          "EBI2 (313 - 7)" = expression(italic("ebi2-7")),
          "EBI2 (313 - 8)" = expression(italic("ebi2-8")),
          "EBI2 (345 - 14)" = expression(italic("ebi1-14")),
          "FT (16c)" = expression(italic("ft-16")),
          "GI (12 - 13)" = expression(italic("gi-13")),
          "HpCCA T89 (10)" = expression(italic("lhy-10")),
          "HpFKF1 (124 - 11)" = expression(italic("fkf1-11")),
          "HpTOC1 (1 - 5)" = expression(italic("toc1-5")),
          "OxTOC1 (1119 - 7)" = expression(italic("TOC1_OX-7")),
          "PHYA (1 - 1A)" = expression(italic("pAtCCR2:LUC PHYA1-1")),
          "PHYA (22)" = expression(italic("pAtCCR2:LUC PHYA22-2")),
          "T89" = expression(italic("WT (T89)")),
          "PRR7 (261 - 5)" = expression(italic("prr7-5")),
          "ZTL (11 - 3)" = expression(italic("ztl-3")),
          "ZTL (11 - 5)" = expression(italic("ztl-5")),
          "ZTL (11 - 7)" = expression(italic("ztl-7")))

plt <- ggplot(data = sm3, aes(x= Month, y = .estimate,
                                      color = Genotype,
                                      fill=  Genotype)) +
    geom_line(size = 1.2) +
    geom_ribbon(data = sm3,
                  aes(ymin = .lower_ci, ymax = .upper_ci),
                  alpha = 0.3) +
  ylab(label = 'Height (cm)') +
  scale_x_continuous(name = "Month",
                     breaks =  seq(1,12,1), 
                     limits = c(1,12)) +
   scale_y_continuous(name = "Height (cm)",
                     breaks =  seq(0,250,50), 
                     limits = c(0,250)) 

plt <- plt +
  scale_color_manual(name = "Genotype",
                     values = cols,
                     labels = labels) +
  scale_fill_manual(name = "Genotype",
                    values = cols,
                    labels = labels) 

plt <- plt + 
  theme_bw() +
  theme(panel.grid.major = element_blank(),
        panel.grid.minor = element_blank(),
        axis.title.x = element_text(size=18, face="bold"),
        axis.text.x = element_text(size=12, face="bold"),
        axis.title.y = element_text( size=18, face="bold"),
        axis.text.y = element_text(size=12, face="bold"),
        legend.title = element_text(size=14, face="bold"),
        legend.text = element_text(size=12, face="bold.italic"),
        strip.text = element_text(size=12, face = "bold.italic"),
        legend.position ="right") 
plt
```

```
# Save
png('C:/Users/bema2731/Documents/Umea_postdoc/Umea_markdown_documents/Ingrid_term_plot_height_F.png',
    units = 'px', width=7000, height=4000, res=600)
plot(plt)
dev.off()
```

```
## png 
##   2
```

### 1.1.6 **Searching for significant differences in the smoothers I**

To observe significant differences among the smoothers, we will
follow the procedure outlined in (Rose et al., 2012) and Gavin Simpson’s
blog ‘Comparing smooths in factor-smooth interactions II’. Since we have
a clear reference genotype (WT) this should make sense.

### 1.1.7 **Ordering the factors in the factor-smooth interaction smoother**

Trees with the WT genotype (T89) are set as the reference.

```
data3 <- mutate(data2,
                 oGenotype = ordered(Genotype, levels = c("T89", "EBI1 (312 - 2)", "EBI1 (312 - 3)", "EBI2 (313 - 6)", "EBI2 (313 - 7)", "EBI2 (313 - 8)", "EBI2 (345 - 14)", "FT (16c)", "GI (12 - 13)", "HpCCA T89 (10)", "HpFKF1 (124 - 11)", "HpTOC1 (1 - 5)", "OxTOC1 (1119 - 7)", "PHYA (1 - 1A)", "PHYA (22)", "PRR7 (261 - 5)", "ZTL (11 - 3)", "ZTL (11 - 5)", "ZTL (11 - 7)")))
head(data3)
```

```
##     Month Plot          Genotype Individual Height         oGenotype
## 22      6   17      ZTL (11 - 5)        461     17      ZTL (11 - 5)
## 96      1   11               T89        641     30               T89
## 97      6   11               T89        641     30               T89
## 110     1   16 HpFKF1 (124 - 11)        756     32 HpFKF1 (124 - 11)
## 111     6   16 HpFKF1 (124 - 11)        756     32 HpFKF1 (124 - 11)
## 116     8   16 HpFKF1 (124 - 11)        756     35 HpFKF1 (124 - 11)
```

#### 1.1.7.1 **Running the GAMM again**

A quick term plot (on the link scale) can be made using the
*plot* function from R/mgcv. Given the argument seWithMean, the
confidence intervals represent Bayesian Wahba/Silverman confidence
intervals on smooths (Wahba, 1983; Silverman, 1985; Nychka, 1988).

```
# Run the model
m10b <- gam(list(Height ~ s(Month, k = 4) +
            s(Month, oGenotype, bs = 'fs', k = 3)  +
            s(Individual, bs = "re"),
          ~ s(Month, k = 4) + 
            s(Month, oGenotype, bs = 'fs', k = 3)  +
            s(Individual, bs = "re")),
            family = gaulss(link=list("sqrt", "logb")), 
            method = "REML",
          data = data3)

# Get approximate p-values
summary(m10b)
```

```
## 
## Family: gaulss 
## Link function: sqrt logb 
## 
## Formula:
## Height ~ s(Month, k = 4) + s(Month, oGenotype, bs = "fs", k = 3) + 
##     s(Individual, bs = "re")
## ~s(Month, k = 4) + s(Month, oGenotype, bs = "fs", k = 3) + s(Individual, 
##     bs = "re")
## 
## Parametric coefficients:
##               Estimate Std. Error z value Pr(>|z|)    
## (Intercept)    10.8550     0.2132  50.909  < 2e-16 ***
## (Intercept).1  -7.5409     0.9716  -7.761  8.4e-15 ***
## ---
## Signif. codes:  0 '***' 0.001 '**' 0.01 '*' 0.05 '.' 0.1 ' ' 1
## 
## Approximate significance of smooth terms:
##                          edf  Ref.df    Chi.sq  p-value    
## s(Month)               2.996   2.997 8.908e+02  < 2e-16 ***
## s(Month,oGenotype)    48.157  33.000 2.526e+15  < 2e-16 ***
## s(Individual)        388.072 400.000 1.893e+09  < 2e-16 ***
## s.1(Month)             2.972   2.980 2.895e+02  < 2e-16 ***
## s.1(Month,oGenotype)  48.471  52.000 4.483e+04 2.02e-05 ***
## s.1(Individual)      175.148 399.000 3.735e+03  0.00309 ** 
## ---
## Signif. codes:  0 '***' 0.001 '**' 0.01 '*' 0.05 '.' 0.1 ' ' 1
## 
## Deviance explained =  100%
## -REML = 6370.3  Scale est. = 1         n = 2022
```

```
# Get the smooth estimates and CIs
sm1 <- smooth_estimates(m10b, "Month", partial_match = TRUE) |>
    add_confint()  

# Subset the relevant smoothers
sm2 <- subset(sm1, .type == "Factor smooth")
sm3 <- subset(sm2, .smooth != "s.1(Month,oGenotype)")
sm4 <- subset(sm3, oGenotype != "T89")

# Make the plot
#Increase factor levels
levels(sm4$oGenotype) <- c(levels(sm4$oGenotype), "ebi1-2", "ebi1-3","ebi2-6", "ebi2-7", "ebi2-8", "ebi1-14", "ft-16", "gi-13", "lhy-10","fkf1-11","toc1-5", "TOC1_OX-7","pAtCCR2:LUC PHYA1-1","pAtCCR2:LUC PHYA22-2","prr7-5", "ztl-3", "ztl-5", "ztl-7")

# Change levels
sm4$oGenotype[sm4$oGenotype == "EBI1 (312 - 2)"] <- "ebi1-2"
sm4$oGenotype[sm4$oGenotype == "EBI1 (312 - 3)"] <- "ebi1-3"
sm4$oGenotype[sm4$oGenotype == "EBI2 (313 - 6)"] <- "ebi2-6"
sm4$oGenotype[sm4$oGenotype == "EBI2 (313 - 7)"] <- "ebi2-7"
sm4$oGenotype[sm4$oGenotype == "EBI2 (313 - 8)"] <- "ebi2-8"
sm4$oGenotype[sm4$oGenotype == "EBI2 (345 - 14)"] <- "ebi1-14"
sm4$oGenotype[sm4$oGenotype == "FT (16c)"] <- "ft-16"
sm4$oGenotype[sm4$oGenotype == "GI (12 - 13)"] <- "gi-13"
sm4$oGenotype[sm4$oGenotype == "HpCCA T89 (10)"] <- "lhy-10"
sm4$oGenotype[sm4$oGenotype == "HpFKF1 (124 - 11)"] <- "fkf1-11"
sm4$oGenotype[sm4$oGenotype == "HpTOC1 (1 - 5)"] <- "toc1-5"
sm4$oGenotype[sm4$oGenotype == "OxTOC1 (1119 - 7)"] <- "TOC1_OX-7"
sm4$oGenotype[sm4$oGenotype == "PHYA (1 - 1A)"] <- "pAtCCR2:LUC PHYA1-1"
sm4$oGenotype[sm4$oGenotype == "PRR7 (261 - 5)"] <- "prr7-5"
sm4$oGenotype[sm4$oGenotype == "PHYA (22)"] <- "pAtCCR2:LUC PHYA22-2"
sm4$oGenotype[sm4$oGenotype == "ZTL (11 - 3)"] <- "ztl-3"
sm4$oGenotype[sm4$oGenotype == "ZTL (11 - 5)"] <- "ztl-5"
sm4$oGenotype[sm4$oGenotype == "ZTL (11 - 7)"] <- "ztl-7"

#  Change colors
vir_col <- viridis(33, option = 'D')
cols <- c(
          "EBI1 (312 - 2)" = vir_col[5],
          "EBI1 (312 - 3)" = vir_col[6],
          "EBI2 (313 - 6)" = vir_col[7] ,
          "EBI2 (313 - 7)" = vir_col[8],
          "EBI2 (313 - 8)" = vir_col[9],
          "EBI2 (345 - 14)" = vir_col[10],
          "FT (16c)" = vir_col[12] ,
          "GI (12 - 13)" = vir_col[13],
          "HpCCA T89 (10)" = vir_col[14],
          "HpFKF1 (124 - 11)" = vir_col[15],
          "HpTOC1 (1 - 5)" = vir_col[16] ,
          "OxTOC1 (1119 - 7)" = vir_col[25] ,
          "PHYA (1 - 1A)" = vir_col[26],
          "PHYA (22)" = vir_col[27],
          #"T89" = "black",
          "PRR7 (261 - 5)" = vir_col[28],
          "ZTL (11 - 3)" = vir_col[31],
          "ZTL (11 - 5)" = vir_col[32],
          "ZTL (11 - 7)" = vir_col[33])

plt <- ggplot(data = sm4, aes(x= Month, y = .estimate,
                                      color = oGenotype,
                                      fill=  oGenotype)) +
    geom_line(size = 1.2) +
    geom_ribbon(data = sm4,
                  aes(ymin = .lower_ci, ymax = .upper_ci),
                  alpha = 0.3) +
  ylab(label = 'difference in the height trend (link scale)') +
  geom_hline(yintercept=0,linetype="dashed", color = "red", size = 1) +
  scale_x_continuous(name = "Month",
                     breaks =  seq(1,12,1), 
                     limits = c(1, 12)) +
   scale_y_continuous(name = "Difference in the height trend (link scale)",
                     breaks =  seq(-6,4,2), 
                     limits = c(-6,4)) +
  facet_wrap(.~ oGenotype)

plt <- plt + 
  theme_bw() +
  theme(panel.grid.major = element_blank(),
        panel.grid.minor = element_blank(),
        axis.title.x = element_text(size=18, face="bold"),
        axis.text.x = element_text(size=12, face="bold"),
        axis.title.y = element_text( size=18, face="bold"),
        axis.text.y = element_text(size=12, face="bold"),
        legend.title = element_text(size=10, face="bold"),
        legend.text = element_text(size=6, face="bold.italic"),
        strip.text = element_text(size=12, face = "bold.italic"),
        legend.position ="bottom") 
plt
```

```
# Save
png('C:/Users/bema2731/Documents/Umea_postdoc/Umea_markdown_documents/Ingrid_differences_height_F_new.png',
    units = 'px', width=13000, height=6000, res=600)
plot(plt)
dev.off()
```

```
## png 
##   2
```

### 1.1.8 **Assessing the assumptions of a GAM(M) model**

The validity of any model depends on the extent to which the
residuals meet the underlying statistical assumptions **(i.e. in
order of importance: outliers, homogeneity, normality, zero adjusting,
collinearity, interactions, independence)** because departures
from these assumptions might increase Type I or II errors, or affect the
estimation of the effect size or its significance (Osbourne and Waters,
2002; Zuur et al., 2016). Therefore, although knowing the extent to
which departures from the assumptions affect the results is not
straightforward, it is important that the underlying assumptions of the
residuals are thoroughly tested - and reported - based on an ‘a priori’
decision for the tested model (Barker and Shaw, 2015; Burnham and
Anderson, 2004; Harrison et al., 2018; Schielzeth et al., 2020).

In this study, we extracted the model residuals using the residuals
functions from the R/mgcv package (Wood, 2017).The parametric
assumptions of the model can be ensured using R/mgcv’s *summary*
function.

#### 1.1.8.1 **General diagnostic plots and results**

The general results are extracted using the *summary*
function.

```
summary(m10b)
```

```
## 
## Family: gaulss 
## Link function: sqrt logb 
## 
## Formula:
## Height ~ s(Month, k = 4) + s(Month, oGenotype, bs = "fs", k = 3) + 
##     s(Individual, bs = "re")
## ~s(Month, k = 4) + s(Month, oGenotype, bs = "fs", k = 3) + s(Individual, 
##     bs = "re")
## 
## Parametric coefficients:
##               Estimate Std. Error z value Pr(>|z|)    
## (Intercept)    10.8550     0.2132  50.909  < 2e-16 ***
## (Intercept).1  -7.5409     0.9716  -7.761  8.4e-15 ***
## ---
## Signif. codes:  0 '***' 0.001 '**' 0.01 '*' 0.05 '.' 0.1 ' ' 1
## 
## Approximate significance of smooth terms:
##                          edf  Ref.df    Chi.sq  p-value    
## s(Month)               2.996   2.997 8.908e+02  < 2e-16 ***
## s(Month,oGenotype)    48.157  33.000 2.526e+15  < 2e-16 ***
## s(Individual)        388.072 400.000 1.893e+09  < 2e-16 ***
## s.1(Month)             2.972   2.980 2.895e+02  < 2e-16 ***
## s.1(Month,oGenotype)  48.471  52.000 4.483e+04 2.02e-05 ***
## s.1(Individual)      175.148 399.000 3.735e+03  0.00309 ** 
## ---
## Signif. codes:  0 '***' 0.001 '**' 0.01 '*' 0.05 '.' 0.1 ' ' 1
## 
## Deviance explained =  100%
## -REML = 6370.3  Scale est. = 1         n = 2022
```

Note here, that the effective degrees of freedom (edf) returned here
gives an approximate idea of the complexity of the smoother (i.e. the
number of required basis functions).

A quick term plot can be made using the *plot* function from
R/mgcv. Given the argument seWithMean, the confidence intervals
represent standard Wahba/Silverman type Bayesian confidence intervals on
smooths with a reasonable frequentist across-the-function
interpretation, if the smooths are not overly smoothed (Nychka,
1988).

```
plot(m10b, rug = TRUE, all.terms = TRUE, pages = 1, seWithMean = TRUE, shade = TRUE, shade.col = "hotpink")
```

We can also test for concurvity and collinearity using the
*concurvity* function in R/mgcv.

```
concurvity(m10b, full = TRUE)
```

```
##          para s(Month) s(Month,oGenotype) s(Individual) s.1(Month)
## worst       1        1                  1             1          1
## observed    1        1                  1             1          1
## estimate    1        1                  1             1          1
##          s.1(Month,oGenotype) s.1(Individual)
## worst                       1               1
## observed                    1               1
## estimate                    1               1
```

Values > 0,8 would be an indicator of concurvity/collinearity. Due
to the use of a location-scale distribution, this function does not
behave as expected.

#### 1.1.8.2 **non-linearity of the residuals in GAM(M)s**

We expected the residuals of our data to be non-linear. We tested the
residuals of our models for non-linearity using the *bdsTest*
function (Brock-Dechert-Scheinkmand and LeBaron statistic test) in the
R/fNonlinear package (Wuertz et al., 2017).

```
BDS_test <- bdsTest(residuals(m10b))
BDS_test
```

```
## 
## Title:
##  BDS Test
## 
## Test Results:
##   PARAMETER:
##     Max Embedding Dimension: 3
##     eps[1]: 0.404
##     eps[2]: 0.808
##     eps[3]: 1.211
##     eps[4]: 1.615
##   STATISTIC:
##     eps[1] m=2: 18.4949
##     eps[1] m=3: 18.6755
##     eps[2] m=2: 7.2015
##     eps[2] m=3: 7.3752
##     eps[3] m=2: 1.5719
##     eps[3] m=3: 0.5322
##     eps[4] m=2: 0.9182
##     eps[4] m=3: 0.1306
##   P VALUE:
##     eps[1] m=2: < 2.2e-16 
##     eps[1] m=3: < 2.2e-16 
##     eps[2] m=2: 5.956e-13 
##     eps[2] m=3: 1.641e-13 
##     eps[3] m=2: 0.116 
##     eps[3] m=3: 0.5946 
##     eps[4] m=2: 0.3585 
##     eps[4] m=3: 0.8961 
## 
## Description:
##  Fri Jan 24 00:24:07 2025 by user: Berthold
```

As the p-values were lower than 0.05, we concluded that the residuals
of our models were indeed non-linear.

#### 1.1.8.3 **Normality of the residuals in GAM(M)s**

An important model assumption, in particular because the central
limit theory is not valid at small sample sizes, is that the residuals
of our models were normally distributed. To test this assumption, we
first visually inspected the histograms and qq-plots given by the
*gam.check* function in the package R/mgcv (Wood, 2017). The
*gam.check* function also allowed to test whether the basis
dimension for the smoother sufficed by returning the k-index and
according p-value (Pya and Wood, 2016).

```
par(mfrow = c(2,2))
gam.check(m10b)
```

```
## 
## Method: REML   Optimizer: outer newton
## full convergence after 22 iterations.
## Gradient range [-1.565533e-05,0.000248916]
## (score 6370.321 & scale 1).
## Hessian positive definite, eigenvalue range [0.9516844,192.9613].
## Model rank =  916 / 916 
## 
## Basis dimension (k) checking results. Low p-value (k-index<1) may
## indicate that k is too low, especially if edf is close to k'.
## 
##                          k'    edf k-index p-value    
## s(Month)               3.00   3.00    0.62  <2e-16 ***
## s(Month,oGenotype)    54.00  48.16    0.62  <2e-16 ***
## s(Individual)        400.00 388.07      NA      NA    
## s.1(Month)             3.00   2.97    0.62  <2e-16 ***
## s.1(Month,oGenotype)  54.00  48.47    0.62  <2e-16 ***
## s.1(Individual)      400.00 175.15      NA      NA    
## ---
## Signif. codes:  0 '***' 0.001 '**' 0.01 '*' 0.05 '.' 0.1 ' ' 1
```

A low p-value in combination with a k-index lower than 1 would
suggest that the basis dimensions of our smoothers were too low.

More robust methods to assess the assumption of normality in our
model assumptions, even though hampered by the small sample size, was to
test the residuals using the *shapiro.test* (Shapiro-Wilk test),
*ad.test* (Anderson-Darling test) and *cvm.test*
(Cramer-Von-Mises test) functions in the R/base, R/nortest and R/goftest
packages, respectively (Faraway et al., 2019; Gross and Ligges, 2015;
Shapiro and Wilk, 1965). The Anderson-Darling test has as H0 that our
data has a uniform distribution, while the shapiro-wilk and
CrameR-Von-Mises test have as H0 that the data follows a normal
distribution!

```
shapiro.test(residuals(m10b))
```

```
ad.test(residuals(m10b))
```

```
## 
##  Anderson-Darling test of goodness-of-fit
##  Null hypothesis: uniform distribution
##  Parameters assumed to be fixed
## 
## data:  residuals(m10b)
## An = Inf, p-value = 2.967e-07
```

```
cvm.test(residuals(m10b), null = 'pnorm')
```

```
## 
##  Cramer-von Mises test of goodness-of-fit
##  Null hypothesis: Normal distribution
##  Parameters assumed to be fixed
## 
## data:  residuals(m10b)
## omega2 = 22.025, p-value < 2.2e-16
```

#### 1.1.8.4 **Homogeneity of variance in GAM(M)s**

Another assumption that had to be tested to assess the fit of our
models is to check whether the residuals of our models show
homoscedasticity. To do this, the residuals vs. linear predictor plot
and response vs. fitted values plot, which are part of the
*gam.check* function output (see above), were used.

```
par(mfrow = c(2,2))
gam.check(m10b)
```

```
## 
## Method: REML   Optimizer: outer newton
## full convergence after 22 iterations.
## Gradient range [-1.565533e-05,0.000248916]
## (score 6370.321 & scale 1).
## Hessian positive definite, eigenvalue range [0.9516844,192.9613].
## Model rank =  916 / 916 
## 
## Basis dimension (k) checking results. Low p-value (k-index<1) may
## indicate that k is too low, especially if edf is close to k'.
## 
##                          k'    edf k-index p-value    
## s(Month)               3.00   3.00    0.62  <2e-16 ***
## s(Month,oGenotype)    54.00  48.16    0.62  <2e-16 ***
## s(Individual)        400.00 388.07      NA      NA    
## s.1(Month)             3.00   2.97    0.62  <2e-16 ***
## s.1(Month,oGenotype)  54.00  48.47    0.62  <2e-16 ***
## s.1(Individual)      400.00 175.15      NA      NA    
## ---
## Signif. codes:  0 '***' 0.001 '**' 0.01 '*' 0.05 '.' 0.1 ' ' 1
```

Are in the residuals vs. linear predictor plot the residuals
relatively equally spread around zero or do they show an irregular
pattern.

To further inspect the heteroscedasticity in the residuals, we used
the *getViz*, *check1D*, *l\_densCheck* and
*l\_gridCheck1D* functions in the R/mgcViz package (Fasiolo et
al., 2019). These plots also showed that the residuals were
heteroscedastic.

```
b <- getViz(m10b, nsim=200)
check1D(b, "Month") + 
  l_gridCheck1D(gridFun = sd, showReps = TRUE, stand = "sc")
```

```
b <- getViz(m10b)
check1D(b, "Month") + 
  l_densCheck()
```

The R/mgcViz package mentions that the latter plot “calculates and
plots how the empirical conditional density of the residuals, r, differs
from its theoretical or model-based counterpart, along a covariate, x.
(p.24 ) and that”(Red) Blue indicates area where the empirical density
of the residuals is (lower) higher than it should be under the model
(p. 25)“. In other words, there are few signs of heteroscedasticity
here.

#### 1.1.8.5 **Independent and identically distributed data**

The trend of growth in plants should show clear serial correlation,
as the growth values at any given moment are dependent on the previous
growth values.

To test whether the residuals of our models showed serial correlation
and were non-i.i.d., we used the Haar Wavelet White Noise test from the
R/hwwntest package (Nason and Savchev, 2014; Savchev and Nason, 2018).
In practice, we used the *hwwn.test* function to test the null
hypothesis that the underlying spectrum of the time series were flat
(i.e. our time series showed a mean of zero, a constant standard
deviation and no autocorrelation). Alternatively, to test for white
noise in the residuals of our models, we also used the
*whitenoise.test* function from the R/normwhn.test package and
the *Box.test* function (Ljung-Box test) (Box and Pierce, 1970;
Ljung and Box, 1978; Wickham, 2012).

```
zero_pad <- rep(0,99)
data.merge <- c(residuals(m10b),zero_pad)
whitenoise.test(residuals(m10b))
```

```
## [1] "no. of observations"
## [1] 2022
## [1] "T"
## [1] 1011
## [1] "CVM stat MN"
## [1] 1.621566
## [1] "tMN"
## [1] 19.76346
## [1] "test value"
## [1] 4.996302e-23
```

```
Box.test(residuals(m10b), type="Ljung-Box")
```

```
## 
##  Box-Ljung test
## 
## data:  residuals(m10b)
## X-squared = 72.996, df = 1, p-value < 2.2e-16
```

#### 1.1.8.6 **Autocorrelation**

I.i.d. in the model residuals is an important model assumption that
should not be ignored. Here, we address the presenceof residual temporal
autocorrelation in our GAMM models.

To begin, we visually inspected the residuals of our model using the
*acf* and *pacf* functions. Note that ideally we want to
look here at the standardized residual ACF and pACF plots, rather than
the raw residual ACF and pACF plots. The autocorrelation graphs helps in
assessing the moving average order (q) and the differencing parameter
(d), while the partial autocorrelation graph helps in assessing the
autoregressive order (p).

```
layout(matrix(1:2, ncol = 2)) 
acf(residuals(m10b), lag.max = 12, main = "raw residual ACF")
pacf(residuals(m10b), lag.max = 12, main= "raw residual pACF")
```

```
layout(1)
```

#### 1.1.8.7 **Testing for stationarity**

First, we performed the KPSS (Kwiatkowski-Phillips-Schmidt-Shin) test
for trend-stationarity using the *ur.kpss* function (Bhargava,
1986; Kwiatkowski et al., 1992). The null hypothesis of the KPSS test is
that there is no unit root (i.e. a stochastic trend) in the time
series.

```
KPSS_test <- ur.kpss(residuals(m10b), type = "tau", lags = "short")
KPSS_test
```

```
## 
## ####################################### 
## # KPSS Unit Root / Cointegration Test # 
## ####################################### 
## 
## The value of the test statistic is: 1.6958
```

```
summary(KPSS_test)
```

```
## 
## ####################### 
## # KPSS Unit Root Test # 
## ####################### 
## 
## Test is of type: tau with 8 lags. 
## 
## Value of test-statistic is: 1.6958 
## 
## Critical value for a significance level of: 
##                 10pct  5pct 2.5pct  1pct
## critical values 0.119 0.146  0.176 0.216
```

```
plot(KPSS_test)
```

If the absolute value of the test statistic is lower than the
absolute value of the 0.05 critical values, we can conclude that the
differentiated model shows trend stationarity.

Subsequently, we performed the PP (Phillips-Perron) test, ADF
(Augmented-Dickey-Fuller) test and the ERS (Elliot-Rothenberg and Stock
Point Optimal) or ADF-GLS test for a unit root in the time series using
the *ur.pp*, *ur.df* and *ur.ers* functions,
respectively (Bhargava, 1986; Elliott et al., 1996; Phillips and Perron,
1988). For these three tests, the null hypothesis is that a unit root is
present in the time series.

```
PP_test <- ur.pp(residuals(m10b), type = "Z-tau",
                 lags = "short")
PP_test
```

```
## 
## ################################################## 
## # Phillips-Perron Unit Root / Cointegration Test # 
## ################################################## 
## 
## The value of the test statistic is: -40.1155
```

```
summary(PP_test)
```

```
## 
## ################################## 
## # Phillips-Perron Unit Root Test # 
## ################################## 
## 
## Test regression with intercept 
## 
## 
## Call:
## lm(formula = y ~ y.l1)
## 
## Residuals:
##     Min      1Q  Median      3Q     Max 
## -4.6511 -0.2122 -0.0686  0.4627  4.1837 
## 
## Coefficients:
##             Estimate Std. Error t value Pr(>|t|)    
## (Intercept)  0.07028    0.01775   3.960 7.75e-05 ***
## y.l1         0.19009    0.02186   8.695  < 2e-16 ***
## ---
## Signif. codes:  0 '***' 0.001 '**' 0.01 '*' 0.05 '.' 0.1 ' ' 1
## 
## Residual standard error: 0.7933 on 2019 degrees of freedom
## Multiple R-squared:  0.03609,    Adjusted R-squared:  0.03561 
## F-statistic:  75.6 on 1 and 2019 DF,  p-value: < 2.2e-16
## 
## 
## Value of test-statistic, type: Z-tau  is: -40.1155 
## 
##          aux. Z statistics
## Z-tau-mu            4.2854
## 
## Critical values for Z statistics: 
##                      1pct      5pct     10pct
## critical values -3.436475 -2.863457 -2.567813
```

```
plot(PP_test)
```

```
ADF_test <- ur.df(residuals(m10b), type = "none", selectlags = "AIC") #@lags
ADF_test
```

```
## 
## ############################################################### 
## # Augmented Dickey-Fuller Test Unit Root / Cointegration Test # 
## ############################################################### 
## 
## The value of the test statistic is: -24.5504
```

```
summary(ADF_test)
```

```
## 
## ############################################### 
## # Augmented Dickey-Fuller Test Unit Root Test # 
## ############################################### 
## 
## Test regression none 
## 
## 
## Call:
## lm(formula = z.diff ~ z.lag.1 - 1 + z.diff.lag)
## 
## Residuals:
##     Min      1Q  Median      3Q     Max 
## -4.5808 -0.1788  0.0255  0.4926  4.2443 
## 
## Coefficients:
##            Estimate Std. Error t value Pr(>|t|)    
## z.lag.1    -0.68532    0.02791 -24.550  < 2e-16 ***
## z.diff.lag -0.14396    0.02205  -6.529 8.36e-11 ***
## ---
## Signif. codes:  0 '***' 0.001 '**' 0.01 '*' 0.05 '.' 0.1 ' ' 1
## 
## Residual standard error: 0.7883 on 2018 degrees of freedom
## Multiple R-squared:  0.4124, Adjusted R-squared:  0.4119 
## F-statistic: 708.3 on 2 and 2018 DF,  p-value: < 2.2e-16
## 
## 
## Value of test-statistic is: -24.5504 
## 
## Critical values for test statistics: 
##       1pct  5pct 10pct
## tau1 -2.58 -1.95 -1.62
```

```
plot(ADF_test)
```

```
ERS_test <- ur.ers(residuals(m10b), type = c("DF-GLS", "P-test")) 
ERS_test
```

```
## 
## ############################################################### 
## # Elliot, Rothenberg and Stock Unit Root / Cointegration Test # 
## ############################################################### 
## 
## The value of the test statistic is: -13.133
```

```
summary(ERS_test)
```

```
## 
## ############################################### 
## # Elliot, Rothenberg and Stock Unit Root Test # 
## ############################################### 
## 
## Test of type DF-GLS 
## detrending of series with intercept 
## 
## 
## Call:
## lm(formula = dfgls.form, data = data.dfgls)
## 
## Residuals:
##     Min      1Q  Median      3Q     Max 
## -4.5411 -0.1887  0.0862  0.4160  4.5280 
## 
## Coefficients:
##              Estimate Std. Error t value Pr(>|t|)    
## yd.lag       -0.47028    0.03581 -13.133  < 2e-16 ***
## yd.diff.lag1 -0.40473    0.03552 -11.395  < 2e-16 ***
## yd.diff.lag2 -0.30744    0.03334  -9.222  < 2e-16 ***
## yd.diff.lag3 -0.20795    0.02926  -7.108 1.63e-12 ***
## yd.diff.lag4 -0.09369    0.02223  -4.214 2.62e-05 ***
## ---
## Signif. codes:  0 '***' 0.001 '**' 0.01 '*' 0.05 '.' 0.1 ' ' 1
## 
## Residual standard error: 0.7735 on 2012 degrees of freedom
## Multiple R-squared:  0.436,  Adjusted R-squared:  0.4346 
## F-statistic: 311.1 on 5 and 2012 DF,  p-value: < 2.2e-16
## 
## 
## Value of test-statistic is: -13.133 
## 
## Critical values of DF-GLS are:
##                  1pct  5pct 10pct
## critical values -2.57 -1.94 -1.62
```

```
plot(ERS_test)
```

If for the PP, ADF, ERS test, the absolute values of the test
statistics are higher than the 0.05 critical value, we can conclude that
the residuals of the initials model shows difference stationarity.

#### 1.1.8.8 **Adapting the GAM() to correct for autocorrelation**

After checking for autocorrelation, (and if the sampling would have
been not equally spaced), we conclude that we should address the
autocorrelation in the residuals of the time series using the corARMA
argument of the *gamm* function (but we can´t). We might consider
differencing the data or adding an ARMA process with a moving average
and autoregressive process. We also used the restricted maximum
likelihood (REML) argument as the smoothness selection method, as it is
the preferred method in literature and less prone to overfitting (Reiss
and Ogden, 2009; Wood, 2011).

To address these contradicting results between the three unit root
tests, we performed an additional unit root test, the NP test
implemented in the *CADFtest* function from the package
R/CADFtest, that gives valid results of the ADF test regressions even if
the residuals follow an unknown ARMA process (Ng and Perron, 2001).
Again, the null hypothesis was that a unit root is present in the
residuals.

```
NP_test <- CADFtest(residuals(m10b), criterion = "MAIC", type = 'none')
NP_test
```

```
## 
##  ADF test
## 
## data:  residuals(m10b)
## ADF(1) = -24.55, p-value < 2.2e-16
## alternative hypothesis: true delta is less than 0
## sample estimates:
##      delta 
## -0.6853183
```

```
summary(NP_test)
```

```
## Augmented DF test 
##                                                 ADF test
## t-test statistic:                          -2.455042e+01
## p-value:                                    4.337019e-41
## Max lag of the diff. dependent variable:    1.000000e+00
## 
## Call:
## dynlm(formula = formula(model), start = obs.1, end = obs.T)
## 
## Residuals:
##     Min      1Q  Median      3Q     Max 
## -4.5808 -0.1788  0.0255  0.4926  4.2443 
## 
## Coefficients:
##            Estimate Std. Error t value Pr(>|t|)    
## L(y, 1)    -0.68532    0.02791 -24.550  < 2e-16 ***
## L(d(y), 1) -0.14396    0.02205  -6.529 8.36e-11 ***
## ---
## Signif. codes:  0 '***' 0.001 '**' 0.01 '*' 0.05 '.' 0.1 ' ' 1
## 
## Residual standard error: 0.7883 on 2018 degrees of freedom
## Multiple R-squared:  0.4124, Adjusted R-squared:  0.4119 
## F-statistic:    NA on NA and NA DF,  p-value: NA
```

```
NP_test$max.lag.y
```

```
## [1] 1
```

```
plot(NP_test)
```

If the absolute values of the test statistics are substantially
higher than the p-values, we can conclude that the residuals of the
models does not show difference stationarity.

To assess the required autoregressive and moving average order, we
used the *auto.arima* function with the arguments stationary =
TRUE and approximation = FALSE from the package R/forecast (Hyndman and
Khandakar, 2008).

```
arma <- auto.arima(residuals(m10b),
                        stationary = TRUE, approximation=TRUE, stepwise = TRUE, seasonal = FALSE)
arma
```

```
## Series: residuals(m10b) 
## ARIMA(5,0,0) with non-zero mean 
## 
## Coefficients:
##          ar1     ar2     ar3     ar4     ar5    mean
##       0.1212  0.0936  0.0957  0.1105  0.0899  0.0883
## s.e.  0.0221  0.0222  0.0222  0.0222  0.0222  0.0350
## 
## sigma^2 = 0.595:  log likelihood = -2341.35
## AIC=4696.71   AICc=4696.76   BIC=4735.99
```

Remember that a better model fit is the best way of accounting for
autocorrelation (rather than adding an ARMA process to the residuals).
The lack of data is not helping here… Including an ARMA process with
Less moving average or auto regressive terms is also preferred over
including moving average or autoregressive processes with higher order
terms.

### 1.1.9 **Cross-validating the model**

#### 1.1.9.1 **Searching for the RMSE of the location\_scale GAM**

We can calculate the extent of downward bias in the estimated
standard error of the residuals using the *CVgam* function in the
R/gamclass package. We specify the number of folds in the nfold
argument. The result should be a cross-validation estimate of the
residual mean square error (i.e. MSE; the standard deviation of the
residuals or the prediction error) (Maindonald, 2020). The purpose of
doing the cross-validation here is to discover whether we over fitted
the data (i.e. we compared the model performance with the model
performance of the full data).

GCV or the unbiased risk estimator (UBRE) are estimates for the MSE
in GAMs (Wood, 2017)

A smaller MSE value would suggest a more predictive GAM. ’The
CV-mse-GAM statistic in the right column is the estimate of the mean
square error given by ordinary cross-validation´ (OCV; (Maindonald,
2016). The issue with this method is that the cross-validation is only
applied to the location part of the location GAM. If the heterogeneity
in the model ends up affecting the estimated smooths in the
location-only model, the resulting MSE estimate is wrong.

The functions in Rmight possibly help.

```
model_performance(m10b)
```

```
## # Indices of model performance
## 
## AIC      |     AICc |       BIC | Nagelkerke's R2 |   RMSE | Sigma
## ------------------------------------------------------------------
## 6357.391 | 7272.996 | 10619.352 |           1.000 | 19.679 | 1.000
```

```
performance_mse(m10b, normalized = TRUE)
```

```
## [1] 7252.447
```

```
performance_rmse(m10b, normalized = TRUE)
```

```
## [1] 0.08338744
```

```
performance_mae(m10b, normalized = TRUE)
```

```
## Error in FUN(left, right): non-numeric argument to binary operator
```

What about the adjusted R

```
gamR2 <- function(gam){
R2 <- 1-((sum(residuals(gam)^2))/
(sum((gam$y - mean(gam$y))^2)))
R2adj <- 1- ((1 - R2) * (length(gam$y) - 1)/
(length(gam$y) - length(gam$coefficients) - 1))
a <- data.frame(R2, R2adj)
return(a)
}

gamR2(m10b)
```

```
##          R2     R2adj
## 1 0.9996486 0.9993573
```

Why is R2 so high? Does it indicate overfitting? It could… but
wouldn´t this be displayed as too much ´wiggliness´ in the smoothers?
What about the principle of Ocam’s razor? Maybe some remarks can be
found in Belkin et al. (2019), Belkin et al. (2020) and Belkin (2021)
stating that although hypothesis complexity may increase (aka the number
of parameters) one can still have low bias and variance (i.e. the
interpolation threshold). In fact, Belkin et al . (2020) states that
“infinite overparameterization can be preferable to any finite number of
parameters”. Also, overfitting would typically be considered a problem
when applying your model to new data but that wasn´t the purpose of the
question here…

### 1.1.10 **Limitations to the GAMM model and why go further with the analyses**

We performed extra tests to characterize our data.

#### 1.1.10.1 **Long range dependance**

We started by performing the Whittle test using the
*WhittleEst* function from the R/longmemo package (Beran, 1994;
Beran et al., 2020; Hurst, 1951). The Whittle test estimates the Hurst
coefficient and can be interpreted as an index of long-range dependence
that allows to assess the randomness of a system (Kleinow, 2002;
Mandelbrot and Hudson, 2004). For example, Hurst coefficients that are
lower than 0.5 but higher than 0 indicate that high values will be
followed by low values (i.e. anti-persistent), while Hurst coefficients
above 0.5 but below 1 indicate that high/low values will be followed by
high/low values (i.e. persistent). A Hurst parameter near 0.5 would be
considered a random walk with no clear trend (Barbulescu et al.,
2010).

```
Whittle_test <- WhittleEst(residuals(m10b))
Whittle_test$coef
```

```
##    Estimate Std. Error  z value Pr(>|z|)
## H 0.6365986 0.01441986 44.14736        0
```

```
confint(Whittle_test)
```

```
##       2.5 %   97.5 %
## H 0.6083362 0.664861
```

```
plot(Whittle_test)
```

#### 1.1.10.2 **The moments of the data’s distribution**

We characterized our Height data further by calculating the skewness
(i.e. the asymmetry around the mean of the probability distribution) and
kurtosis (i.e. the magnitude in which the tails of a distribution differ
from the tails of a normal distribution). The skewness and kurtosis, and
its standard errors, were calculated using the *skewness*,
*se.skew*, *kurtosis* and *describe* functions from
the packages R/e1071, R/sur and R/pscyh (Harrel, 2019; Meyer et al.,
2019; Revelle, 2019).

```
skewness(na.omit(data2$Height))
```

```
## [1] 0.647848
```

```
se.skew(na.omit(data2$Height))
```

```
## [1] 0.05326238
```

```
kurtosi(na.omit(data2$Height), na.rm=FALSE, type=3)
```

```
## [1] -0.06355977
```

```
describe(na.omit(data2$Height))
```

```
##    vars    n   mean    sd median trimmed   mad min max range skew kurtosis   se
## X1    1 2112 126.62 43.33    117  123.51 35.58  17 253   236 0.65    -0.06 0.94
```

#### 1.1.10.3 **Multimodality of the data’s distribution**

Finally, we tested the Height data for multimodality. First, we
performed unimodal test from the R/diptest package using the
*dip.test* function (Maechler, 2016).

```
dip.test(data2$Height)
```

```
## 
##  Hartigans' dip test for unimodality / multimodality
## 
## data:  data2$Height
## D = 0.0095881, p-value = 0.2298
## alternative hypothesis: non-unimodal, i.e., at least bimodal
```

Using the *bimodality\_amplitude* and *modetest*
functions, two multimodality tests from the R/modes and R/multimode
packages, we can find if our data is multimodal (Ameijeiras-Alonso et
al., 2018; Sathish and 4D Strategies, 2016).

```
#Dip test for unimodality
bimodality_amplitude(na.omit(data2$Height), fig = TRUE)
```

```
## [1] 0.184686
```

```
#Silverman's bimodality test
modetest(na.omit(data2$Height),mod0=1,method="SI")
```

```
## 
##  Silverman (1981) critical bandwidth test
## 
## data:  na.omit(data2$Height)
## Critical bandwidth = 13.221, p-value = 0.022
## alternative hypothesis: true number of modes is greater than 1
```

```
modetest(na.omit(data2$Height),mod0=2,method="SI")
```

```
## 
##  Silverman (1981) critical bandwidth test
## 
## data:  na.omit(data2$Height)
## Critical bandwidth = 4.9166, p-value = 0.872
## alternative hypothesis: true number of modes is greater than 2
```

### 1.1.11 **Conclusion**

’All models are wrong but some are useful (Box, 1976). Given a
limited sample size, the models made in this analysis are constrained
and subject to violations of their assumptions. Since all models are
wrong, the question becomes: what is the magnitude of the effect caused
by these violations of the assumptions on our outcomes? Key to this
issue is of course that experiments analysed using frequentist
statistics should be repeated. Only consistent findings will eventually
increase our certainty about the existence of a natural phenomena! We
could however improve our model fitting. Using a gamma distribution and
modeling more than just the mean improved our model fit and gave us a
model that violated less assumptions. We will continue here using the
R/gamlss framework.

### 1.1.12 **Making the GAMLSS model**

Often, the assumptions of Generalized additive (mixed) models (GAMMs)
are nevertheless violated in ecology due to limitation in the data
availability or experimental set-up. For example, the residuals of the
GAMMs can be non-independent and identically distributed (i.i.d.),
heteroscedastic and non-normal, while the data can show over-dispersion,
be skewed with heavy tails, or show multi-modality. A framework capable
of addressing these issues is GAMLSS (generalized additive models for
location, scale and shape) (Rigby and Stasinopoulos, 2005).

GAMLSS were introduced to model data where the distribution of the
response variable does not necessarily follows an exponential family
distribution (e.g. data that is discrete, censored, heterogeneous,
truncated, skewed or kurtotic, etc.; note that our data shows skewness,
kurtosis and autocorrelation) (Akanztiliotou et al., 2002; Rigby and
Stasinopoulos, 2001; Rigby and Stasinopoulos, 2005). Unlike GAMMs, the
GAMLSS inferential framework models not only the distribution parameter
µ, but also the distribution parameters σ, ν and τ. These four
distribution parameters correspond to the location, scale and shape of
the response variable’s distribution and can generally be interpreted
using the distribution’s moments (i.e. the mean, variance, skewness and
kurtosis, respectively) (Stasinopoulos and Rigby, 2007). In practice,
the semi-parametric GAMLSS framework can use many distribution families
to model the response variable whilst providing not only information on
changes in the mean but also on the variance, skewness and kurtosis
(Rigby et al., 2019).

Another advantage of the ‘complete distribution’ approach of GAMLSS,
unlike quantile regressions, is that it offers tools for both rigorous
testing of the parametric model assumptions and model selection
(Voudouris et al., 2013). However, the effectiveness of GAMLSS depends
largely on choices made by the user. For example, in GAMLSS, one has to
decide the distribution of the response variable, the link functions for
each parameter, the explanatory terms for each parameter and the amount
of smoothing (Voudouris et al., 2013).
